# Antagonizing microRNA‐19a/b augments PTH anabolic action and restores bone mass in osteoporosis in mice

**DOI:** 10.15252/emmm.202013617

**Published:** 2022-10-04

**Authors:** Hanna Taipaleenmäki, Hiroaki Saito, Saskia Schröder, Miki Maeda, Ramona Mettler, Matthias Ring, Ewa Rollmann, Andreas Gasser, Carl Haasper, Thorsten Gehrke, Alexander Weiss, Steffen K Grimm, Eric Hesse

**Affiliations:** ^1^ Molecular Skeletal Biology Laboratory, Department of Trauma, Hand and Reconstructive Surgery University Medical Center Hamburg‐Eppendorf Hamburg Germany; ^2^ Institute of Musculoskeletal Medicine, University Hospital LMU Munich Martinsried Germany; ^3^ Musculoskeletal University Center Munich, University Hospital LMU Munich Martinsried Germany; ^4^ Evotec SE Hamburg Germany; ^5^ Endo‐Clinic Hamburg Germany

**Keywords:** anti‐miRNA, osteoporosis, parathyroid hormone, Tgif1, treatment

## Abstract

Postmenopausal bone loss often leads to osteoporosis and fragility fractures. Bone mass can be increased by the first 34 amino acids of human parathyroid hormone (PTH), parathyroid hormone‐related protein (PTHrP), or by a monoclonal antibody against sclerostin (Scl‐Ab). Here, we show that PTH and Scl‐Ab reduce the expression of microRNA‐19a and microRNA‐19b (miR‐19a/b) in bone. In bones from patients with lower bone mass and from osteoporotic mice, miR‐19a/b expression is elevated, suggesting an inhibitory function in bone remodeling. Indeed, antagonizing miR‐19a/b *in vivo* increased bone mass without overt cytotoxic effects. We identified TG‐interacting factor 1 (Tgif1) as the target of miR‐19a/b in osteoblasts and essential for the increase in bone mass following miR‐19a/b inhibition. Furthermore, antagonizing miR‐19a/b augments the gain in bone mass by PTH and restores bone loss in mouse models of osteoporosis in a dual mode of action by supporting bone formation and decreasing receptor activator of NF‐κB ligand (RANKL)‐dependent bone resorption. Thus, this study identifies novel mechanisms regulating bone remodeling, which opens opportunities for new therapeutic concepts to treat bone fragility.

## Graphical abstract

Due to low bone mass, osteoporosis leads to fractures. Current drugs increase bone mass but have limitations. Thus, additional medicines are needed. Here we established anti‐sense oligonucleotide‐based microRNA‐19a/b inhibition to increase bone mass in osteoporosis by a novel dual mode of action.

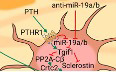

**The paper explained**

**Problem**

Postmenopausal osteoporosis affects millions of people worldwide and often leads to fragility fractures due to a decline in bone mass and structure. Fractures are associated with high morbidity and mortality and are caused by increased bone resorption and impaired bone formation. Current drugs increase bone mass and decrease fracture risk in an antiresorptive or anabolic manner. Although these drugs are potent, limitations and restrictions exist, and additional therapies are needed to better treat osteoporosis patients.

**Results**

We uncovered that the expression of endogenous microRNA‐19a/b (miR‐19a/b) in bone is reduced in response to anabolic treatment. Under physiological conditions, pharmacological inhibition of miR‐19a/b using an anti‐sense oligonucleotide increases bone mass in an anabolic manner. In the context of an activated bone remodeling, including sex steroid deficiency‐mediated osteoporosis, anti‐miR‐19a/b treatment increased the amount of newly formed bone but also inhibited the activated catabolic bone resorption, leading to a profound increase in bone mass and structure. This robust dual mode of action‐based pharmacological effect did not cause overt side effects and was overall well‐tolerated.

**Impact**

Our preclinical findings revealed a novel dual mode of action‐based therapy that could be used to improve the efficacy of existing drugs but also to serve as a novel therapy to treat osteoporosis in a well‐tolerated manner. Antisense oligonucleotide‐based miR‐19a/b inhibition is a novel treatment modality that is promising for drug development to obtain additional treatment options for patients with osteoporosis or other debilitating low bone mass diseases.

## Introduction

Osteoporosis is among the most prevalent diseases of the musculoskeletal system, both in women and men (Harvey *et al*, 2010; Gennari & Bilezikian, 2013; Seeman, 2013; Willson *et al*, 2015). Osteoporotic fractures often constitute life‐threatening events especially in the elderly and represent a significant socio‐economic burden. Bone is a highly dynamic tissue that is constantly dismantled and rebuilt throughout life by bone‐resorbing osteoclasts and bone‐forming osteoblasts. These processes are tightly coupled and balanced to maintain a stable bone mass (Baron & Hesse, 2012). Bone resorption followed by bone formation is the principle of bone remodeling. Bone modeling, however, is a resorption‐independent process by which bone formation occurs on quiescent surfaces without prior bone resorption. Both processes occur concomitantly in the bone tissue and contribute to bone mass maintenance (Baron & Hesse, 2012). In the context of sex steroid deficiency such as after menopause, bone resorption often increases while bone formation decreases (Raisz, 2005; Malluche *et al*, 2022). This leads to a reduction in bone mass and bone mineral density (BMD), frequently resulting in osteoporosis and fragility fractures.

Established antiresorptive drugs like bisphosphonates or a receptor activator of NF‐κB ligand (RANKL) neutralizing antibody (Denosomab) are therapeutic approaches that inhibit osteoclast activity, leading to an increase in BMD (Chen & Sambrook, 2011). By contrast, osteoanabolic therapies increase BMD by augmenting osteoblast activity and comprise the intermittent administration of a recombinant fragment containing the first 34 amino acids of human parathyroid hormone (PTH) (Teriparatide) or a modified form of the first 34 amino acids of the parathyroid hormone‐related protein (PTHrP) (Abaloparatide). Both drugs are agonists of the PTH type 1 receptor (PTH1R) (Bilezikian *et al*, 2009; Verhaar & Lems, 2010; Lombardi *et al*, 2011; Cusano & Bilezikian, 2012; Shirley, 2017). However, these treatments have several disadvantages, including discomfort due to daily injections, various contraindications, and a treatment period restricted to 24 months (Vahle *et al*, 2002). Activation of the canonical Wnt signaling pathway by antagonizing the Wnt inhibitor sclerostin using a monoclonal antibody (Romosozumab) is a novel approach to elevate bone mass and BMD (McClung *et al*, 2014; Cosman *et al*, 2017). Romosozumab is a potent drug for the treatment of patients with severe osteoporosis and a high risk of fractures. However, its use is recommended for only 12 months, and it should not be given to patients with an increased risk for cardiovascular events. Thus, the identification of additional targets to increase bone mass and BMD is very important, as this will advance our understanding of bone physiology and may provide new treatment options for patients with osteoporotic bone loss.

Recently, microRNAs (miRNAs) have gained great attention as novel therapeutic approaches to treat patients with cancer or infections with the hepatitis C virus (Iorio & Croce, 2012; Bouchie, 2013; Janssen *et al*, 2013). By altering transcript stability and/or by interfering with translation, miRNAs inhibit protein abundance and are established as key regulators of various biological processes including bone remodeling in health and disease (Taipaleenmäki *et al*, 2012; van Wijnen *et al*, 2013; Taipaleenmäki, 2018). Here, we identify miRNAs whose abundance is reduced during a bone anabolic response, with the aim to pharmacologically mimic this process by therapeutic manipulation of miRNAs. In this study, we describe miR‐19a and miR‐19b as novel mediators of the bone anabolic effects of both PTH treatment and activation of the canonical Wnt pathway.

MiR‐19a and miR‐19b arise from the highly conserved polycistronic cluster miR‐17~92 that contains miR‐17, miR‐18, miR‐19a, miR‐20a, miR‐19b, and miR‐192. Members of this cluster were shown to exert oncogenic functions and contribute to tissue homeostasis of the heart and the vascular system (Mendell, 2008; Concepcion *et al*, 2012; Peng *et al*, 2018; Li *et al*, 2019; Mayer *et al*, 2019). Recently, it has been reported that mice bearing a conditional deletion of the entire miR‐17~92 cluster targeted to osteoblasts show a reduced periosteal bone formation (Mohan *et al*, 2015). A series of miR‐17~92‐mutant mice and associated RNA‐seq data has been used to characterize the functional organization of this multifamily miRNA cluster (Han *et al*, 2015). Although several members of this cluster have been implicated in bone homeostasis, a distinct function of miR‐19a/b in bone remodeling in adult mice has not been reported.

We identified the homeodomain protein TG‐interacting factor 1 (Tgif1), a nuclear factor with positive effects on bone formation and a mediator of PTH anabolic signaling (Saito *et al*, 2019), as a downstream target of miR‐19a/b. Furthermore, we demonstrate that therapeutic inhibition of miR‐19a/b enhances the increase in bone mass in response to PTH therapy and reduces bone loss in mouse models of osteoporosis induced by sex steroid deprivation. Under these conditions of high bone turnover, anti‐miR‐19a/b functions in a dual mode of action by augmenting bone formation and attenuating an increase in RANKL expression and subsequent bone resorption. Finally, we show that miR‐19a and miR‐19b expression is increased in patients with relatively lower bone mass and under conditions of sex steroid deficiency. Taken together, inhibition of miR‐19a/b constitutes an innovative approach to enhance existing or develop novel therapies to improve the treatment options for patients suffering from osteoporosis.

## Results

### Inhibition of miR‐19a/b increases bone mass

**Figure 1:**
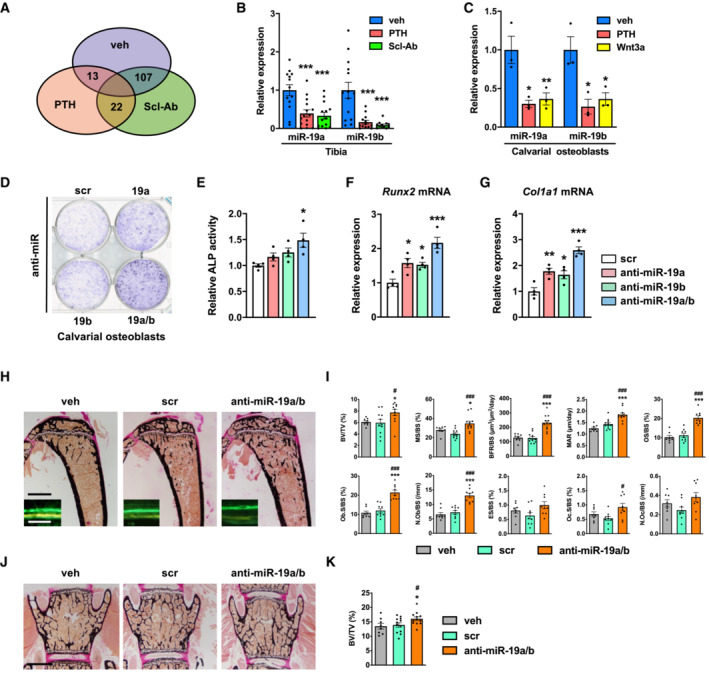
Inhibition of miR‐19a/b increases bone mass *in vivo* AVenn diagram illustrating the number of miRNAs whose expression was reduced in mouse bones *in vivo* upon injection of PTH (*n* = 4) or an antibody against Sclerostin (Scl‐Ab; *n* = 4) compared with vehicle (veh; *n* = 4) control.BQuantification of the expression of miR‐19a and miR‐19b in bones of mice treated with PTH (*n* = 15) or Scl‐Ab (*n* = 14) compared with veh (*n* = 13) control.CQuantification of the expression of miR‐19a and miR‐19b in calvarial osteoblasts upon *in vitro* stimulation with PTH or Wnt3a compared with veh control (*n* = 3).DAlkaline phosphatase staining of differentiating calvarial osteoblasts transfected with a scrambled (scr) control miRNA, anti‐sense microRNA (anti‐miR) inhibiting miR‐19a or miR‐19b or a combination thereof (anti‐miR‐19a/b) (representative image of 4 independent experiments).EQuantification of alkaline phosphatase staining of calvarial osteoblasts transfected with scr, anti‐miR‐19a, anti‐miR‐19b, or anti‐miR‐19a/b (*n* = 4).F, GExpression analysis of osteoblast marker genes (F) *Runx2* and (G) Type I Collagen (*Col1a1*) in calvarial osteoblasts transfected with scr, anti‐miR‐19a, anti‐miR‐19b, or anti‐miR‐19a/b (*n* = 4).HImages of von Kossa‐stained histological sections of the proximal tibiae of 12‐week‐old male C57Bl/6J mice and fluorescence double labeling to visualize bone formation (insets) after 4 weeks of treatment with anti‐miR‐19a/b (*n* = 12), scr control oligonucleotides (*n* = 11) or veh (*n* = 8). Scale bars indicate 1 mm (black) and 50 μm (white).IBone histomorphometric analysis of the proximal tibiae of the same animals as in (H). BFR/BS, bone formation rate/bone surface; BV/TV, bone volume/tissue volume; ES/BS, eroded surface/bone surface; MAR, mineral apposition rate; MS/BS, mineralizing surface/bone surface; N.Ob/BS, number of osteoblasts/bone surface; N.Oc/BS, number of osteoclasts/bone surface; Ob.S/BS, osteoblast surface/bone surface; Oc.S/BS, osteoclast surface/bone surface; OS/BS, osteoid surface/bone surface.JImages of von Kossa‐stained histological sections of the fourth lumbar vertebral body of 12‐week‐old mice after 4 weeks of treatment with anti‐miR‐19a/b, scr, or veh. Scale bar indicates 1 mm.KQuantification of bone mass (BV/TV) of the fourth lumbar vertebral body of 12‐week‐old mice after 4 weeks of treatment with anti‐miR‐19a/b (*n* = 12), scr (*n* = 12) or veh (*n* = 8). Data information: Mean values ± SEM. Three or four groups were compared using one‐way ANOVA followed by Tukey's *post hoc* analysis. *P < 0.05, **P < 0.01, ***P < 0.001 vs. veh, ^#^ *P* < 0.05, ^###^ *P* < 0.001 vs. scr.

**Figure EV1:**
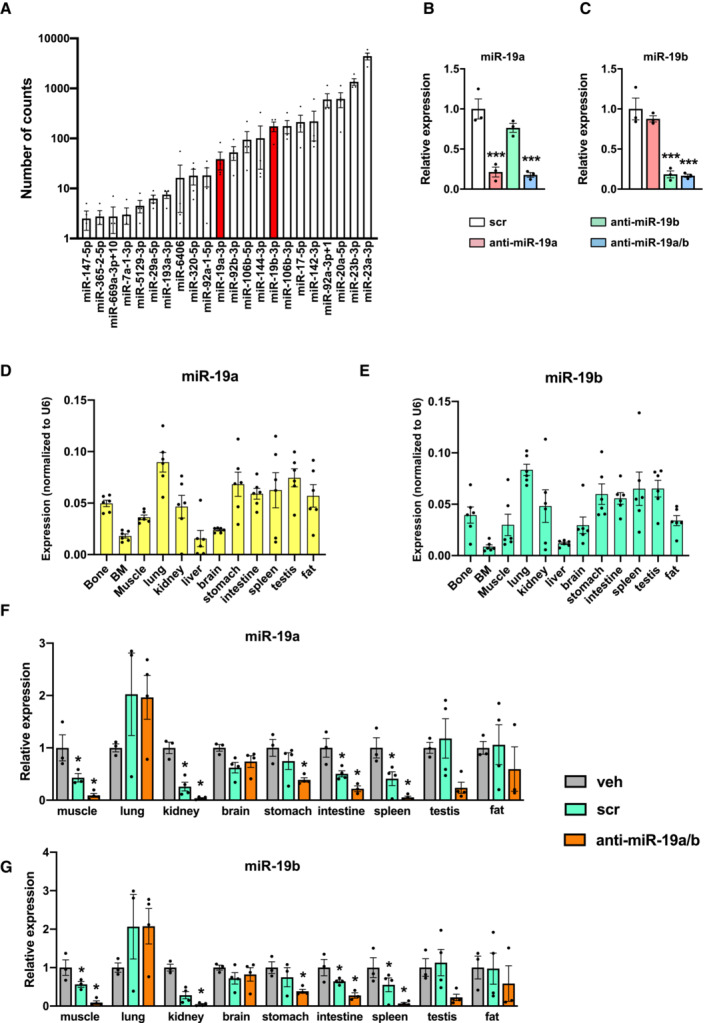
MiR‐19a and miR‐19b are expressed in various organs and downregulated by anti‐miR‐19a/b treatment AAbsolute expression (number of counts) of the 22 miRNAs in vehicle‐treated samples (*n* = 4) whose expression is decreased in mouse tibiae by PTH and Scl‐Ab treatment.B, CExpression of miR‐19a (B) and miR‐19b (C) in MC3T3‐E1 cells after transfection with scrambled (scr) control oligonucleotide, anti‐miR‐19a, anti‐miR‐19b, or anti‐miR‐19a/b (*n* = 3).D, EExpression of miR‐19a (D) and miR‐19b (E) in mouse organs and tissues (*n* = 6). Values are normalized to U6 as an internal control (Δ*C*_T_).F, GRelative expression of miR‐19a (F) and miR‐19b (G) in mouse organs and tissues after 4‐weeks of intravenous (i.v.) treatment with vehicle (veh, *n* = 3), scrambled (scr, *n* = 4) or anti‐miR‐19a/b (*n* = 4). Data information: Mean values ± SEM. One‐way ANOVA followed by Tukey's *post hoc* analysis was used to compare the groups. *P < 0.05 vs. veh. (F, G), ***P < 0.001 vs. scr (B, C).

To identify miRNAs as novel targets to increase bone mass, 8‐week‐old male mice were injected with a vehicle, a recombinant fragment containing the first 34 amino acids of human PTH (PTH) or an antibody against Sclerostin (Scl‐Ab). To identify potential therapeutically relevant candidates, we focused on miRNAs that were expressed at an intermediate level in the bone under steady‐state conditions to allow a sufficient downregulation and whose abundance was robustly reduced by both bone anabolic treatments. Activation of bone formation by PTH or Scl‐Ab treatment reduced the expression of 35 or 129 miRNAs in bone, respectively, of which 22 were downregulated by both treatments (Fig 1A). Among these miRNAs, we focused on miR‐19a and miR‐19b since quantification of gene expression demonstrated an intermediate basal abundance in bone (Fig EV1A) and confirmed the strong repressive function of PTH and Scl‐Ab treatment in tibiae *in vivo* (Fig 1B). Furthermore, activation of PTH and canonical Wnt signaling in calvarial osteoblasts *in vitro* using PTH and Wnt3a, respectively, confirmed the strong downregulation of miR‐19a and miR‐19b expression in an osteoblast‐autonomous manner (Fig 1C). These findings suggested that pharmacological inhibition of endogenous miR‐19a and miR‐19b might augment osteoblast differentiation. To address this hypothesis, we first transfected calvarial osteoblast with inhibitors of miR‐19a (anti‐miR‐19a), miR‐19b (anti‐miR‐19b), or a combination of miR‐19a and miR‐19b (anti‐miR‐19a/b) and confirmed a profound downregulation of endogenous miR‐19a and miR‐19b (Fig EV1B and C). Indeed, *in vitro* differentiation of calvarial osteoblasts was enhanced by transfection with anti‐miR‐19a and anti‐miR‐19b and more profoundly by concomitant antagonism of miR‐19a and miR‐19b using anti‐miR‐19a/b, demonstrated by an augmented alkaline phosphatase staining and an increase in alkaline phosphatase activity (Fig 1D and E). The enhanced osteoblast differentiation was further confirmed by an increased expression of the osteoblast‐related genes Runt‐related transcription factor 2 (Runx2) and Collagen type I alpha 1 (Col1a1) (Fig 1F and G). These findings suggested that treatment with anti‐miR‐19a/b might elicit a bone anabolic response *in vivo*.

Prior to investigating the effect of anti‐miR‐19a/b treatment *in vivo*, we quantified miR‐19a and miR‐19b expression and determined a broad expression pattern in various organs including bone (Fig EV1D and E). Next, we treated healthy 8‐week‐old male C57Bl/6J mice for 28 days with weekly intravenous injections of anti‐miR‐19a/b and observed a strong reduction in endogenous miR‐19a and miR‐19b expression in almost all organs investigated except in the lung, brain, testis, and fat (Fig EV1F and G). In support of our hypothesis, histomorphometric analysis and microcomputed tomography (μCT) revealed an increase in trabecular bone mass (bone volume per tissue volume; BV/TV) at the proximal tibia, lumbar spine, and distal femur as well as of the cortical density at the midshaft femur (Fig 1H–K; Appendix Fig S1A–D, Table EV1). Furthermore, parameters of bone formation (bone formation rate per bone surface; BFR/BS, mineral apposition rate; MAR and osteoid surface per bone surface; OS/BS) and the number and surface of osteoblasts per bone surface (N.Ob/BS, Ob.S/BS) were increased by anti‐miR‐19a/b treatment (Fig 1I, Table EV1). By contrast, although osteoclast surface (osteoclast surface per bone surface; Oc.S/BS) was increased by anti‐miR‐19a/b treatment, osteoclast number and activity (number of osteoclasts per bone surface; N.Oc/BS, eroded surface per bone surface; ES/BS) were not affected (Fig 1I). These findings demonstrate that under steady‐state conditions, antagonizing endogenous miRNA‐19a/b *in vivo* leads to an osteosclerotic increase in bone mass at the appendicular and axial skeleton.

### Administration of anti‐miR‐19a/b does not cause overt toxic effects

**Figure 2:**
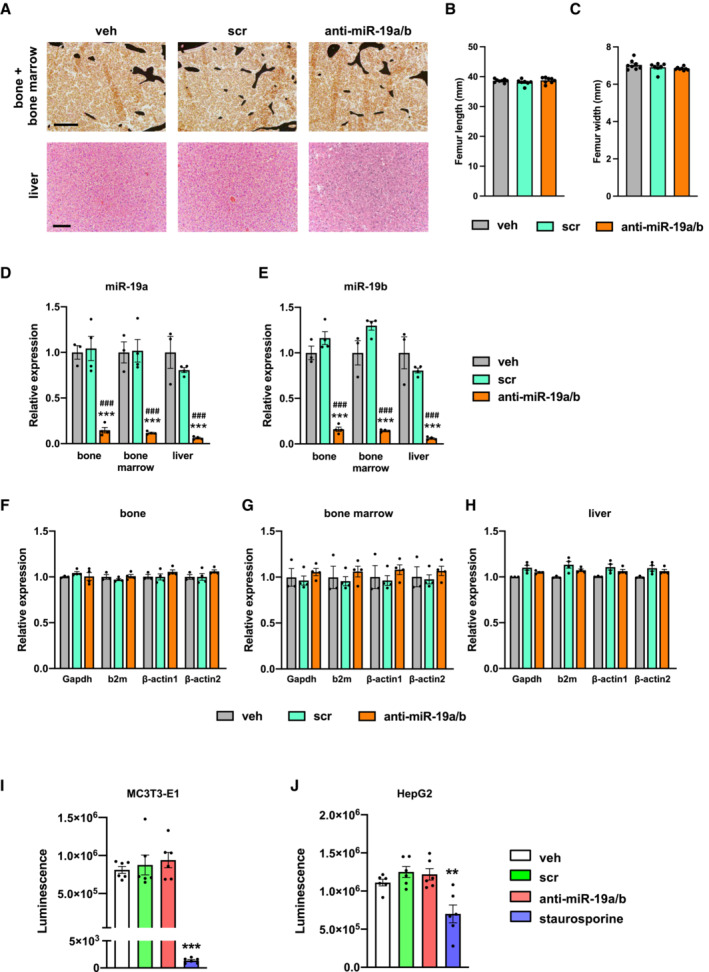
Anti‐miR‐19a/b treatment does not cause cytotoxic effects AImages of von Kossa‐stained histological sections of the bone and bone marrow (upper panel) and of hematoxylin–eosin‐stained liver sections (lower panel) of 12‐week‐old mice after treatment with anti‐miR‐19a/b (*n* = 12), scrambled (scr) control oligonucleotides (*n* = 11) or vehicle (veh) (*n* = 8). Scale bars indicate 100 μm (upper panel) and 50 μm (lower panel).B, CQuantification of the femur length (B) and width (C) after treatment with anti‐miR‐19a/b (*n* = 8), scr (*n* = 7) or veh (*n* = 8).D, EQuantification of the expression of miR‐19a (D) and miR‐19b (E) in the bone, bone marrow and liver after treatment with anti‐miR‐19a/b (*n* = 4), scr (*n* = 4) or veh (*n* = 3).F–HQuantification of the expression of the housekeeping genes glyceraldehyde‐3‐phosphate dehydrogenase (Gapdh), beta‐2 microglobulin (b2m), beta‐actin 1 (β‐actin 1) and beta‐actin 2 (β‐actin 2) in the bone (F), bone marrow (G) and liver (H) after treatment with anti‐miR‐19a/b (*n* = 4), scr (*n* = 4) or veh (*n* = 3).I, JCTG assay‐based quantification of luminescence signal intensity as a surrogate parameter of metabolically active osteoblasts of the MC3T3‐E1 cell line (I) and of hepatocytes of the HepG2 cell line (J) treated with anti‐miR‐19a/b, scr or veh and after stimulation with staurosporine to induce cell death as a positive control. *n* = 6. Data information: Mean values ± SEM. More than three groups were compared using one‐way ANOVA followed by Tukey's *post hoc* analysis. **P < 0.01, ***P < 0.001 vs. veh, ^###^ *P* < 0.001 vs. scr. Source data are available online for this figure.

**Figure EV2:**
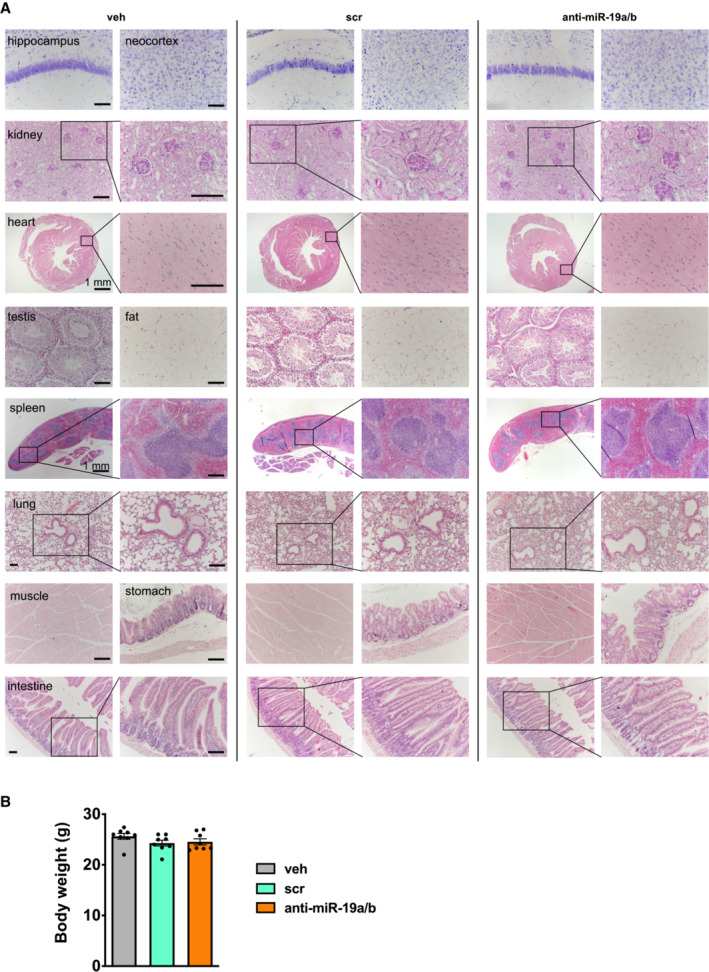
Histological analysis does not reveal overt adverse effects of anti‐miR‐19a/b treatment on tissue morphology Histology of various mouse organs as indicated after 4 weeks of treatment with vehicle (veh, *n* = 8), scrambled control oligonucleotide (scr, *n* = 11) or anti‐miR‐19a/b (*n* = 11). Organs were stained by Nissl stain (brain sections), with periodic acid–Schiff (PAS) (kidney sections), or with hematoxylin and eosin (all other tissues). Scale bars indicate 100 μm unless otherwise noted. Representative images are shown.Body weight of mice after 4 weeks of treatment with veh (*n* = 8), scr (*n* = 8) or anti‐miR‐19a/b (*n* = 8). Data information: Mean values ± SEM. One‐way ANOVA followed by Tukey's *post hoc* analysis was used to compare three groups.

**Figure EV3:**
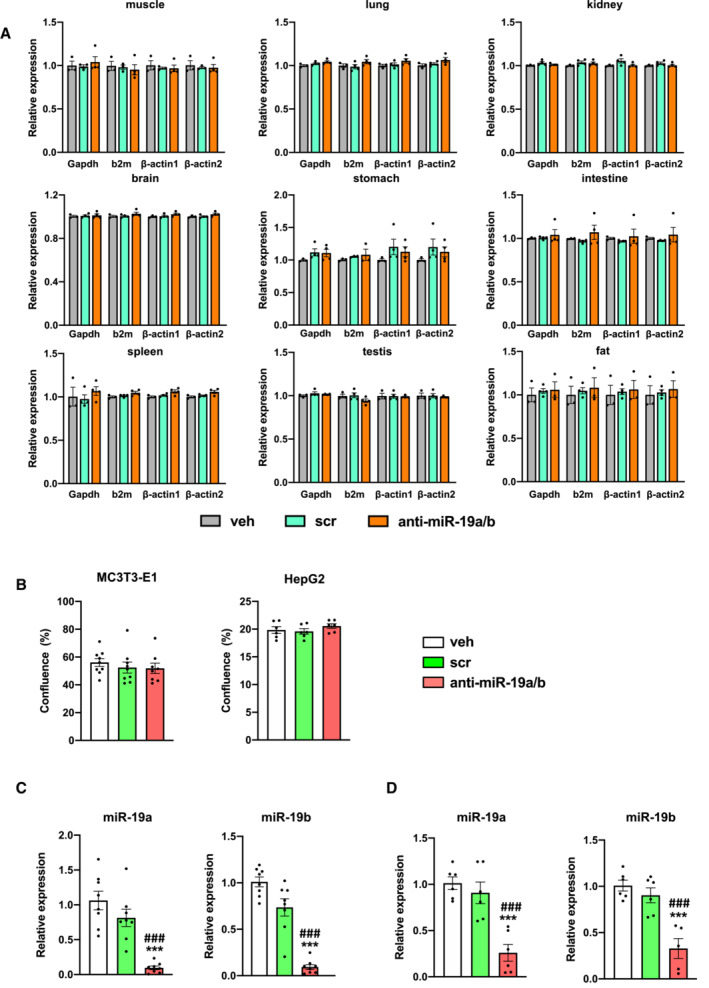
Gene expression and functional analysis do not indicate cytotoxic effects AQuantification of the expression of the housekeeping genes glyceraldehyde‐3‐phosphate dehydrogenase (Gapdh), beta‐2 microglobulin (b2m), beta‐actin 1 (β‐actin 1) and beta‐actin 2 (β‐actin 2) in several organs upon treatment with anti‐miR‐19a/b (*n* = 4), scrambled control oligonucleotide (scr, *n* = 4) or vehicle (veh, *n* = 3).BGrowth of osteoblasts of the MC3T3‐E1 cell line (*n* = 9) and of hepatocytes of the Hep2G cell line (*n* = 6) 24 h after treatment with anti‐miR‐19a/b, scrambled control oligonucleotide (scr) or vehicle (veh).C–D(C) Quantification of the expression of endogenous miR‐19a and miR‐19b in cells of the MC3T3‐E1 (*n* = 8) and (D) HepG2 (*n* = 6) cell lines after treatment with anti‐miR‐19a/b, scr or veh. Data information: Mean values ± SEM. Three groups were compared using one‐way ANOVA followed by Tukey's *post hoc* analysis. ***P < 0.001 vs. veh. ^###^ *P* < 0.001 vs. scr. Source data are available online for this figure.

A general concern often associated with miRNA mimic‐ or anti‐miR‐related therapies is the occurrence of organ‐damaging cytotoxic effects, particularly in the liver due to oligonucleotide accumulation. Given that endogenous miR‐19a/b is expressed in many organs (Fig EV1D and E) and since its expression is strongly reduced in most of them in response to anti‐miR‐19a/b treatment (Fig EV1F and G), we sought to determine whether anti‐miR‐19a/b causes cytotoxic effects. To address this question, we first performed a dedicated histological analysis of the bone, bone marrow, and liver of mice that presented with a high bone mass phenotype upon treatment with anti‐miR‐19a/b (Fig 1H–K; Appendix Fig S1A–D, Table EV1). No tissue abnormalities in bone, bone marrow, or liver or growth alterations affecting the length or width of the bones were detected (Fig 2A–C), while the efficacy of the treatment was confirmed by a reduced expression of endogenous miR‐19a/b (Fig 2D and E). To exclude potential cytotoxic effects in greater detail, we quantified the expression of the housekeeping genes glyceraldehyde‐3‐phosphate dehydrogenase (Gapdh), beta‐2 microglobulin (b2m), beta‐actin 1 (β‐actin 1) and beta‐actin 2 (β‐actin 2) in the bone, bone marrow, and liver but did not detect any changes in response to the administration of anti‐miR‐19a/b (Fig 2F–H). Next, we expanded these analyses to other organs and parameters obtained from the *in vivo* experiment and excluded adverse effects on tissue morphology of several organs, animal welfare, behavior, and body weight (Fig EV2A and B). Consistently, no changes in the expression of any housekeeping gene were found in any organ investigated (Fig EV3A), demonstrating that anti‐miR‐19a/b treatment does not cause any obvious organ damage *in vivo*.

To determine putative cell type‐specific cytotoxic effects, dedicated cell viability assays were performed. Briefly, cells of the osteoblast cell line MC3T3‐E1 and the liver cell line HepG2 were treated with anti‐miR‐19a/b. Staurosporine, a protein kinase inhibitor that causes apoptosis, served as a positive control. In cells of both cell lines, staurosporine induced a cytotoxic effect, while anti‐miR‐19a/b treatment did not affect cell viability (Fig 2I and J). Consistently, no change in the growth of osteoblasts or hepatocytes was observed due to anti‐miR‐19a/b treatment (Fig EV3B), which was effective as confirmed by the reduced expression of endogenous miR‐19a/b in cells of the MC3T3‐E1 and HepG2 cell lines (Fig EV3C and D). In summary, these findings demonstrate that inhibition of miR‐19a/b does not cause severe adverse effects *in vitro* or *in vivo* and represents a novel and specific treatment to increase bone mass.

### Anti‐miR‐19a/b treatment increases bone mass in a Tgif1‐dependent manner

**Figure 3:**
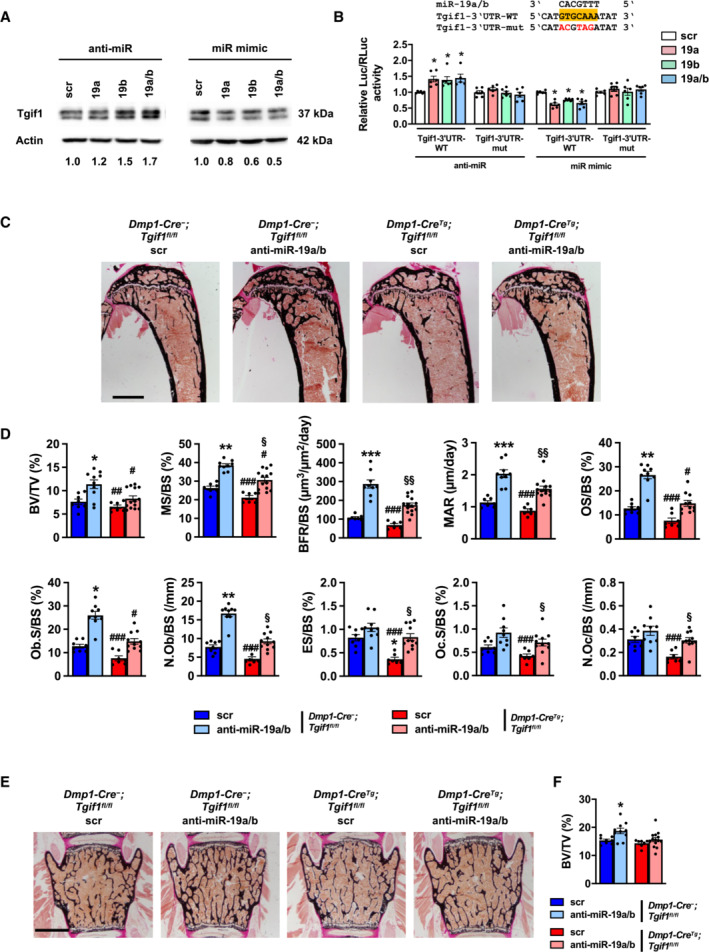
Anti‐miR‐19a/b increases bone mass in a Tgif1‐dependent manner AImmunoblot of Tgif1 protein expression in calvarial osteoblasts transfected with scrambled (scr), or with anti‐miR‐19a, anti‐miR‐19b and anti‐miR‐19a/b (left), or with miR‐19a, mir‐19b and mir‐19a/b mimics (right) as indicated. Immunoblot for Actin was used as a loading control. Normalized fold expression and molecular weight in kilo Dalton (kDa) are indicated (representative image of 3 independent experiments).BLuciferase activity in MC3T3‐E1 cells transfected with a reporter plasmid containing the 3′ untranslated region (UTR) of the wild‐type (WT) Tgif1 mRNA (Tgif1‐3′UTR‐WT) or an alternative version bearing mutations (mut, marked in red) of the putative miR‐19a/b binding site (yellow box) to disable the binding of miR‐19a/b (Tgif1‐3′UTR‐mut). In addition to the respective reporter plasmid, cells were co‐transfected with scr, or with anti‐miR‐19a, anti‐miR‐19b and anti‐miR‐19a/b (left), or with miR‐19a, mir‐19b and mir‐19a/b mimics (right) as indicated. *n* = 6.CImages of von Kossa‐stained histological sections of the proximal tibiae of 12‐week‐old male mice of the genotypes *Dmp1‐Cre‐;Tgif1* ^*fl*/*fl*^ treated weekly with scr (*n* = 8) or anti‐miR‐19a/b (*n* = 10) and *Dmp1‐Cre* ^*Tg*^ *;Tgif1* ^*fl*/*fl*^ treated weekly with scr (*n* = 7) or anti‐miR‐19a/b (*n* = 14) for 4 weeks. Scale bar indicates 1 mm.DBone histomorphometric analysis of the proximal tibiae of the same animals as in (C). For abbreviations see legend to Fig 1.E, FImages of von Kossa‐stained histological sections of the fourth lumbar vertebral bodies of the same animals as in (C) and (F) quantification of the BV/TV. Scale bar represents 1 mm. Data information: Mean values ± SEM. Statistical tests were performed with one‐way ANOVA followed by Tukey's *post hoc* analysis (B), or the nonparametric Kruskal–Wallis test to compare more than two groups (D, F), in which normal distribution could not be assumed. *P < 0.05, **P < 0.01, ***P < 0.001 vs. scr. Source data are available online for this figure.

In order to elucidate the molecular mechanism by which anti‐miR‐19a/b treatment increases bone mass; we performed an *in silico* analysis using TargetScan, miRWalk, and PicTar algorithms to identify miR‐19a/b targets. The significant top hundred conserved targets revealed by each algorithm were used to identify commonly predicted targets. These targets were ranked according to significance and novelty, leading to the identification of the homeodomain protein TG‐interacting factor 1 (Tgif1) as one of the predicted targets of miR‐19a/b with a conserved putative miR‐19a/b binding sequence in its 3′ untranslated region (UTR) (Appendix Fig S2). Functionally, regulation of Tgif1 expression by miR‐19a/b was confirmed by transfecting osteoblasts of the MC3T3‐E1 cell line with miR‐19a/b inhibitors or mimics, which increased or reduced Tgif1 protein abundance, respectively (Fig 3A). Consistently, the activity of a reporter gene construct containing the 3'UTR of the Tgif1 mRNA was increased or reduced by miR‐19a/b inhibitors or mimics, respectively, which was abolished by mutation of the miR‐19a/b binding site (Fig 3B), confirming that Tgif1 is indeed a direct target of miR‐19a/b.

Recently, we reported that Tgif1 promotes osteoblast function and bone remodeling (Saito *et al*, 2019), suggesting that Tgif1 might mediate the bone anabolic effect of anti‐miR‐19a/b. To test the hypothesis that Tgif1 in osteoblasts is important for the increase in bone mass in response to anti‐miR‐19a/b treatment, Tgif1 was deleted within the osteoblast lineage by crossing mice carrying the Tgif1 gene flanked by loxP sites (*Tgif1* ^*fl*/*fl*^) (Shen & Walsh, 2005) with mice expressing the Cre recombinase downstream of the Dentin matrix protein 1 (Dmp1) regulatory element (*Dmp1‐Cre* ^*Tg*^) (Bivi *et al*, 2012). Next, 8‐week‐old male animals were treated with miR‐19a/b inhibitors for 4 weeks. In mice lacking Tgif1 in mature osteoblasts (*Dmp1‐Cre* ^*Tg*^ *; Tgif1* ^*fl*/*fl*^), anti‐miR‐19a/b treatment had a less pronounced effect on increasing bone formation‐related parameters compared with *Dmp1‐Cre* ^*−*^ *; Tgif1* ^*fl*/*fl*^ controls while bone resorption started to become activated, thereby preventing a gain in bone mass in the tibia and the lumbar spine (Fig 3C–F; Table EV2). These findings demonstrate that anti‐miR‐19a/b increases bone mass through Tgif1 in mature osteoblasts and osteocytes.

### Antagonizing miR‐19a/b augments the bone anabolic effect of PTH

**Figure 4:**
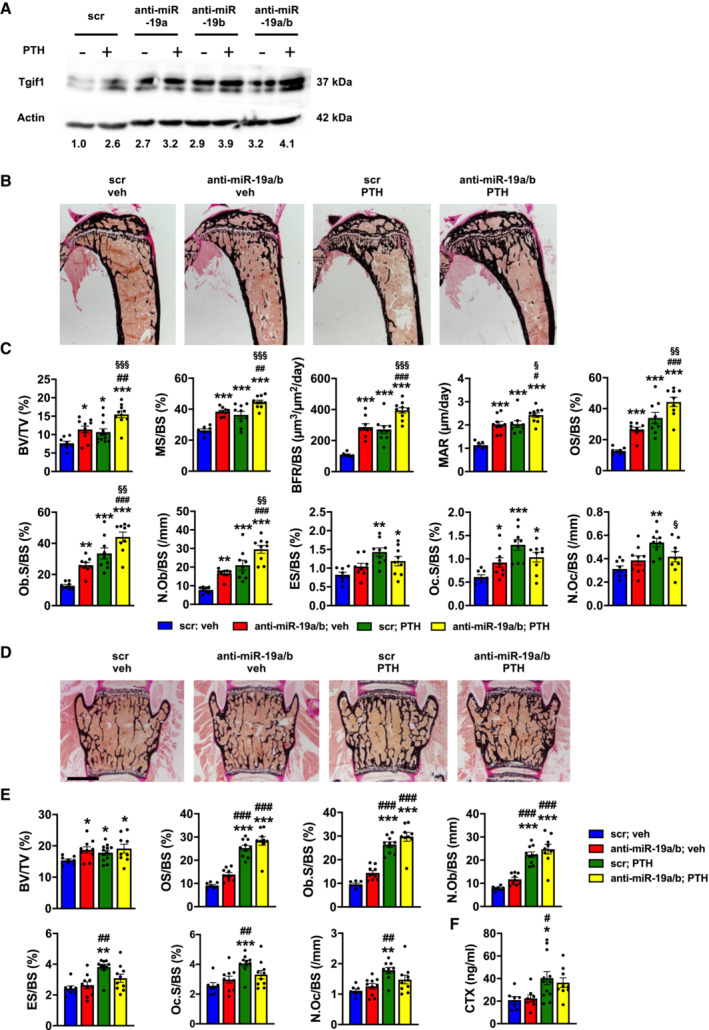
Inhibition of miR‐19a/b enhances the bone anabolic effect of PTH treatment Immunoblot of Tgif1 protein expression in calvarial osteoblasts after stimulation with PTH (+) or vehicle (−) and after transfection with scrambled (scr), anti‐miR‐19a, anti‐miR‐19b, or anti‐miR‐19a/b as indicated. Immunoblot for Actin was used as a loading control. Normalized fold expression and molecular weight in kilo Dalton (kDa) are indicated (representative image of 3 independent experiments).Images of von Kossa‐stained histological sections of the proximal tibiae of 12‐week‐old male wild‐type mice after treatment with scr oligonucleotides and vehicle (veh) (*n* = 8), anti‐miR‐19a/b and veh (*n* = 10), scr and intermittent PTH (*n* = 12) or a co‐treatment with anti‐miR‐19a/b and PTH (*n* = 10) for 4 weeks. Scale bar indicates 1 mm.Histomorphometric analysis of the proximal tibiae of the same animals as in (B) after treatment with intermittent PTH or veh and/or weekly injections of anti‐miR‐19a/b or scr control for 4 weeks. For abbreviations see legend to Fig 1.Images of von Kossa‐stained histological sections of the fourth lumbar vertebral bodies of the same animals as in (B). Scale bars indicate 1 mm.Histomorphometric analysis of the fourth lumbar vertebral body. For abbreviations see legend to Fig 1, scr, veh (*n* = 8); anti‐miR‐19a/b, veh (*n* = 10); scr, PTH (*n* = 12); anti‐miR‐19a/b, veh (*n* = 10).Analysis of serum carboxy‐terminal collagen crosslinks (CTX) in the same animals as in (B). Data information: Mean values ± SEM. One‐way ANOVA followed by Tukey's *post hoc* analysis was used to compare four groups. *P < 0.05, **P < 0.01 ***P < 0.001 vs. scr, veh; ^#^ *P* < 0.05, ^##^ *P* < 0.01, ^###^ *P* < 0.001 vs. anti‐miR‐19a/b, veh; ^§^ *P* < 0.001, ^§§^ *P* < 0.001, ^§§§^ *P* < 0.001 vs. scr, PTH. Source data are available online for this figure.

Tgif1 is a PTH target gene and is necessary to elicit the bone anabolic effect of PTH (Saito *et al*, 2019). Since both PTH and anti‐miR‐19a/b treatments increase Tgif1 expression and bone mass, we aimed to determine the effect of concomitant treatment. Interestingly, treatment with PTH alongside anti‐miR‐19a or anti‐miR‐19b further increased Tgif1 protein abundance compared with either treatment alone with the most profound effect being observed upon co‐treatment with PTH and anti‐miR‐19a/b (Fig 4A). Furthermore, anti‐miR‐19a/b treatment of 8‐week‐old male mice increased bone mass in the proximal tibia and the lumbar spine to a similar extent like PTH and augmented the bone anabolic effect of PTH in tibiae (Fig 4B–E).

PTH is well established to increase bone mass in a remodeling‐based manner by activating bone resorption and bone formation (Baron & Hesse, 2012), which was confirmed by histomorphometric analysis (Fig 4C and E; Table EV3). In detail, PTH increased all osteoblast‐related parameters like the osteoblast surface per bone surface (Ob.S/BS), the number of osteoblasts per bone surface (N.Ob/BS), and all indices of bone formation such as the mineralizing surface per bone surface (MS/BS), the bone formation rate per bone surface (BFR/BS), the mineral apposition rate (MAR) and the osteoid surface per bone surface (OS/BS). Activation of bone resorption was reflected by an increase in osteoclast surface per bone surface (Oc.S/BS), the number of osteoclasts per bone surface (N.Oc/BS), and the eroded surface per bone surface (ES/BS) (Fig 4C and E; Table EV3). By contrast, treatment with anti‐miR‐19a/b alone increased bone mass in a more modeling‐based manner, e.g., without activating bone resorption, by increasing all parameters of osteoblast function and bone formation (MS/BS, BFR/BS, MAR, OS/BS, Ob.S/BS, N.Ob/BS) and had a modest effect on increasing the surface of osteoclasts (Oc.S/BS) only in tibiae (Fig 4C and E; Table EV3). Interestingly, co‐treatment with PTH and anti‐miR‐19a/b was strongly synergistic in increasing all parameters of osteoblast function and bone formation in tibiae, while the PTH‐mediated activation of osteoclast function and bone resorption was greatly attenuated (Fig 4C and E; Table EV3). This unexpected finding demonstrates that under the condition of high bone turnover, anti‐miR‐19a/b treatment has a dual effect with increasing bone formation and reducing the activated bone resorption, leading to a profound increase in bone mass foremost in long bones in mice (Fig 4B–E; Table EV3). Attenuation of the PTH‐mediated increase in bone resorption by anti‐miR‐19a/b treatment was confirmed by fewer osteoclasts in bones identified by tartrate‐resistant acid phosphatase staining (TRAP) (Appendix Fig S3) and a reduced concentration of the bone resorption marker carboxy‐terminal collagen crosslinks (CTX) in the serum of mice (Fig 4F). This context‐dependent strong dual anabolic and antiresorptive function could be very beneficial for the treatment of patients with severe osteoporosis.

### Pharmacological inhibition of miR‐19a/b restores bone mass in osteoporosis

**Figure 5:**
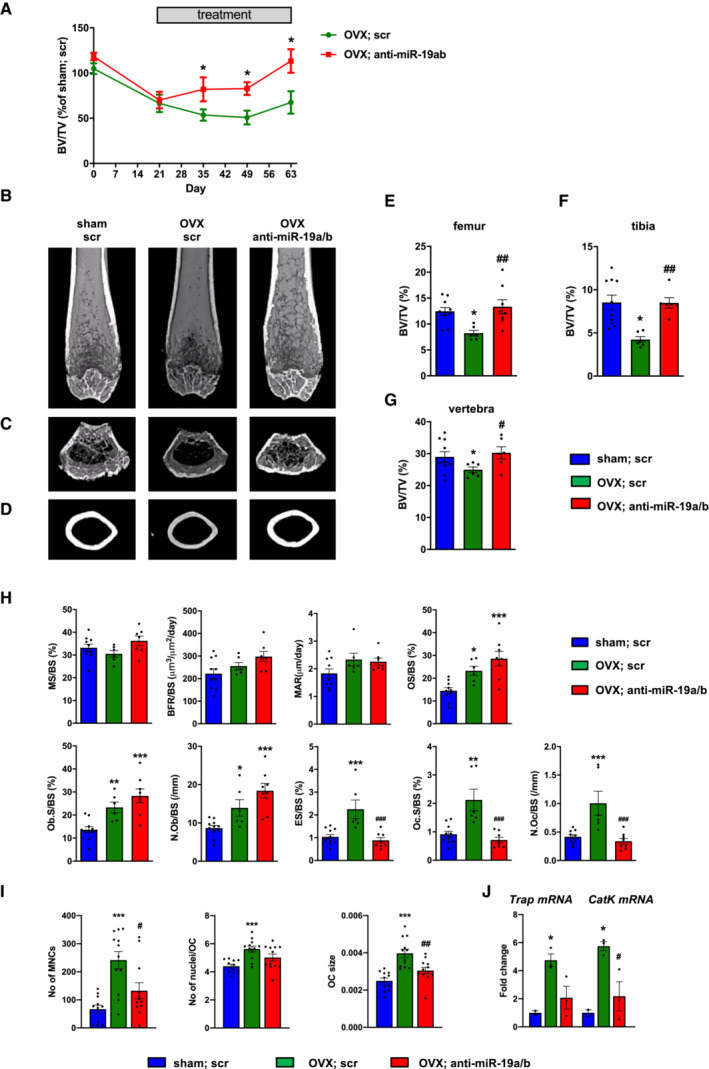
Inhibition of miR‐19a/b restores bone mass in a mouse model of osteoporosis ATime course of the relative change (compared with sham‐operated and scr‐treated control, *n* = 10) of bone mass (BV/TV, bone volume/tissue volume) in the tibiae of female mice in which osteoporosis was induced by ovariectomy (OVX) 21 days before the start of weekly intravenous (i.v.) treatment with scrambled (scr, *n* = 6) or anti‐miR‐19a/b (*n* = 8).B–DMicrocomputed tomography (μCT) of the distal femora (B, C) and midshaft femoral cross sections (D) of the same animals as in A, after the termination of the experiment.E–GQuantification of trabecular BV/TV by μCT in femora (E), tibiae (F), and the fourth lumbar vertebral bodies (G) of the same animals as in (A–D) 70 days after OVX.HHistomorphometric analysis of the proximal tibiae of the same mice as in (A–D) after termination of the experiment. For abbreviations see legend to Fig 1.IAnalysis of the number of TRAP‐positive multinucleated cells (No of MNCs), number of nuclei per osteoclast (No of nuclei/OC), and osteoclast size (OC size) after 4 days of *ex vivo* osteoclast differentiation of bone marrow macrophages isolated from sham‐operated (*n* = 11) or ovariectomized mice treated with scr (*n* = 12) or anti‐miR‐19a/b (*n* = 12).JExpression analysis of the osteoclast marker genes *Trap* and Cathepsin K (*CatK*) in bone marrow macrophages isolated from sham‐operated (*n* = 2) or ovariectomized mice treated with scr (*n* = 3) or anti‐miR‐19a/b (*n* = 3) after 4 days of differentiation. Data information: Mean values ± SEM. One‐way ANOVA followed by Tukey's *post hoc* analysis was used for statistical analysis. *P < 0.05, **P < 0.01, ***P < 0.001 vs. sham; scr, ^#^ *P* < 0.05, ^##^ *P* < 0.01, ^###^ *P* < 0.001 vs. OVX; scr.

**Figure EV4:**
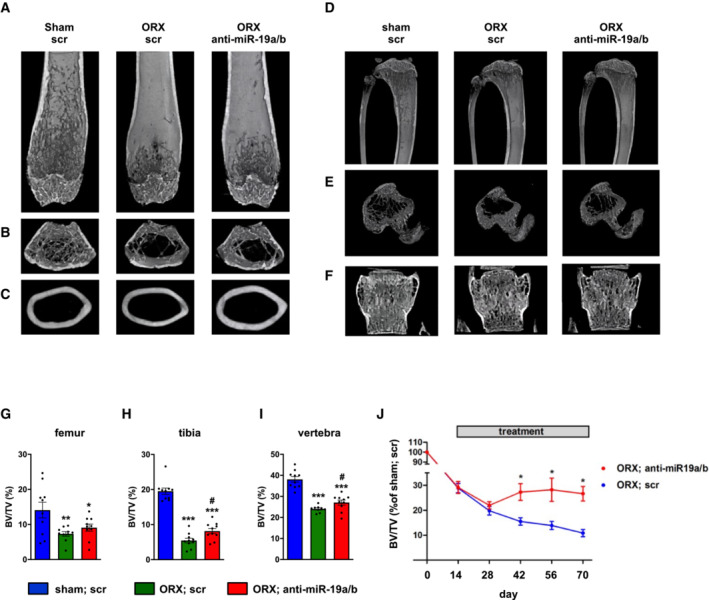
Anti‐miR‐19a/b treatment alleviates bone loss induced by orchiectomy A‐C(A, B) μCT scans of the distal femora and (C) of midshaft femoral cross sections of sham‐operated male mice treated with scrambled control oligonucleotides (scr, *n* = 10) and of male mice in which osteoporosis was induced by orchiectomy (ORX) 14 days before the start of weekly treatment with anti‐miR‐19a/b (*n* = 10) or scr (*n* = 10).D–F(D, E) μCT scans of the proximal tibiae and (F) the fourth lumbar vertebral bodies of the same mice as in (A–C).G–I(G) μCT‐based quantification of the trabecular bone mass (BV/TV, bone volume/tissue volume) in distal femora, (H) proximal tibiae, and (I) fourth lumbar vertebral bodies 70 days after ORX, sham, scr (*n* = 10); ORX, scr (*n* = 10); ORX, anti‐miR‐19a/b (*n* = 10).JTime course of the relative change (compared with sham‐operated and scr‐treated control) in BV/TV in the tibiae of male mice in which osteoporosis was induced by ORX 14 days before the start of weekly treatment with scr or anti‐miR‐19a/b, sham, scr (*n* = 10); ORX, scr (*n* = 10); ORX, anti‐miR‐19a/b (*n* = 10). Data information: Mean values ± SEM. One‐way ANOVA followed by Tukey's *post hoc* analysis was used for statistical analysis. *P < 0.05, **P < 0.01, ***P < 0.001 vs. sham; scr, ^#^ *P* < 0.05 vs. ORX; scr.

**Figure EV5:**
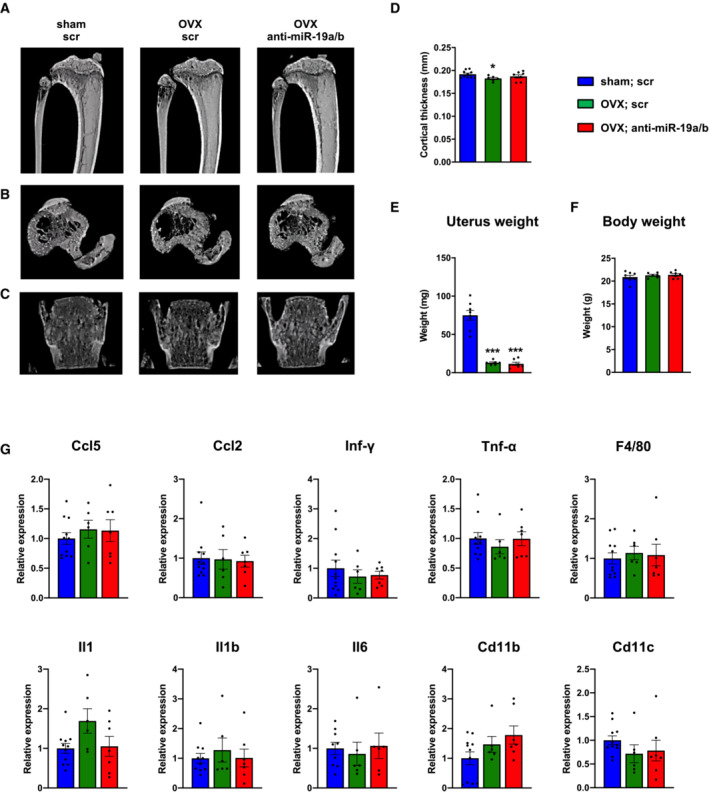
Anti‐miR‐19a/b treatment restores ovariectomy‐induced bone loss in mice A–C(A, B) μCT scans of the proximal tibiae and (C) the fourth lumbar vertebral bodies of female mice in which osteoporosis was induced by ovariectomy (OVX) 21 days before the start of weekly treatment with scrambled control oligonucleotides (scr, *n* = 6) or anti‐miR‐19a/b (*n* = 8). Sham‐operated and scr‐treated animals served as controls (*n* = 10).DHistomorphometric analysis of the cortical thickness at the midshaft femora of the same mice as in (A–C) after termination of the experiment.E, F(E) Uterus weight and (F) body weight of female mice after sham operation (*n* = 8) or ovariectomy (OVX) and 7 weeks of treatment with scr (*n* = 6) or anti‐miR‐19a/b (*n* = 7).GmRNA expression of inflammatory markers Ccl5, Ccl2, Interferon gamma (Inf‐γ), Tumor necrosis factor alpha (Tnf‐α), F4/80, Interleukin 1 (Il1), Interleukin 1b (Il1b), Interleukin 6 (Il6), cluster of differentiation 11b (Cd11b) and cluster of differentiation 11c (Cd11c) in tibiae of sham‐operated scr‐treated mice (*n* = 11) and OVX mice treated with scr (*n* = 6) or anti‐miR‐19a/b (*n* = 7). Data information: Mean values ± SEM. One‐way ANOVA followed by Tukey's *post hoc* analysis was used for statistical analysis. *P < 0.05, **P < 0.01, ***P < 0.001 vs. sham; scr, ^###^ *P* < 0.001 vs. OVX; scr.

To further explore the therapeutic potential of anti‐miR‐19a/b treatment in the context of metabolic bone disease, we induced sex steroid deficiency as an experimental model of osteoporosis in female and male mice. Orchidectomy in male mice caused a very rapid and severe trabecular bone loss in the femur, tibia, and lumbar spine and reduced the cortical thickness at the midshaft femur, with a partial but significant reconstitution of the trabecular bone mass by anti‐miR‐19a/b treatment (Fig EV4A–J; Table EV4). In female mice, ovariectomy significantly decreased bone mass 3 weeks after induction of the disease (Fig 5A). While trabecular bone mass continued to decline in osteoporotic mice treated with a scrambled control microRNA inhibitor, anti‐miR‐19a/b treatment fully restored the trabecular bone mass at all skeletal sites to the level of sham‐operated healthy control animals (Figs 5A–C and E–G, and EV5A–C; Table EV5). Cortical thickness at the midshaft femur was decreased due to ovariectomy and a partial reconstitution in response to anti‐miR‐19a/b treatment was noticed (Figs 5D and EV5D; Table EV5). At the tissue level, ovariectomy‐induced sex steroid deficiency increased all parameters of osteoclast function and bone resorption (N.Oc/BS, Oc.S/BS, ES/BS), thereby activating bone remodeling with a consecutive increase of some osteoblast‐related indices (N.Ob/BS, Ob.S/BS, OS/BS) (Fig 5H). In the context of this activated bone turnover, anti‐miR‐19a/b treatment of osteoporotic mice increased osteoblast parameters (N.Ob/BS, Ob.S/BS, OS/BS) but returned the increased osteoclast‐dependent bone resorption (N.Oc/BS, Oc.S/BS, ES/BS) back to the level of sham‐operated, scrambled‐treated control animals (Fig 5H).

Sufficient sex hormone deprivation in response to ovariectomy was confirmed by a reduced uterus weight, which was not affected by anti‐miR‐19a/b treatment (Fig EV5E). This finding suggests that restoration of the bone mass in osteoporotic mice is likely not mediated by an elevation of sex hormones. Consistent with earlier findings, no changes in body weight or adverse effects on several organs were observed in mice upon anti‐miR‐19a/b treatment (Fig EV5F; Appendix Fig S4).

Because sex steroid deficiency can activate low‐grade inflammation promoting the acute loss of bone mass in ovariectomized mice (Rogers *et al*, 2009; Cline‐Smith *et al*, 2020), we quantified the expression of a panel of inflammatory cytokines in the bone marrow but did not detect any alteration (Fig EV5G). Thus, neither the osteoporotic phenotype nor the pharmacological effect of anti‐miR‐19a/b treatment is influenced by inflammation.

The *in vivo* findings indicate that a reduction in increased osteoclast activity in the context of a pathologically elevated bone turnover might be the predominant effect of the anti‐miR‐19a/b‐mediated therapeutic increase in bone mass. To further investigate osteoclast function in this disease model, we obtained bone marrow macrophages (BMM) from ovariectomized mice followed by *ex vivo* osteoclast differentiation. While the number of multinucleated cells (No of MNCs), fusion to osteoclasts reflected by the number of nuclei per osteoclast (No of nuclei/OC), and the size of osteoclasts (OC size) obtained from ovariectomized mice was increased, treatment of ovariectomized mice with anti‐miR‐19a/b greatly reduced all parameters (Fig 5I). In addition, the expression of Tartrate‐resistant acid phosphatase (Trap) and Cathepsin K (CatK), markers of active bone‐resorbing mature osteoclasts, was increased in osteoclasts obtained from ovariectomized mice while anti‐miR‐19a/b treatment diminished the expression of both genes (Fig 5J). These findings support the notion that the increased differentiation and activity of osteoclasts obtained from ovariectomized mice are profoundly reduced by anti‐miR‐19a/b treatment.

**Figure 6:**
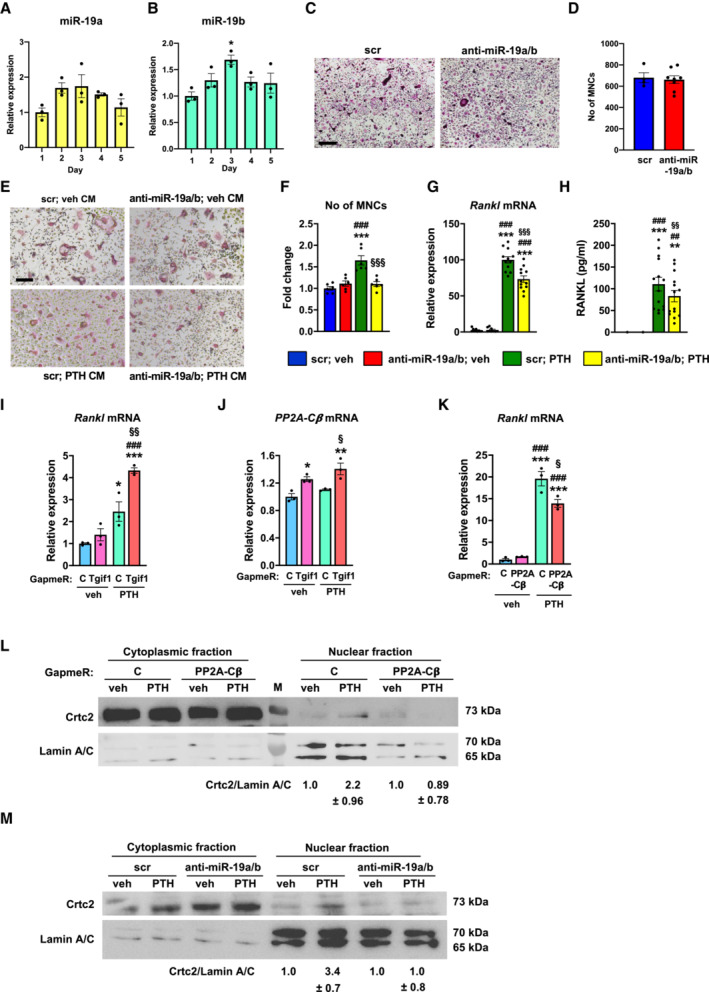
Anti‐miR‐19a/b suppresses osteoblast‐mediated osteoclast differentiation through inhibition of Rankl expression A, BExpression of miR‐19a (A) and miR‐19b (B) during osteoclast differentiation of bone marrow macrophages (*n* = 3).CRepresentative images of TRAP‐stained osteoclast cultures of bone marrow cells transfected with scrambled (scr) control oligonucleotide or anti‐miR‐19a/b (representative images of 3 independent experiments). Scale bar indicates 200 μm.DQuantification of multinucleated TRAP‐positive multinucleated cells (MNCs) after 4 days of osteoclast differentiation of bone marrow cells transfected with scr (*n* = 4) or anti‐miR‐19a/b (*n* = 8).E, FRepresentative images (E) and quantification of MNCs (F) after transfection with scr or anti‐miR‐19a/b and stimulation with conditioned medium (CM) collected from Ocy454 cells that were stimulated with vehicle (veh) or PTH (*n* = 6). Scale bar indicates 200 μm.GRankl mRNA expression in Ocy454 cells transfected with scr or anti‐miR‐19a/b and stimulated with veh or PTH (*n* = 12).HQuantification of RANKL protein abundance in CM collected from Ocy454 cells transfected with scr or anti‐miR‐19a/b and stimulated with veh or PTH (*n* = 14).I, JExpression of Rankl (I) and PP2A‐Cβ (J) mRNA in Ocy454 cells transfected with control GapmeR or GapmeR against Tgif1 and treated with veh or PTH (*n* = 3).KRankl mRNA expression in Ocy454 cells transfected with control GapmeR or GapmeR against PP2A‐Cβ and treated with veh or PTH (*n* = 3).LImmunoblot of Crtc2 protein expression in the cytoplasm and nucleus of Ocy454 cells transfected with control GapmeR or GapmeR against PP2A‐Cβ and stimulated with veh or PTH. Immunoblot for Lamin A/C was used as a loading control and purity control of the nuclear fraction. Normalized fold expression and molecular weight in kilo Dalton (kDa) are indicated (representative image of 3 independent experiments).MImmunoblot of Crtc2 protein expression in the cytoplasm and nucleus of Ocy454 cells transfected with scr or anti‐miR‐19a/b and stimulated with veh or PTH. Immunoblot for Lamin A/C was used as a loading control and purity control of the nuclear fraction. Normalized fold expression and molecular weight in kilo Dalton (kDa) are indicated (representative image of 3 independent experiments). Data information: Mean values ± SEM. The Student's *t*‐test was used for statistical analysis of two groups (D). One‐way ANOVA followed by Tukey's *post hoc* analysis was used for statistical analysis in all other experiments. *P < 0.05, **P < 0.01, ***P < 0.001 vs. Day 1 (B), vs. scr; veh (F–H), vs. scrambled control Gapmer (C); veh (K–L), ^##^ *P* < 0.01, ^###^ *P* < 0.001 vs. scr; veh (F–H), vs. C; veh (K–L), ^§^ *P* < 0.05, ^§§^ *P* < 0.01, ^§§§^ *P* < 0.001 vs. scr; veh (F–H), vs. C; veh (K–L). Source data are available online for this figure.

These observations lead to the unexpected notion that under conditions of activated bone remodeling like in response to PTH treatment (Fig 4C and E) but also upon ovariectomy (Fig 5H) as a model of sex steroid deficiency‐induced osteoporosis, but not under conditions of normal bone remodeling, the predominant effect of anti‐miR‐19a/b treatment on increasing bone mass is by attenuating the activated bone resorption. To further test this hypothesis, we first sought to rule out potential direct effects of anti‐miR‐19a/b on osteoclast differentiation because endogenous miR‐19a and miR‐19b are expressed in osteoclast precursor cells and increase during osteoclast differentiation (Fig 6A and B). While treatment with anti‐miR‐19a/b robustly reduced the abundance of endogenous miR‐19a and miR‐19b (Appendix Fig S5A), osteoclast differentiation was not affected as determined by TRAP staining and quantification of osteoclast number (Fig 6C and D). These observations excluded a direct effect of anti‐miR‐19a/b on osteoclasts. We therefore hypothesized that the inhibitory effect on bone resorption could be mediated indirectly through osteoblasts and osteocytes.

This thought was supported by the observation, that anti‐miR19a/b treatment of healthy mice with normal bone remodeling predominantly increased bone formation but not bone resorption (Figs 1I and 3D). However, upon targeted deletion of Tgif1 in cells of the osteoblast lineage, anti‐miR19a/b treatment increases bone formation and bone resorption, causing a high bone turnover phenotype with no gain in bone mass (Fig 3D). This suggests that anti‐miR19a/b treatment in the absence of Tgif1 stimulates osteoblast lineage cells to send signals to osteoclasts, thereby increasing bone resorption.

To investigate whether osteoblasts are the source of a signal that stimulates osteoclast differentiation, Ocy454 osteoblast lineage cells were transfected with anti‐miR‐19a/b or scrambled control oligonucleotides one day after plating. By day two, cells were treated with PTH or vehicle for 24 h, followed by harvesting conditioned medium (Appendix Fig S5B). A fraction of the conditioned medium was supplemented with RANKL and macrophage–colony‐stimulating factor (M‐CSF) and placed over BMMs obtained from adult female wild‐type mice. The medium was replaced by day 2 and MNCs were stained for TRAP and counted by day 4 (Appendix Fig S5B). Indeed, while conditioned medium obtained from osteoblasts transfected with anti‐miR‐19a/b did not affect the number of MNCs, medium harvested from osteoblasts upon treatment with PTH strongly increased the number of MNCs (Fig 6E and F). This effect was returned to control values by stimulating BMMs with medium collected from osteoblasts that were treated with anti‐miR‐19a/b and PTH (Fig 6E and F). These data strongly support the hypothesis that an osteoblast‐derived soluble factor supports osteoclast differentiation.

To identify the factor that stimulates osteoclast differentiation, we first quantified the expression of several molecules known to mediate osteoblast–osteoclast interactions including members of the ephrin family (Arthur & Gronthos, 2021), interleukins (Weitzmann, 2017), and insulin‐like growth factors and their binding proteins (Yakar *et al*, 2018). Anti‐miR‐19a/b did not revert any changes in gene expression induced by PTH treatment (Appendix Fig S5C), suggesting that it is unlikely that any of these factors is mechanistically involved. Given the established role of osteoblast‐ and osteocyte‐derived RANKL on osteoclast differentiation (Boyce, 2013), we quantified the expression of Rankl in Ocy454 cells stimulated with anti‐miR‐19a/b and PTH and a combination thereof. As expected, PTH robustly increased the expression of Rankl, which was significantly reduced by anti‐miR‐19a/b co‐treatment (Fig 6G). To confirm that changes in Rankl expression are also reflected by the amount of soluble RANKL in the conditioned medium, we quantified the concentration of RANKL in the supernatant. Consistent with the gene expression analysis, medium obtained from control osteoblasts or osteoblasts transfected with anti‐miR‐19a/b did not contain considerable amounts of RANKL compared with the conditioned medium harvested from osteoblasts that were stimulated with PTH (Fig 6H). Furthermore, concomitant treatment with anti‐miR‐19a/b caused a significant reduction in RANKL concentration (Fig 6H), which explains the decreased osteoclast differentiation.

Anti‐miR‐19a/b treatment reverts the PTH‐induced increase in Rankl expression (Fig 6G and H) and increases Tgif1 expression (Figs 3A and 4A). To further delineate the signaling pathway and to test the hypothesis that Tgif1 is implicated in the PTH‐dependent increase in Rankl expression, we reduced the expression of endogenous Tgif1 in Ocy454 osteoblast lineage cells using GapmeRs (Appendix Fig S5D) and stimulated these cells with PTH. Indeed, the PTH‐mediated increase in Rankl expression was further augmented by Tgif1‐deficiency, indicating that Tgif1 is functionally relevant to restrict the abundance of RANKL in the context of PTH stimulation (Fig 6I).

Protein Phosphatase 2A (PP2A) is a serine/threonine phosphatase that consists of structural‐, regulatory‐ and catalytic subunits (Kiely & Kiely, 2015). PP2A catalytic subunit isoform β (PP2A‐Cβ) is an important functional component of PPA, which contributes to the increase in Rankl expression in response to PTH treatment (Ricarte *et al*, 2018). We therefore investigated a functional involvement of PP2A‐Cβ. Tgif1 deficiency alone increased the expression of PP2A‐Cβ, which was further augmented by PTH stimulation (Fig 6J), demonstrating that Tgif1 is an upstream inhibitor of PP2A‐Cβ in osteoblasts. We then noticed that the PTH‐induced increase in Rankl expression was significantly reduced by PP2A‐Cβ‐deficiency (Fig 6K, Appendix Fig S5E), confirming that PP2A‐Cβ has a functional role in regulating Rankl expression.

It has been reported that PTH facilitates the nuclear translocation of the cAMP response element‐binding protein (CREB)‐regulated transcription coactivator 2 (Crtc2) to induce Rankl expression in response to PTH stimulation (Wein *et al*, 2016; Ricarte *et al*, 2018). Consistently, PP2A‐Cβ‐deficiency prevents the PTH‐mediated nuclear translocation of Crtc2 (Fig 6L). Since anti‐miR‐19a/b increases the abundance of Tgif1, which inhibits PP2A‐Cβ, we uncovered that anti‐miR‐19a/b treatment prevents the PTH‐mediated nuclear translocation of Crtc2 (Fig 6M), which is likely to prevent the increase in Rankl expression.

Together, these findings delineate the molecular pathway by which co‐treatment with anti‐miR‐19a/b and PTH reduces RANKL abundance via Tgif1‐PP2A‐Cβ‐Crtc2 signaling in osteoblasts, thereby attenuating bone resorption. This mechanism facilitates the increase in bone mass under conditions of high bone turnover.

**Figure 7:**
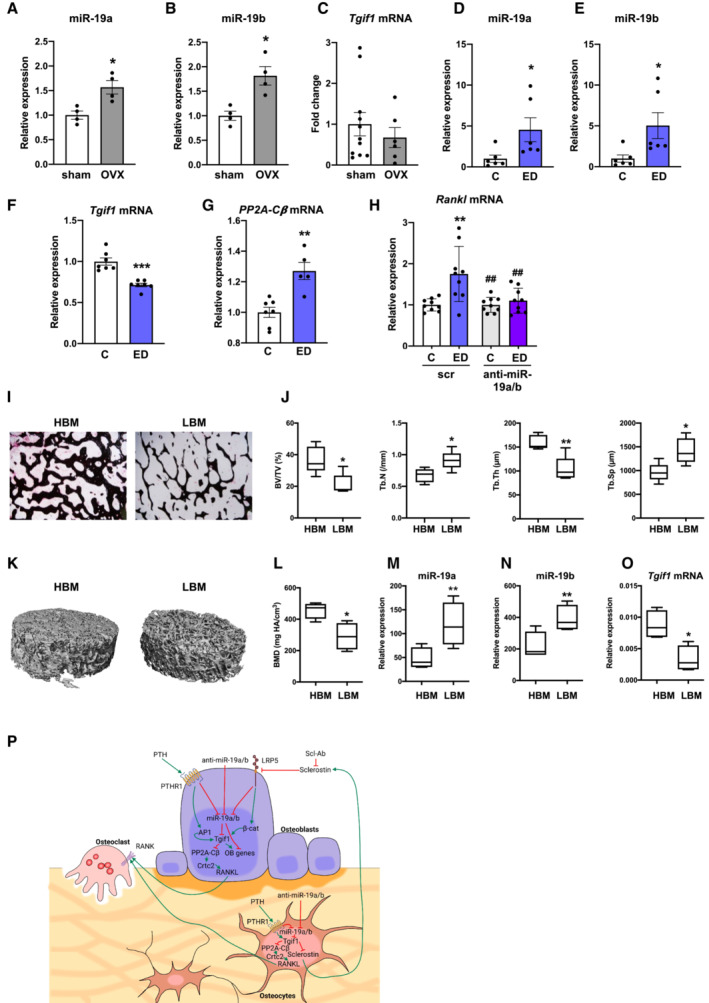
MiR‐19a/b expression is increased in osteoporotic bones from mice, due to *in vitro* sex steroid deficiency and in bones from humans with lower bone mass A, BRelative miR‐19a (A) and miR‐19b (B) expression in long bones of sham‐operated (*n* = 4) and ovariectomized (OVX) (*n* = 4) mice.CExpression of Tgif1 mRNA in long bones of sham‐operated (*n* = 11) and OVX (*n* = 6) mice.D, ERelative expression of miR‐19a (D) and miR‐19b (E) in Ocy454 cells upon estrogen deficiency (ED) and control (C) treatment (*n* = 6).F, GExpression of *Tgif1* (F) and *PP2A‐Cβ* (G) mRNA in Ocy454 cells after control in response to ED (*n* = 5–7).HExpression of *Rankl* mRNA in Ocy454 cells transfected with scrambled (scr) control oligonucleotide or anti‐miR‐19a/b in the context of ED (*n* = 9).IRepresentative images of von Kossa‐stained human bone samples obtained from nonfractured femoral heads of postmenopausal women after assignment to groups of relatively higher bone mass (HBM, *n* = 4) and relatively lower bone mass (LBM, *n* = 4).JHistomorphometric analysis of bone mass (BV/TV, bone volume/tissue volume), Trabecular number (Tb.N/mm), trabecular thickness (Tb.Th), and trabecular separation (Tb.Sp) of the same human bone samples as in (I).K, LRepresentative μCT images of 3D reconstructed human bone samples (K) and quantification of bone mineral density (BMD) (L). (HBM, *n* = 4), (LBM, *n* = 4).M, NRelative expression of miR‐19a (M) and miR‐19b (N) in association with higher and lower bone mass, (HBM, *n* = 4), (LBM, *n* = 4).OExpression of *Tgif1* mRNA in human bone samples, (HBM, *n* = 4), (LBM, *n* = 4).PSchematic model of the mechanism by which miR‐19a/b and anti‐miR‐19a/b treatment affects bone remodeling. Data information: Mean values ± SEM. In the box blots (J, L–O) the central band indicates the median, and the whiskers indicate the minimum and maximum values. One‐way ANOVA followed by Tukey's *post hoc* analysis was used for statistical analysis (H). Student's *t*‐test was used for statistical analysis of all other experiments. *P < 0.05, **P < 0.01, ***P < 0.001 vs. sham (A–C), vs. control (C) (D–G) vs. C; scr (H), vs. HBM (J–O); ^##^ *P* < 0.01 vs. ED; scr.

Given the profound effect of anti‐miR‐19a/b in restoring bone mass in osteoporosis, we reasoned that miR‐19a/b expression could be pathologically elevated in osteoporotic bones as part of the disease mechanisms. Indeed, expression of miR‐19a and miR‐19b was increased in bones from ovariectomized mice while the expression of Tgif1 had a trend to decrease compared with sham‐operated control animals (Fig 7A–C). Consistently, estrogen deficiency increased miR‐19a and miR‐19b expression in osteoblasts (Fig 7D and E). Similarly, miR‐19a and miR‐19b expression was increased by an androgen deficiency (Appendix Fig S6A and B). Mechanistically, Tgif1 expression was decreased while the expression of PP2A‐Cβ and Rankl was increased by estrogen and androgen deficiency (Fig 7F–H, Appendix Fig S6C and D). The elevated expression of Rankl caused by estrogen deficiency was returned to normal by anti‐miR‐19a/b treatment (Fig 7H). These data confirm, that anti‐miR‐19a/b reduces a pathologically increased Rankl expression in a Tgif1‐PP2A‐Cβ‐dependent manner.

Based on these findings, we hypothesized that miR‐19a/b expression could also be associated with bone mass in humans. To test this hypothesis, we obtained bone samples from postmenopausal women with relatively higher‐ and lower bone mass (Fig 7I–L). In support of our previous findings, expression of miR‐19a and miR‐19b was increased in bones from patients with lower bone mass compared with bones from patients with higher bone mass while the expression of Tgif1 was decreased (Fig 7M–O).

Together, these findings demonstrate that miR‐19a/b expression is increased in osteoblasts and osteocytes during high turnover bone remodeling. Under these conditions, administration of anti‐miR‐19a/b reduces the pathologically elevated bone resorption by preventing an increase in Rankl expression and augments bone formation in a dual mode of action, thereby normalizing bone mass in a Tgif1‐dependent manner without severe adverse effects (Fig 7P). This mechanism might also be implicated in the regulation of bone mass maintenance in humans and could serve as the basis for drug development.

## Discussion

Here we report that activation of the PTH and canonical Wnt signaling pathways decrease the expression of endogenous miR‐19a/b in the bone tissue. Furthermore, miR‐19a/b expression is elevated in bones from patients with a relatively low bone mass and under conditions of sex steroid deficiency including bones from osteoporotic mice. Pharmacological inhibition of miR‐19a/b increases bone mass under physiological conditions, enhances the gain in bone mass by PTH therapy, and reverts bone loss in gonadectomy‐induced mouse models of osteoporosis. No overt cytotoxic or *in vivo* adverse effects were determined. Thus, antagonizing endogenous miR‐19a/b represents a promising novel concept for the treatment of osteoporosis or other low bone mass diseases.

Recently, miRNAs have been proven to represent innovative pharmacological targets to treat diseases like cancer and hepatitis C (Iorio & Croce, 2012; Bouchie, 2013; Janssen *et al*, 2013). In this study, we extended this concept to bone. Drugs that increase BMD in patients with osteoporosis and lower the incidence of fragility fractures have several limitations and restrictions (Vahle *et al*, 2002; Bilezikian *et al*, 2009; Verhaar & Lems, 2010; Lombardi *et al*, 2011; Cusano & Bilezikian, 2012; McClung *et al*, 2014; Cosman *et al*, 2017; Shirley, 2017). We therefore sought to identify miRNAs as a novel molecular approach to augment bone mass in skeletal fragility. Given the emerging therapeutic impact of miRNAs, we aimed to identify miRNAs whose expression is decreased by known bone anabolic pathways, with the goal to pharmacologically mimic this downregulation to facilitate a gain in bone mass. Antagonizing endogenous miRNAs might presumably be a more specific approach than providing miRNA mimics, since anti‐sense miRNAs might only function in the presence of the endogenous miRNA target and might therefore cause less off‐site effects. Using the established bone anabolic capacity of the PTH and the canonical Wnt signaling pathways in an integrated manner facilitated the identification of miR‐19a/b as potential candidates.

Mimicking the reduction in endogenous miR‐19a/b expression using the systemic application of inhibitors against miR‐19a/b *in vivo* revealed a bone anabolic effect under steady‐state conditions. The effect of anti‐miR‐19a/b on bone remodeling involves the miR‐19a/b target Tgif1, a homeodomain protein and important regulator of bone remodeling (Saito *et al*, 2019). Deletion of Tgif1 in the osteoblast lineage impaired osteoblast function and reduced bone formation, representing the opposite effect of anti‐miR‐19a/b treatment. Consistent with this finding, antagonizing miR‐19a/b increased Tgif1 abundance and enhanced bone formation. Although miR‐19a/b has additional targets and not ruling out other Tgif1‐independent mechanisms, anti‐miR‐19a/b treatment failed to increase bone mass in the absence of Tgif1 in osteoblasts, indicating that Tgif1 is a crucial target of miR‐19a/b for bone mass accrual. These findings establish Tgif1 as a necessary component for the action of anti‐miR‐19a/b in bone. Similarly, germ‐line‐ and osteoblast‐targeted deletion of Tgif1 abrogated the PTH anabolic function (Saito *et al*, 2019) and our current findings suggest that Tgif1 is an essential factor interconnecting the PTH and miR‐19a/b pathways.

Important for clinical applications, we found that systemic inhibition of miR‐19a/b augments the increase in bone mass in response to PTH treatment. Although PTH is an efficient and overall safe drug, its use is restricted to 24 months (Vahle *et al*, 2002). Therefore, optimizing the currently available PTH therapy is not only of great scientific interest but would in fact provide a great clinical benefit. The strong synergistic effect of PTH and anti‐miR‐19a/b treatment indicates that miR‐19a/b is an important downstream component of the PTH signaling cascade, which opens several avenues to improve PTH therapeutic action. However, these findings do not exclude the possibility that PTH has miR‐19a/b‐independent effects and that anti‐miR‐19a/b has targets outside the PTH signaling pathway.

One of the features of PTH therapy is its mode of action, which is characterized by a remodeling‐based mechanism. PTH increases bone formation followed by the activation of bone resorption, thereby restricting the anabolic capacity of the drug. Interestingly, treatment of intact mice with anti‐miR‐19a/b alone increased bone formation and bone mass to a similar extent to PTH without affecting bone resorption. However, co‐treatment of mice with PTH and anti‐miR‐19a/b uncoupled bone formation from bone resorption during bone remodeling.

The increase in bone mass in response to PTH was weaker in the lumbar spine than in tibiae, which is opposite to the effect seen in humans and due to a less activated bone remodeling at this skeletal site in mice upon PTH stimulation (Iida‐Klein *et al*, 2002). This also explains the lack of an additive effect at the spine, while anti‐miR‐19a/b strongly augments PTH anabolic response in tibiae.

Interestingly, in the context of high bone turnover induced by PTH treatment or ovariectomy, anti‐miR‐19a/b functions in a dual mode of action by augmenting bone formation and attenuating the increased bone resorption. Mechanistically, antagonizing miR‐19a/b increases Tgif1, which inhibits PP2A‐Cβ, thereby alleviating the nuclear translocation of Crtc2 and subsequent Rankl expression. The decrease in secreted RANKL reduces osteoclast activation and bone resorption. However, under conditions of physiological bone remodeling and normal Rankl expression, anti‐miR‐19a/b treatment does not have an antiresorptive component. In consequence, anti‐miR‐19a/b renders PTH treatment more effective in a way that a greater amount of bone is gained during the same period of time, but anti‐miR‐19a/b can also be used alone to treat low bone mass conditions. These observations open promising clinical opportunities in the context of single, combined, or sequential therapies.

Expression of miR‐19a/b was increased in patients with a relatively lower bone mass and in osteoporotic mice and under conditions of *in vitro* sex steroid deficiency, suggesting that miR‐19a/b might be implicated in the regulation of bone mass maintenance in humans. Since miRNAs can be secreted into the circulation, they have clinical potential as noninvasive biomarkers in various diseases, including osteoporosis. Whether miR‐19a/b can be used in patient stratification to discriminate those with an increased risk of osteoporosis and fractures remains to be elucidated. Interestingly, a recent study identified an increased abundance of miR‐19a in the serum of rats after ovariectomy while the serum concentration of miR‐19a was decreased in response to PTH treatment (Kocijan *et al*, 2020). These findings are consistent with our data and strongly suggest that restoring the pathological elevation of miR‐19a/b might be a promising concept to treat osteoporosis.

Our findings demonstrate an increase in bone mass in response to anti‐miR‐19a/b treatment due to a dual mode of action implying an increase in bone formation and reduced bone resorption under high bone turnover conditions such as in mouse models recapitulating sex steroid deficiency‐induced osteoporosis (Zhou *et al*, 2018). However, this leaves open whether this therapeutic effect also improves bone quality. In addition, the models used here are limited because the animals were at 8 weeks of age, and therefore, relatively young age and future investigations using aged mice are likely to provide additional valuable information. Furthermore, a long‐term treatment and a targeting approach could be considered. Prior to transition into the clinics, proof of concept in larger animals, optimization of the dose, and route of administration would be required. Nevertheless, our findings clearly support this line of development and are encouraging to pursue the further translational exploitation of this innovation. This study clearly demonstrates that miR‐19a/b has an important function in physiological and pathological bone remodeling. We provide strong evidence that antagonizing miR‐19a/b is an innovative and overall safe concept to improve existing or to develop novel therapies to treat low bone mass diseases.

## Materials and Methods

### miRNA sequencing

For miRNA sequencing, 8‐week‐old male C57Bl/6J mice were injected with vehicle (veh; 0.1 mM acetic acid, 0.01% BSA in H_2_O), PTH 1–34 (100 μg/kg) or Scl‐Ab (100 μg/kg). After four (PTH and vehicle) or 24 h (Scl‐Ab and vehicle), total RNA, including small RNAs, was isolated from mouse bones after flushing the bone marrow using Trizol (Invitrogen) according to instructions provided by the manufacturer. Next Generation Sequencing (NSG) of miRNAs, including RNA quality control, library preparation, and downstream analysis was performed by Exiqon. MicroRNA sequencing was performed using the Illumina Platform.

### Cell culture

The osteoblast cell line MC3T3‐E1 and the human liver cancer cell line HepG2 were purchased from ATCC. The Ocy454 cell line was kindly provided by Dr. Divieti Pajevic. MC3T3‐E1 and Ocy454 cells were cultured in α‐MEM (Gibco) supplemented with 10% Fetal Bovine Serum (FBS, Gibco) and 1% Penicillin–Streptomycin (P/S, Gibco). HepG2 cells were cultured in DMEM (Gibco), 10% FBS, and 1% P/S. In various experiments, Ocy454 cells were stimulated with vehicle (0.1 mM acetic acid, 0.01% BSA in sterile water) or PTH (100 nM) for 4 h. To collect conditioned medium (CM), cells were transfected with scrambled control oligonucleotide (scr) or anti‐miR‐19a/b (final concentration 40 nM). One day after transfection, cells were treated with vehicle or PTH (100 nM) for 24 h. CM was diluted by 50% with α‐MEM supplemented with 10% FBS, 1% P/S, receptor activator of NF‐κB ligand (RANKL) (50 ng/ml) (Peprotech), and macrophage–colony‐stimulating factor (M‐CSF) (25 ng/ml) (Peprotech) prior to stimulating bone marrow macrophage (BMMs) to differentiate into osteoclasts. All cell lines used are regularly tested for mycoplasma contamination.

### *Ex vivo* osteoblast differentiation assays

For calvarial osteoblast cultures, calvariae were dissected from 1 to 3‐day old C57Bl/6J mice and digested sequentially in α‐MEM containing 0.1% collagenase and 0.2% dispase (both Roche). Cell fractions two to four were combined and expanded in α‐MEM containing 10% FBS and P/S. Osteoblast differentiation was induced by supplementing α‐MEM with 0.2 mM L‐ascorbic acid and 10 mM β‐glycerophosphate (both Millipore). Osteoblast differentiation was determined by alkaline phosphatase (ALP) staining after fixing the cells in 4% neutrally buffered formaldehyde solution. For ALP staining, cells were incubated with naphthol AS‐MX/Fast Blue (both Sigma‐Aldrich) in Tris–HCl solution for 15 min at room temperature.

### Gene and miRNA expression analyses

Total RNA was isolated from mouse bones and various tissues using Trizol reagent (Invitrogen) and from cultured cells using the RNEasy Plus Mini kit (Qiagen) according to the manufacturer's instructions. cDNA was synthesized from 1 μg of total RNA using ProtoScript First Strand cDNA Synthesis Kit (NEBioLabs). Quantitative real‐time PCR was performed using SYBR Green (BioRad). After normalization to TATA‐binding protein (Tbp) mRNA, relative expression levels and fold induction of each target gene (Table EV6) were calculated using the comparative *C*_T_ (ΔΔ*C*_T_) method. Small RNAs were isolated from various mouse tissues using Trizol. For the isolation of small RNAs from cells, the miRNEasy kit (Qiagen) was used. The QuantimiR‐kit (SBI System Biosciences) was used to add a polyA tail to small RNAs for cDNA synthesis according to the manufacturer's guidelines. Relative miRNA expression was determined by SYBR Green using a universal reverse primer and a specific forward primer designed for each miRNA of interest (Table EV6). U6 expression was used as internal control and the relative miRNA expression was calculated using the Δ*C*_T_ or ΔΔ*C*_T_ method.

### Animal experiments

Female and male C57Bl/6J wild‐type mice were purchased from Janvier Laboratories. To delete Tgif1 in osteoblasts *in vivo*, mice expressing the Cre recombinase under the control of the 8 kb fragment of the murine Dentin matrix protein 1 (*Dmp1‐Cre* ^*Tg*^)^8^ promoter were crossed with mice in which exons 2 and 3 of the Tgif1 gene are flanked by loxP sites (*Tgif1* ^*fl*/+^)^3^. The resulting mice with the genotype *Dmp1‐Cre* ^*Tg*^ *;Tgif1* ^*fl*/+^ were mated with *Tgif1* ^*fl*/+^ mice to obtain *Dmp1‐Cre* ^*Tg*^ *;Tgif1* ^*fl*/*fl*^ mice that bear a conditional deletion of Tgif1 in osteoblasts. Since no bone phenotype was observed in *Dmp1‐Cre* ^+^ mice, *Dmp1‐Cre* ^*−*^ *;Tgif1* ^*fl*/*fl*^ mice were used as control. For bone anabolic studies, a recombinant fragment containing the first 34 amino acids of human Parathyroid hormone (PTH; 100 μg/kg of body weight, Biochem) or control vehicle (veh; 0.1 mM acetic acid, 0.01% bovine serum albumin [BSA] in H_2_O) were administered intraperitoneally in 8‐week‐old male C57Bl/6J mice five times a week for 4 weeks. Locked nucleic acid (LNA)‐modified scrambled control miRNA inhibitors (scr) and inhibitors against miR‐19a/b (anti‐miR‐19a/b) were purchased from Exiqon/Qiagen. In 8‐week‐old male C57Bl/6J, *Dmp1‐Cre* ^*−*^ *;Tgif1* ^*fl*/*fl*^ or *Dmp1‐Cre* ^*Tg*^ *;Tgif1* ^*fl*/*fl*^ mice, oligonucleotides were injected at a concentration of 10 mg/kg intravenously (i.v.) once a week for 4 weeks. Orchiectomy or sham operation was performed in 8‐week‐old male C57Bl/6J wild‐type mice. Two weeks after orchiectomy, mice were treated with scr (10 mg/kg, i.v.) or anti‐miR‐19a/b (10 mg/kg, i.v.) for 7 weeks as described above. For ovariectomy, 8‐week‐old female C57Bl/6J mice were ovariectomized or sham‐operated and treated with scr (10 mg/kg, i.v.) or anti‐miR‐19a/b (10 mg/kg, i.v.) after 3 weeks for 7 weeks. All experiments were conducted according to the protocols approved by the local authority for animal welfare.

### Bone analyses

Mice were injected 7 and 2 days before sacrifice with calcein (20 mg/kg) and demeclocycline (20 mg/kg; both Sigma‐Aldrich), respectively. Tibiae, femura, and the fourth lumbar vertebral bodies (L4) were collected and fixed in 3.7% PBS‐buffered formaldehyde. For histomorphometric analysis, tibiae and L4 were embedded in methylmethacrylate. Toluidine blue, von Kossa, and Tartrate‐resistant acid phosphatase (TRAP) staining were performed using 4 μm sagittal sections. For analysis of dynamic bone parameters, unstained 4 μm sagittal sections were used. Quantitative bone histomorphometric measurements were performed according to standard protocols using the OsteoMeasure system (OsteoMetrics). Microcomputed tomography (μCT) was used for three‐dimensional analyses of bones. Long bones and L4 were analyzed using high‐resolution μCT with a fixed isotropic voxel size of 10 μm (70 kV at 114 μA, 400 ms integration time; Viva80 micro‐CT; Scanco Medical AG). The threshold value was determined at 326 mg/cm^3^ hydroxyapatite based on Hounsfield units and a phantom with a linear hydroxyapatite gradient (79–729 mg/cm^3^). All analyses were performed on digitally extracted bone tissue using 3D distance techniques (Scanco Medical AG). For analysis by histomorphometry and μCT, a region starting 200 μm below the growth plate of the long bones was used. For histomorphometry, the average size of the region of interest was 2.5 mm^2^. Length and width of the left femora were determined using high‐resolution *ex vivo* μCT analyses (Procon CT‐alpha, ProCon X‐Ray GmbH, Sarstedt, Germany) with the following settings: 150 kV, 100 μA, 0.4 s exposure time, 1,500 projections, 1 averaging, 2 × 2 binning mode and 22 μm voxel size. 3D images were generated using X‐AID reconstruction software (MITOS GmbH, Munich, Germany). Reconstructed images were further processed using the analysis software Dragonfly (ORS, Montreal, Canada). Length of each femur was determined by measuring the distance from the greater trochanter to the surface of the condyles. Width of each femur was determined by measuring the distance between the outer surfaces of the medial and lateral condyle.

### Histological analyses of mouse tissues

Soft tissues were collected and fixed overnight in 4% Formalin/PBS at 4°C. Tissues were dehydrated, embedded in paraffin, and cut into 4 μm thick sections. Hematoxylin–eosin staining was performed for the analysis of tissue histology. Brain sections were stained with Nissl stain, and kidneys were stained with Periodic acid–Schiff (PAS) stain. At least two investigators analyzed the tissue histology independently and in a blinded manner. Images of stained tissues were acquired using the Olympus DP72 microscope and CellSens Entry‐imaging program (Olympus Life Science).

### Cell viability assay

To assess putative cytotoxic effects of anti‐miR‐19a/b treatment, MC3T3‐E1 and HepG2 cells were transfected with anti‐miR‐19a/b or scrambled (scr) control oligonucleotides at a final concentration of 40 nM using Lipofectamine 3000 (Invitrogen; MC3T3‐E1 cells, forward transfection) and Lipofectamine RNAiMax (Invitrogen; HepG2 cells, reverse transfection) according to the manufacturer's instructions. Staurosporine (1 μM) was used to induce cell death as a positive control. Cell viability was determined 24 h after transfection using the CellTiter‐Glo (CTG) Assay (Promega). In this assay, a luminescence signal is generated that is proportional to the amount of ATP present in the cell, thereby acting as a surrogate parameter of metabolically active cells. Upon lysing the cells in buffer (150 nM NaCl, 20 mM Tris–HCl pH 7.5, 1 mM EDTA, 1 mM EGTA, 1% Triton X‐100), quantification of the luminescence signal by the CTG assay was performed according to the instructions provided by the manufacturer with the exception that an aliquot and not the entire lysate was used. Cell confluence was determined using the IncuCyte S3 Live‐Cell Analysis System (Sartorius) as recommended by the manufacturer.

### Immunoblotting

Cells were lysed in a buffer containing 10 mM HEPES (pH 7.6), 1.5 mM MgCl_2_, 10 mM KCl, 0.1 mM EDTA, and complete protease and phosphatase inhibitors (Roche Diagnostics). For separating nuclear and cytoplasmic fractions, cells were lysed in a buffer containing 10 mM HEPES (pH 7.6), 1.5 mM MgCl_2_, 10 mM KCl, 0.1 mM EDTA, and complete proteinase and phosphatase inhibitors. Lysates were incubated for 15 min on ice. NP‐40 (10%) was added to the lysates (1/16 volume) and lysates were homogenized and centrifuged. Supernatants were collected as the cytosolic fraction. Next, the pellet was resuspended in buffer containing 20 mM HEPES (pH 7.6), 25% glycerol, 420 mM NaCl, 1.5 mM MgCl_2_, 0.2 mM EDTA, and complete proteinase and phosphatase inhibitors. Lysates were incubated on ice for 30 min and centrifuged for 5 min. Supernatants were collected as the nuclear fraction. Lysates were separated on 12% polyacrylamide gels and subjected to immunoblot analysis. Immunoblots were incubated overnight at 4°C with primary antibodies against TG‐interacting factor 1 (Tgif1) (1:1,000, Rabbit monoclonal, Abcam), Actin (1:5,000, Mouse monoclonal, Millipore), CREB‐regulated transcription coactivator 2 (Crtc2) (1:2,000, Rabbit polyclonal, Millipore) and Lamin A/C (1:500, Mouse monoclonal, Santa Cruz). Peroxidase‐labeled anti‐rabbit or anti‐mouse secondary antibodies (1:10,000, Santa Cruz) were used to visualize bands using the Clarity Western ECL Substrate (BioRad). Immunoblot images were acquired using the ChemiDoc imaging system and Image Lab software (BioRad). Quantification of band intensities was performed by gray scale analysis using Image Lab Software (Biorad). The intensity of the signal of interest was normalized to the intensity of the internal control signal (Actin or Lamin A/C). Normalized ratios are quantified and expressed as fold change relative to the experimental control group.

### DNA constructs and Luciferase assays

For the 3′UTR assays, mouse full‐length Tgif1 3′UTR was cloned into a pMIR‐Reporter Luciferase Plasmid (Applied Biosystems) downstream of the luciferase open reading frame to obtain the Tgif1‐3′UTR‐WT reporter plasmid. To generate a mutant reporter with a miss‐matched miR‐19a/b binding site (Tgif1‐3′UTR‐mut), the miR‐19a/b target sequence was altered using the QuickChange II XL site‐directed mutagenesis kit (Stratagene). MC3T3‐E1 cells were co‐transfected with the Luc‐Tgif1‐3′UTR reporter plasmid, the Renilla luciferase plasmid (Promega) as an internal transfection control, and miRNA mimic or inhibitor oligonucleotides (Ambion) by nucleoporation (NEON, Invitrogen) according to the manufacturer's instructions. Luciferase assays were performed using the Dual‐Luciferase Reporter Gene Assay System (Promega) according to instructions provided by the manufacturer. Firefly luciferase activity was normalized to Renilla luciferase activity.

### CTX ELISA

To analyze the serum concentration of carboxy‐terminal collagen crosslinks (CTX), serum was obtained by centrifuging peripheral blood at room temperature for 5 min. Samples were stored at −80°C until CTX ELISA (Immunodiagnostic Systems) was performed using 20 μl serum according to the manufacturer's protocol.

### *Ex vivo* osteoclast differentiation assay

Bone marrow cells were isolated from long bones of healthy, sham‐operated, or ovariectomized C57Bl/6J female mice and incubated in 10 cm dishes containing complete medium (α‐MEM supplemented with 10% FBS, 1% P/S) at 37°C for 3 h. Nonadherent cells were collected and further differentiated into osteoclast precursor cells by stimulating with macrophage colony‐stimulating factor (M‐CSF, 100 ng/ml) for 3–4 days. Osteoclast differentiation was induced by treating these cells with receptor activators of NF‐κB ligand (RANKL, 100 ng/ml) and M‐CSF (25 ng/ml). After 4 days of differentiation, cells were fixed with formalin and stained for tartrate‐resistant acid phosphatase (TRAP). Osteoclast differentiation was quantified by counting the number of TRAP‐positive cells with more than three nuclei using the Osteomeasure system (Osteometrics). To investigate the effect of anti‐miR‐19a/b on osteoclast differentiation, osteoclast precursor cells were transfected with scrambled control oligonucleotides (scr) or anti‐miR19a/b at a final concentration of 40 nM using Lipofectamine 3000. Osteoclast differentiation was induced by RANKL (100 ng/ml) and M‐CSF (25 ng/ml) treatment for 24 h after transfection, and osteoclast differentiation was determined after 4 days as described. For osteoclast differentiation assays using Ocy454‐derived CM, osteoclast precursor cells were stimulated with 30% CM supplemented with 50 ng/ml RANKL and 25 ng/ml M‐CSF. The medium was refreshed after 2 days, and cells were fixed and stained for TRAP after 4 days.

### Expression analysis of osteoblast‐derived paracrine factors

Ocy454 cells were plated and transfected with scrambled control oligonucleotides (scr) or anti‐miR19a/b 24 h prior to treatment with vehicle or PTH. After 4 h of treatment with vehicle or PTH, cells were harvested and RNA was isolated to quantify the mRNA expression of osteoblast‐derived paracrine factors known to stimulate osteoclast differentiation (components of Ephrin signaling: Eph2B, Eph4B, Ephrin2B; interleukins: IL6, IL11, IL18; insulin‐like growth factors: Igf1, Igf2; insulin‐like growth factor binding proteins: Igfbp2, Igfbp3, Igfbp5 and Rankl, Table EV6) using quantitative PCR. Data are normalized to the expression of the housekeeping gene Gapdh and expressed as relative expression compared with the scr and vehicle‐treated group.

### RANKL ELISA

To analyze the concentration of secreted RANKL, CM was collected from Ocy454 cells transfected with scr or anti‐miR‐19a/b and treated with vehicle or PTH as described. RANKL concentration was determined using an enzyme‐linked immunosorbent assay (ELISA) against RANKL (Thermo Scientific) according to the manufacturer's protocol.

### *In vitro* sex steroid‐deficiency models

Ocy454 cells were cultured in 12‐well plates in a complete medium for 24 h prior to stimulation with vehicle (0.1% Dimethyl sulfoxide, Sigma), 17‐β‐estradiol (100 nM, Sigma), or 5a‐dihydrotestosterone sulfate (20 nM, Sigma). After 24 h of stimulation, sex steroids were removed by washing the cells with 1× PBS and replacing the medium with phenol red‐free α‐MEM containing 10% FBS and 1% P/S. Four hours after the removal of the sex steroids, cells were harvested and RNA was isolated.

### Human bone samples

Human bone samples were obtained from the femoral heads of postmenopausal female patients of a comparable age (78.2 ± 3.9 years) undergoing elective hip replacement due to osteoarthritis at Helios ENDO‐Clinic Hamburg. Medical history with particular emphasis on conditions affecting bone metabolism including previous and current medication was obtained. In addition, comprehensive laboratory tests (e.g., differential blood count, hemoglobin, erythrocytes and derived parameters, thrombocytes, coagulation system including aPTT and INR, liver transaminases, electrolytes, C‐reactive protein, creatinine, urea, phosphorus, alkaline phosphatase (AP), bone‐specific‐AP, serum electrophoresis, Vitamin D, PTH, bone turnover markers P1NP and CTX, thyroid hormones, urine analysis) were performed. Only patients without major alterations of bone metabolism and in the absence of diseases or medications known to affect bone metabolism were included in this study. Furthermore, patients with a history of breast or prostate cancer or hip dysplasia were excluded as well. All parameters were chosen according to the national guideline for the diagnosis and therapy of postmenopausal osteoporosis. Immediately after surgery, femoral heads were cut into slices. One slice was rapidly snap frozen in liquid nitrogen and stored at −80°C for gene expression analyses. Another slice of the femoral head was used for μCT analyses and histology. A cylindrical tissue sample was harvested from an area that did not have cystic or sclerotic alterations of the trabecular bone. Samples were fixed in 3.7% formaldehyde for several days and stored in 70% ethanol until analysis by μCT (70 kV at 114 μA, 200 ms integration time; Viva80 micro‐CT; Scanco Medical AG). The threshold value was determined at 326 mg/cm^3^ hydroxyapatite based on Hounsfield units and a phantom with a linear hydroxyapatite gradient (79–729 mg/cm^3^). All analyses were performed on digitally extracted bone tissue using 3D distance techniques (Scanco Medical AG). Samples from eight patients were divided into a group with a relatively higher bone mass (BV/TV) (HBM, four patients) and a relatively lower bone mass (LBM, four patients). Bones were embedded in methylmetacrylate, cut into 4 μm sagittal sections, and stained with von Kossa/van Gieson. Bone histomorphometric measurements were performed using an OsteoMeasure system (OsteoMetrics). Total RNA was isolated from snap frozen samples and used for cDNA synthesis and miRNA expression analysis as described. Informed consent was obtained from all human subjects and, in addition to the principles set out in the WMA Declaration of Helsinki, the experiments conformed to the Department of Health and Human Services Belmont Report. The study protocol was approved by the local ethics committee.

### Statistical analyses

Data are presented as mean values ± SEM. Comparisons of two groups were made by using a two‐tailed Student's *t*‐test. One‐way analysis of variance (ANOVA), followed by Tukey's *post hoc* analysis was used to compare more than two groups unless otherwise stated. The nonparametric Kruskal–Wallis test was used to compare more than two groups in the dataset presented in Fig 3D and F, in which normal distribution could not be assumed. Statistical tests are indicated in the figure legends. Probability values were considered statistically significant at *P* < 0.05. Experiments were replicated in most cases at least three times and in few cases at least twice as biological replicates with a minimum of two technical replicates. Sample size was estimated based on standards used in the field. To minimize the effects of subjective bias, animals of the same gender and genotype were randomly assigned to treatment groups and samples were analyzed in a blinded manner.

## Author contributions

**Hanna Taipaleenmäki:** Conceptualization; resources; data curation; software; formal analysis; supervision; funding acquisition; validation; investigation; visualization; methodology; writing – original draft; project administration; writing – review and editing. **Hiroaki Saito:** Conceptualization; resources; data curation; software; formal analysis; validation; investigation; visualization; methodology. **Saskia Schröder:** Data curation; formal analysis; methodology. **Miki Maeda:** Investigation; methodology. **Ramona Mettler:** Data curation; investigation; methodology. **Matthias Ring:** Investigation; methodology. **Ewa Rollmann:** Data curation; investigation; visualization; methodology. **Andreas Gasser:** Conceptualization; data curation; formal analysis; validation; investigation; visualization; methodology. **Carl Haasper:** Data curation; supervision; investigation; methodology. **Thorsten Gehrke:** Conceptualization; supervision. **Alexander Weiss:** Data curation; formal analysis; investigation; methodology. **Steffen K Grimm:** Formal analysis; investigation; methodology. **Eric Hesse:** Conceptualization; resources; data curation; formal analysis; supervision; funding acquisition; validation; investigation; methodology; writing – original draft; project administration; writing – review and editing.

In addition to the CRediT author contributions listed above, the contributions in detail are:

HT conceived the project, designed and performed the experiments, analyzed the data, interpreted the results, and wrote the manuscript. HS performed the experiments, analyzed the data, and interpreted the results. SS performed the experiments, analyzed the data, and interpreted the results. MM and RM. ER designed and supervised the experiments, analyzed the data, interpreted the results, and contributed to the writing of the manuscript. AG designed the experiments, analyzed the data, and interpreted the results. MR collected the samples, performed the experiments, and analyzed the data. TG and CH contributed to the sample collection and interpreted the results. AW designed and supervised the experiments, analyzed the data, and interpreted the results. SKG designed the experiments, analyzed the data and interpreted the results. EH conceived the project, designed the experiments, analyzed the data, interpreted the results, and wrote the manuscript.

## Disclosure and competing interests statement

HT, HS, and EH are inventors of a patent (WO 2018/185270 A1) that is related to the topic. HT and EH are consultants of Sirana Pharma GmbH, which is using this patent. ER, AW, and SKG are employees of Evotec SE. All other authors have no conflict of interest.

## For more information

- American Society for Bone and Mineral Research (ASBMR): www.asbmr.org
- European Calcified Tissue Society (ECTS): www.ectsoc.org
- International Osteoporosis Foundation (IOF): www.osteoporosis.foundation
- Bone Health & Osteoporosis Foundation (BHOF): www.bonehealthandosteoporosis.org

## Supporting information




Appendix S1

Expanded View Figures PDF

Table EV1

Table EV2

Table EV3

Table EV4

Table EV5

Table EV6

Source Data for Expanded View

PDF+

Source Data for Figure 2

Source Data for Figure 3

Source Data for Figure 4

Source Data for Figure 6

## Data Availability

The miRNA sequencing datasets produced in this study are available in the following database: https://www.ncbi.nlm.nih.gov/bioproject/?term=PRJNA836630 with the BioProject number PRJNA836630.
