# Supplementary material for: Antagonizing microRNA‐19a/b augments PTH anabolic action and restores bone mass in osteoporosis in mice
Source: EMBO Mol Med. 2022 Oct 4;14(11):e13617. doi: 10.15252/emmm.202013617 (PMC9641424; doi:10.15252/emmm.202013617)
Supplement: Supplementary file 1 — Appendix S1 [file EMMM-14-e13617-s004.pdf]

Appendix

Table of content

Appendix Figure S1
Appendix Figure S2
Appendix Figure S3
Appendix Figure S4
Appendix Figure S5
Appendix Figure S6

Appendix Figure S1

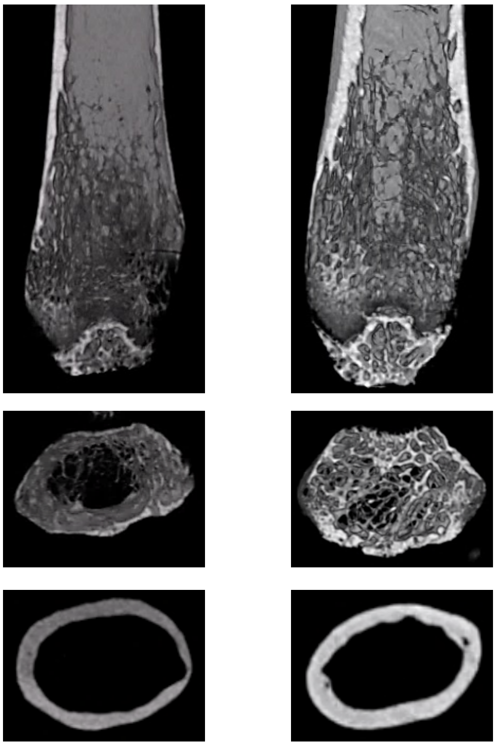

D

	Parameters	Male: scr	Male: anti-miR-19a/b
Distal femur	BV/TV (%)	16.53 ± 1.36 (n=8)	23.69 ± 2.67* (n=8)
	Tb.Th (μm)	42.61 ± 2.12 (n=8)	50.25 ± 3.08 (n=8)
	Tb.Sp (μm)	221.0 ± 11.3 (n=8)	166.0 ± 15.1* (n=8)
	Tb.N (1/mm)	3.832 ± 0.135 (n=8)	4.786 ± 0.261* (n=8)
	SMI	2.329 ± 0.106 (n=8)	1.646 ± 0.215* (n=8)
Midshaft femur	Ct.Th (μm)	168.8 ± 5.4 (n=8)	183.4 ± 4.2 (n=8)
	Ct.Dens (mg HA/cm^3)	941.9 ± 18.7 (n=8)	1015 ± 8.0** (n=8)
	Ps.Dm (mm)	1.877 ± 0.029 (n=8)	1.966 ± 0.030 (n=8)
	Ps.Pm (mm)	5.895 ± 0.092 (n=8)	6.175 ± 0.093 (n=8)
	Ec.Dm (mm)	1.616 ± 0.029 (n=8)	1.681 ± 0.026 (n=8)
	Ec.Pm (mm)	5.075 ± 0.092 (n=8)	5.279 ± 0.083 (n=8)

μCT analysis in 12-week old mice. Mean values ± SEM. * $p<0.05$, ** $p<0.01$.

Appendix Figure S1 Antagonizing miR-19a/b increases bone mass. **A**, **B** μCT of the distal- and **C** midshaft (cross sections) femora of mice treated with scrambled (scr, n=8) or anti-miR-19a/b (n=8). **D** Values of the μCT analysis of the same animals as in **A**-**C**. Data information: Student's t-test was used for statistical analysis. *$p<0.05$, **$p<0.01$, vs. scr.

Appendix Figure S2

miR-19a/b 3’…ACCU**AAACGUGU**

Mouse 5’…AAUAUUUGCACAUGGGAUUGCU…
Human …AAUAUUUGCACAUGGGAUUGCU…
Chimpanzee…AAUAUUUGCACAUGGGAUUGCU…
Rat …AAUAUUUGCACAUGGGAUUGCU…
Rabbit …AAUAUUUGCACAUGGGAUUGCU…
Hedgehog …AAUAUUUGCACAUGGGAUUGCU…
Dog …AAUAUUUGCACAUGGGAUUGCU…
Cat …AAUAUUUGCACAUGGGAUUGCU…
Horse …AAUAUUUGCACAUGGGAUUGCU…
Cow …AAUAUUUGCACAUGGGAUUGCU…
Elephant …AAUAUUUGCACAUGGGAUUGCU…

Tgif1 Consensus …AAUAUUUGCACAUGGGAUUGCU…

Appendix Figure S2 Tgif1 is a conserved target of miR-19a/b. *In silico* analysis of the putative consensus binding site for miR-19a and miR-19b (red letters) at the Tgif1 3’ untranslated region (UTR) in various species as indicated.

Appendix Figure S3

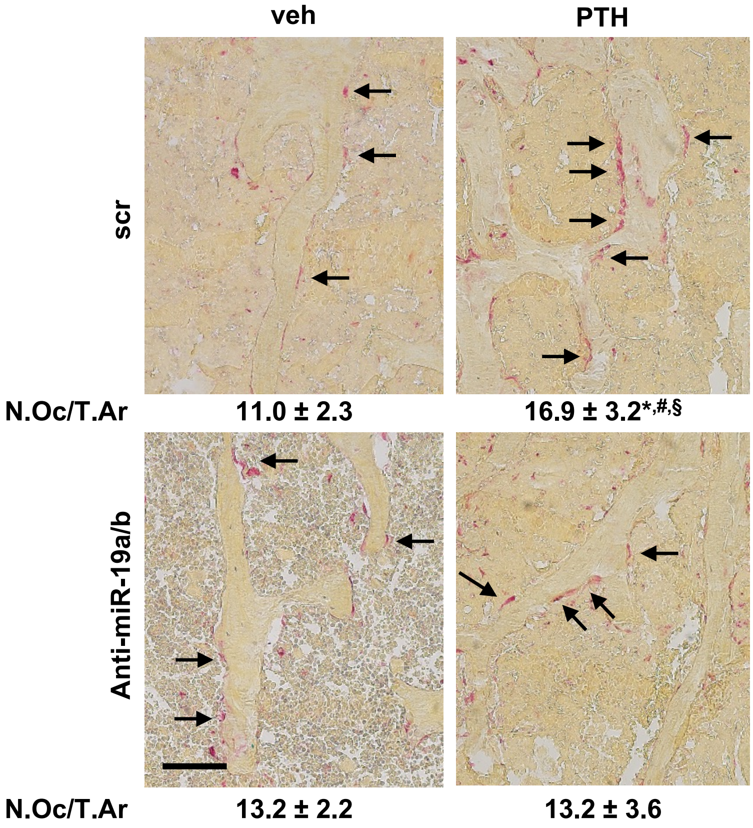

Appendix Figure S3 Anti-miR-19a/b treatment prevents the PTH-induced increase in osteoclast number. Representative images of TRAP-positive osteoclasts (black arrows) in the vertebrae of 12-week-old male mice after treatment with intermittent PTH or vehicle (veh) and/or weekly injections of anti-miR-19a/b or scrambled (scr) control for 4 weeks. Scr; veh n=8, anti-miR-19a/b; veh n=10, scr; PTH n=12, anti-miR-19a/b; PTH n=10. The number of osteoclasts (N.Oc/T.Ar) is indicated. Scale bar indicates 50µm (black). Data information: Mean values ± SEM. One-way ANOVA followed by Tukey's post-hoc analysis was used for statistical analysis. *p<0.002 vs. veh; scr, #<0.05 vs. veh; anti-miR-19a/b, §p<0.05 vs. PTH; anti-miR-19a/b.

Appendix Figure S4

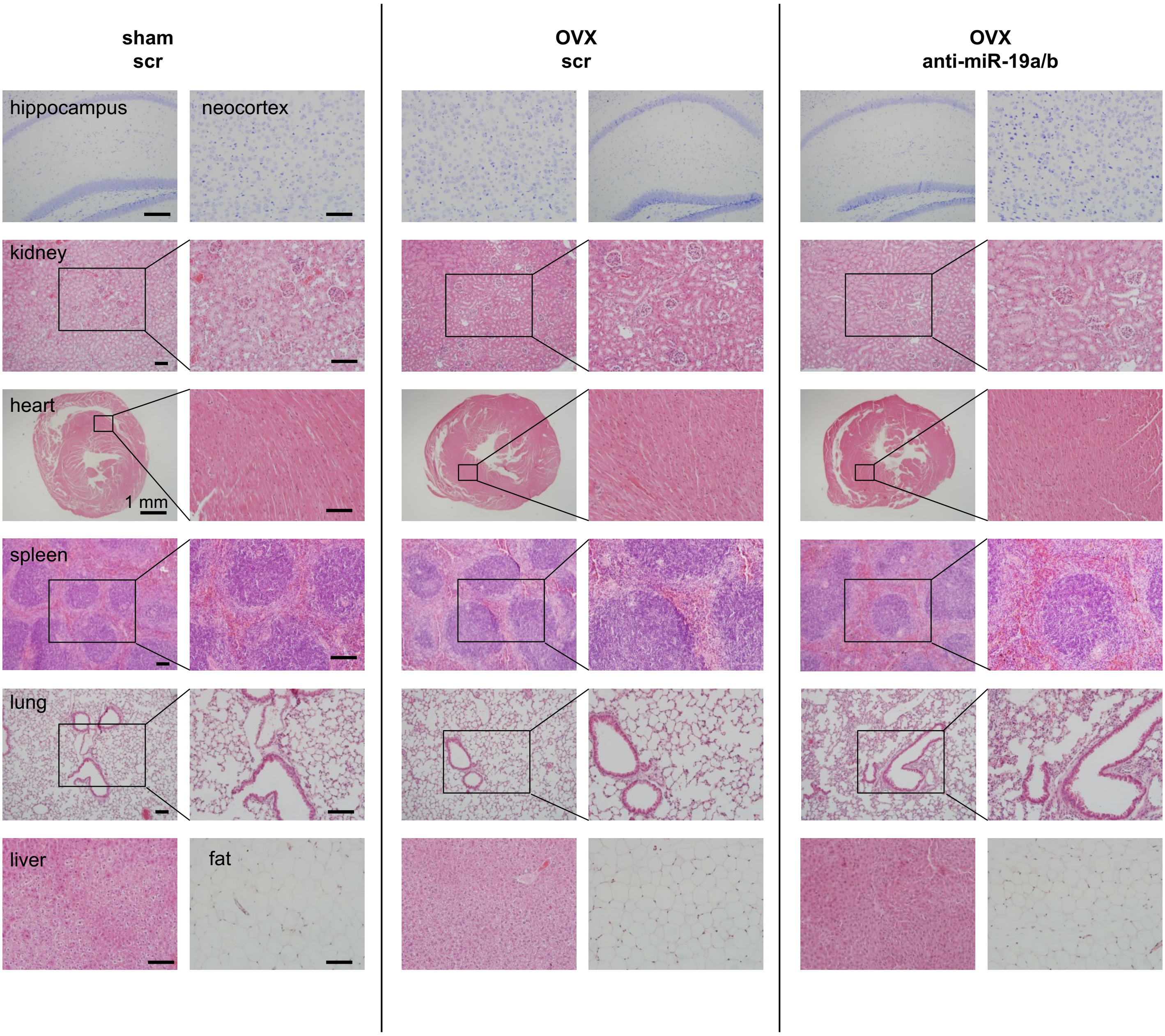

Appendix Figure S4 Histological analysis does not show overt adverse effects of anti-miR-19a/b treatment on tissue morphology in ovariectomized mice. Histology of various organs of female mice after sham operation or ovariectomy (OVX) and seven weeks treatment with scrambled control oligonucleotides (sham; scr, n=8; OVX; scr, n=7) or anti-miR-19a/b (OVX; anti-miR-19a/b, n=7). Organs were stained by Nissl stain (brain sections), with Periodic acid-Schiff (PAS) (kidney sections) or with haematoxylin and eosin (all other organs). Scale bars indicate 100 µm unless otherwise noted. Representative images are shown.

Appendix Figure S5

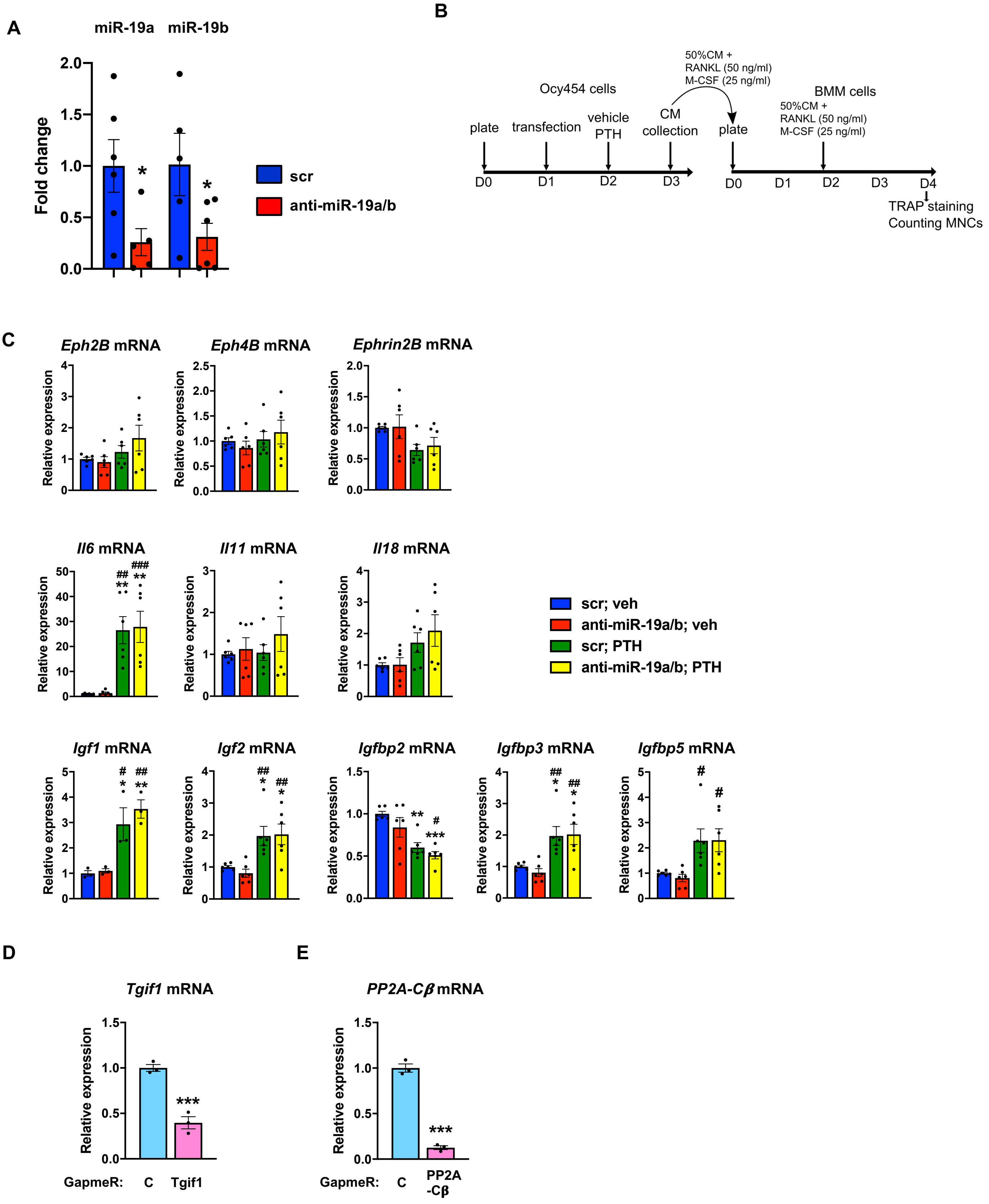

Appendix Figure S5 A miR-19a and miR-19b expression in osteoclast precursors cells transfected with scrambled control oligonucleotides (scr) or anti-miR-19a/b, n=5. **B** Schematic illustrating the experimental setup of the osteoclast differentiation assay in the presence of condition medium (CM). **C** Quantification of the expression of factors in Ocy454 cells that are related to osteoblast-osteoclast interaction, n=6. **D** Expression of *Tgif1* mRNA in Ocy454 cells after transfection with a scrambled control GapmeR (GapmeR C) or GapmeR against Tgif1, n=3. **E** Expression of *PP2A-Cβ* mRNA in Ocy454 cells after transfection with a scrambled control GapmeR (C) or GapmeR against PP2A-Cβ. Data information: Mean values ± SEM. Student's t-test was used to compare two groups (A, D). For comparison of more than two groups One-way ANOVA followed by Tukey's post-hoc analysis was used (C). *p<0.05, **p<0.01, ***p<0.001 vs. scr (**A**), vs. scr; veh (**C**) vs. GapmeR C (**D**), $^{\#}$p<0.05, $^{\#\#}$p<0.01, $^{\#\#}$p<0.001 vs. anti-miR-19a/b; veh (**C**).

Appendix Figure S6

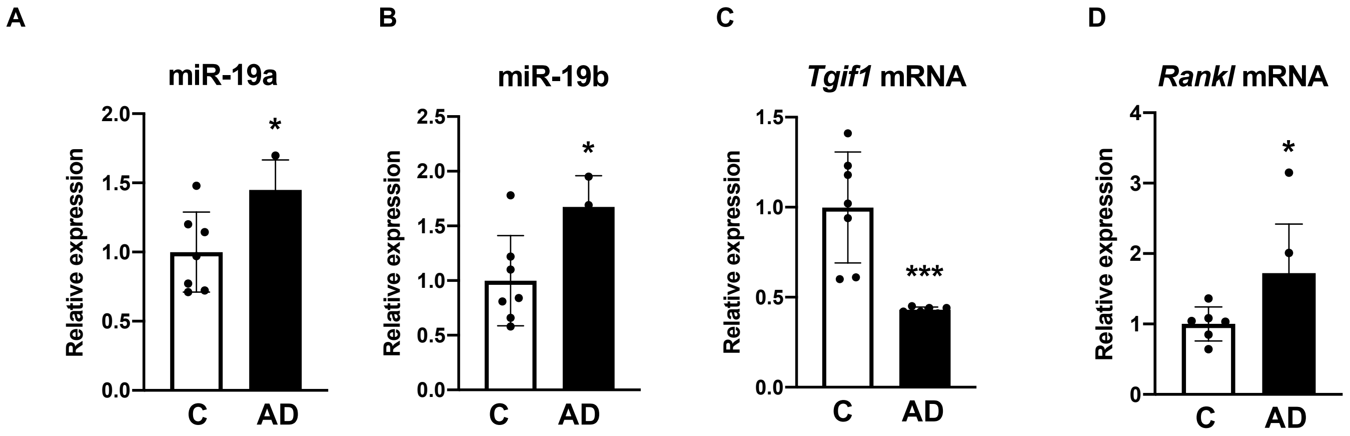

Appendix Figure S6 A Relative expression of miR-19a, **B** miR-19b, **C** Rankl and **D** Tgif1 mRNA in Ocy454 cells upon androgen deficiency (AD) and control (C) treatment, n=6. Data information: Mean values ± SEM. Student's t-test was used for statistical analysis. $^{*}p<0.05$, $^{***}p<0.001$ vs. C.
