# Supplementary material for: Antagonizing microRNA‐19a/b augments PTH anabolic action and restores bone mass in osteoporosis in mice
Source: EMBO Mol Med. 2022 Oct 4;14(11):e13617. doi: 10.15252/emmm.202013617 (PMC9641424; doi:10.15252/emmm.202013617)
Supplement: Supplementary file 2 — Expanded View Figures PDF [file EMMM-14-e13617-s007.pdf]

Expanded View Figures

Figure EV1. MiR-19a and miR-19b are expressed in various organs and downregulated by anti-miR-19a/b treatment.

A Absolute expression (number of counts) of the 22 miRNAs in vehicle-treated samples (n = 4) whose expression is decreased in mouse tibiae by PTH and Scl-Ab treatment.

B, C Expression of miR-19a (B) and miR-19b (C) in MC3T3-E1 cells after transfection with scrambled (scr) control oligonucleotide, anti-miR-19a, anti-miR-19b, or anti-miR-19a/b (n = 3).

D, E Expression of miR-19a (D) and miR-19b (E) in mouse organs and tissues (n = 6). Values are normalized to U6 as an internal control (ΔC_T).

F, G Relative expression of miR-19a (F) and miR-19b (G) in mouse organs and tissues after 4-weeks of intravenous (i.v.) treatment with vehicle (veh, n = 3), scrambled (scr, n = 4) or anti-miR-19a/b (n = 4).

Data information: Mean values ± SEM. One-way ANOVA followed by Tukey's *post hoc* analysis was used to compare the groups. *P < 0.05 vs. veh. (F, G), ***P < 0.001 vs. scr (B, C).

© 2022 The Authors

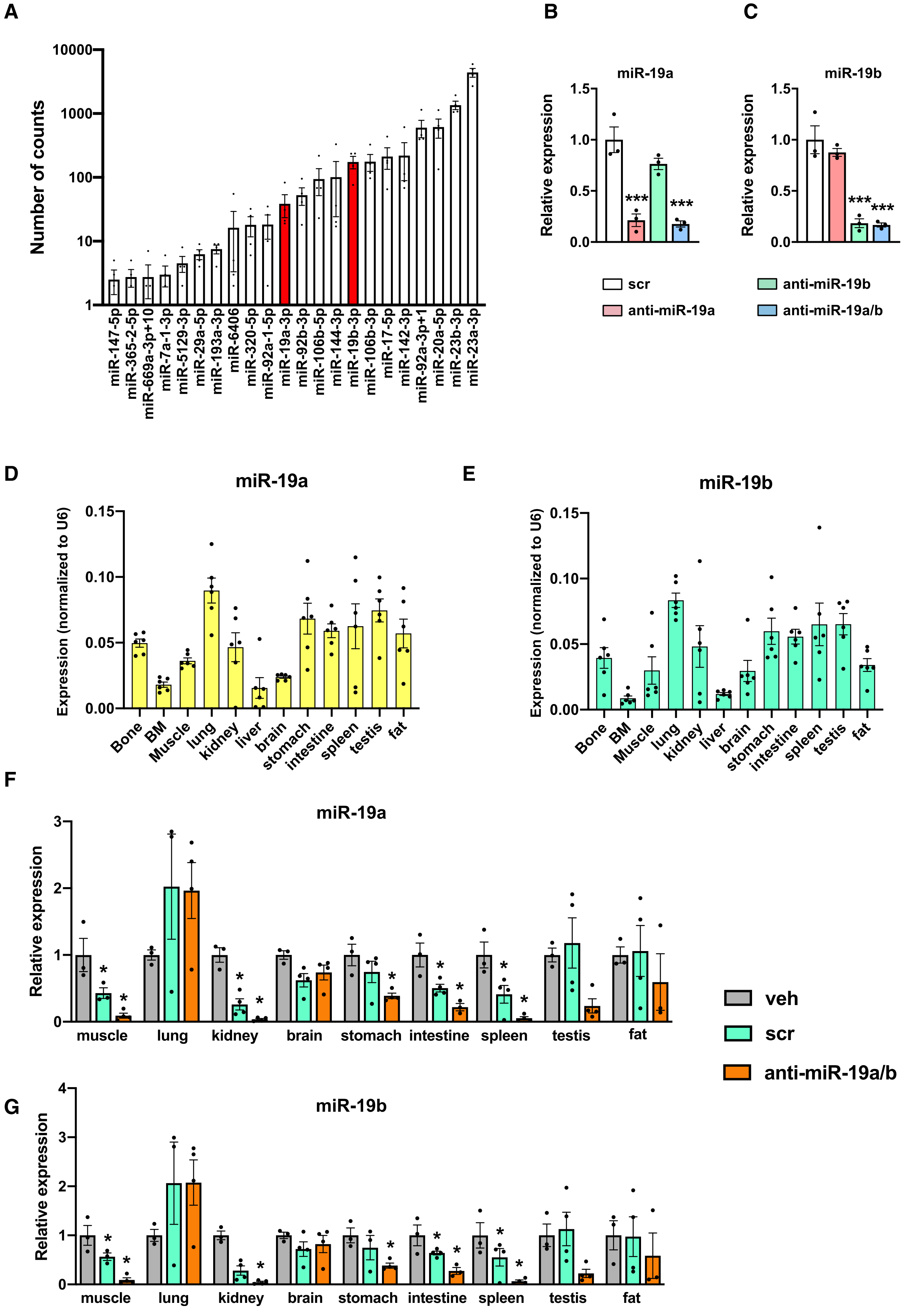
A
Number of counts
10000
1000
100
10
1
miR-147-5p
miR-365-2-5p
miR-669a-3p+10
miR-7a-1-3p
miR-5129-3p
miR-29a-5p
miR-193a-3p
miR-6406
miR-320-5p
miR-92a-1-5p
miR-19a-3p
miR-92b-3p
miR-106b-5p
miR-144-3p
miR-19b-3p
miR-106b-3p
miR-17-5p
miR-142-3p
miR-92a-3p+1
miR-20a-5p
miR-23b-3p
miR-23a-3p
B
miR-19a
Relative expression
***
***
C
miR-19b
Relative expression
***
***
scr
anti-miR-19a
anti-miR-19b
anti-miR-19a/b
D
miR-19a
Expression (normalized to U6)
Bone
BM
Muscle
lung
kidney
liver
brain
stomach
intestine
spleen
testis
fat
E
miR-19b
Expression (normalized to U6)
F
miR-19a
Relative expression
muscle
lung
kidney
brain
stomach
intestine
spleen
testis
fat
veh
scr
anti-miR-19a/b
G
miR-19b
Relative expression

Figure EV1.

Figure EV2. Histological analysis does not reveal overt adverse effects of anti-miR-19a/b treatment on tissue morphology.

A Histology of various mouse organs as indicated after 4 weeks of treatment with vehicle (veh, $n = 8$), scrambled control oligonucleotide (scr, $n = 11$) or anti-miR-19a/b ($n = 11$). Organs were stained by Nissl stain (brain sections), with periodic acid–Schiff (PAS) (kidney sections), or with hematoxylin and eosin (all other tissues). Scale bars indicate 100 μm unless otherwise noted. Representative images are shown.

B Body weight of mice after 4 weeks of treatment with veh ($n = 8$), scr ($n = 8$) or anti-miR-19a/b ($n = 8$).

Data information: Mean values ± SEM. One-way ANOVA followed by Tukey's *post hoc* analysis was used to compare three groups.

 © 2022 The Authors

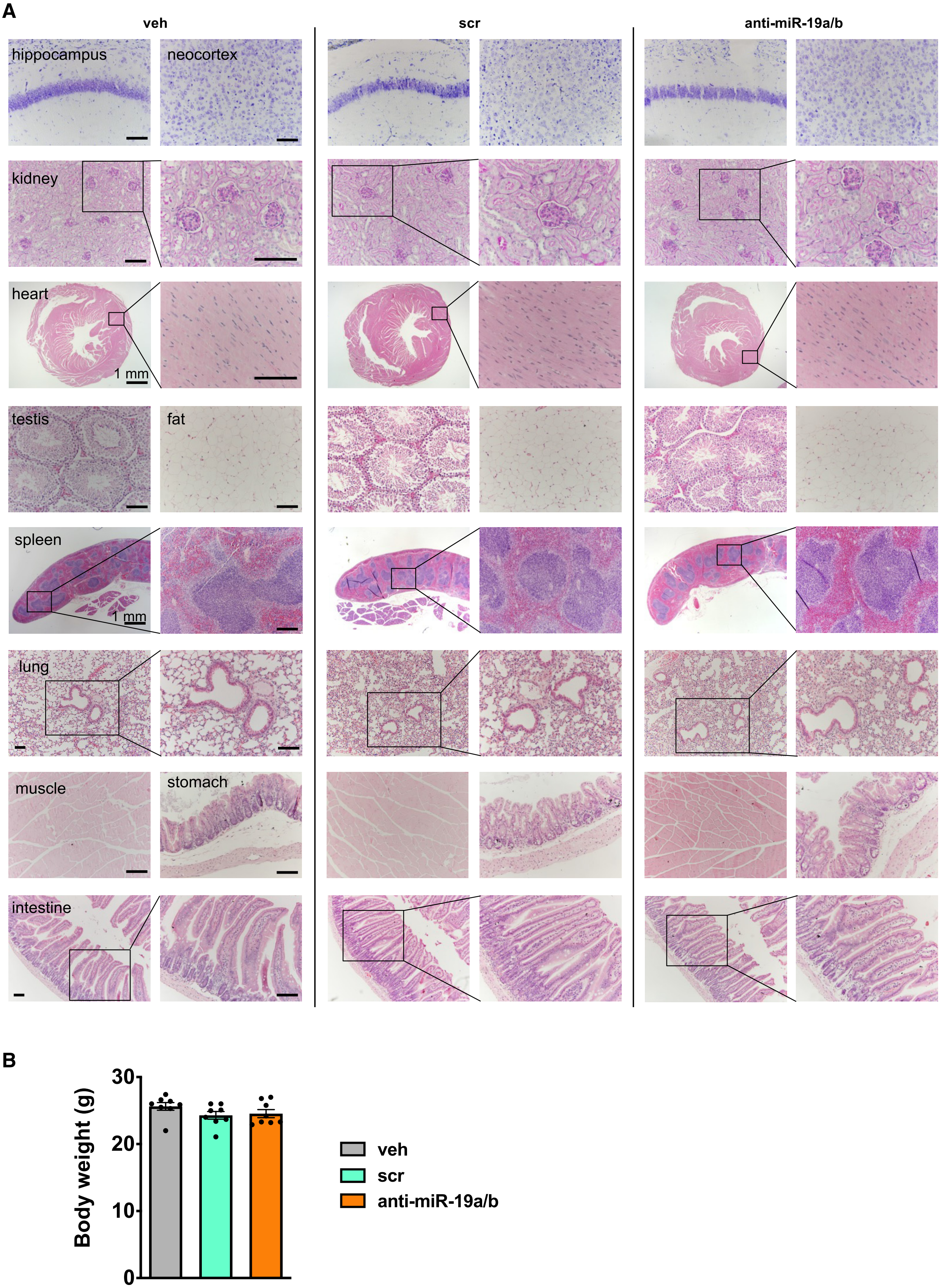
A
veh
scr
anti-miR-19a/b
hippocampus
neocortex
kidney
heart
1 mm
testis
fat
spleen
1 mm
lung
muscle
stomach
intestine
B
Body weight (g)
30
20
10
0
veh
scr
anti-miR-19a/b

Figure EV2.

Figure EV3. Gene expression and functional analysis do not indicate cytotoxic effects.

A Quantification of the expression of the housekeeping genes glyceraldehyde-3-phosphate dehydrogenase (Gapdh), beta-2 microglobulin (b2m), beta-actin 1 (β-actin 1) and beta-actin 2 (β-actin 2) in several organs upon treatment with anti-miR-19a/b ($n = 4$), scrambled control oligonucleotide (scr, $n = 4$) or vehicle (veh, $n = 3$).

B Growth of osteoblasts of the MC3T3-E1 cell line ($n = 9$) and of hepatocytes of the Hep2G cell line ($n = 6$) 24 h after treatment with anti-miR-19a/b, scrambled control oligonucleotide (scr) or vehicle (veh).

C–D (C) Quantification of the expression of endogenous miR-19a and miR-19b in cells of the MC3T3-E1 ($n = 8$) and (D) HepG2 ($n = 6$) cell lines after treatment with anti-miR-19a/b, scr or veh. Data information: Mean values ± SEM. Three groups were compared using one-way ANOVA followed by Tukey's *post hoc* analysis. $^{***}P < 0.001$ vs. veh. $^{\#\#\#}P < 0.001$ vs. scr.

Source data are available online for this figure.

 © 2022 The Authors

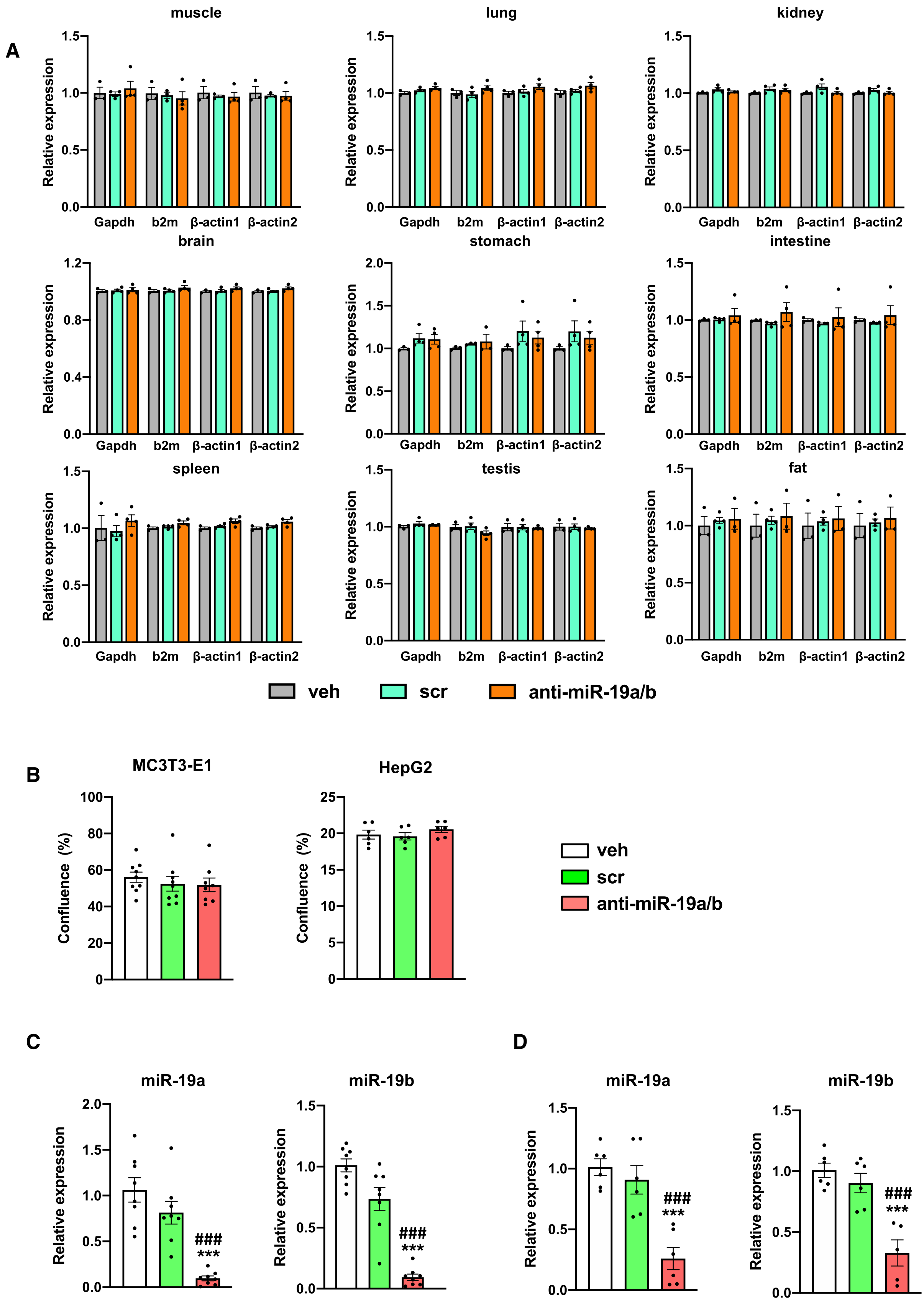
A
muscle
lung
kidney
brain
stomach
intestine
spleen
testis
fat
Relative expression
Gapdh
b2m
β-actin1
β-actin2
veh
scr
anti-miR-19a/b
B
MC3T3-E1
HepG2
Confluence (%)
veh
scr
anti-miR-19a/b
C
D
miR-19a
miR-19b
Relative expression
###
***

Figure EV3.

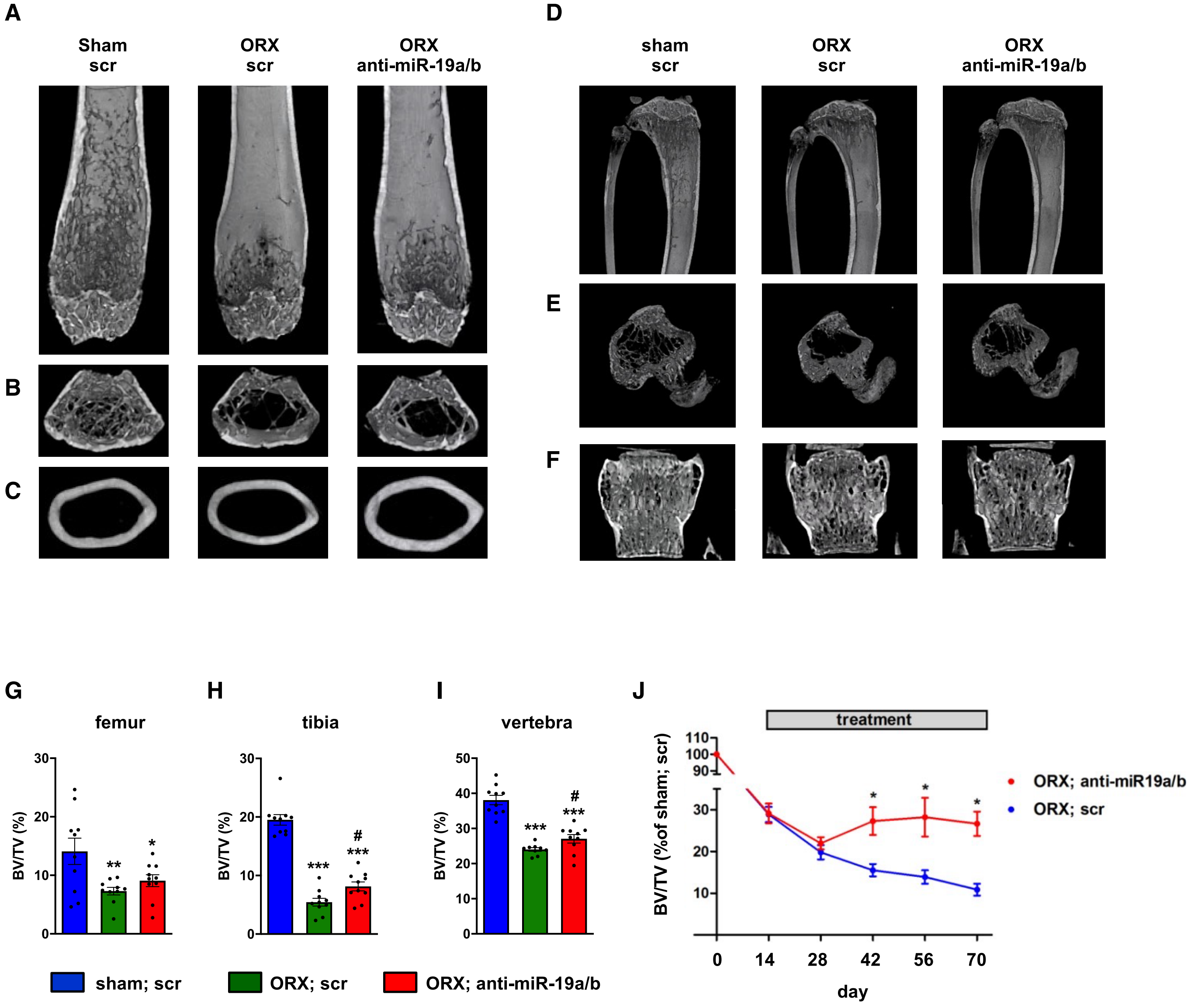

Figure EV4. Anti-miR-19a/b treatment alleviates bone loss induced by orchiectomy.

A–C (A, B) µCT scans of the distal femora and (C) of midshaft femoral cross sections of sham-operated male mice treated with scrambled control oligonucleotides (scr, *n* = 10) and of male mice in which osteoporosis was induced by orchiectomy (ORX) 14 days before the start of weekly treatment with anti-miR-19a/b (*n* = 10) or scr (*n* = 10).

D–F (D, E) µCT scans of the proximal tibiae and (F) the fourth lumbar vertebral bodies of the same mice as in (A–C).

G–I (G) µCT-based quantification of the trabecular bone mass (BV/TV, bone volume/tissue volume) in distal femora, (H) proximal tibiae, and (I) fourth lumbar vertebral bodies 70 days after ORX, sham, scr (*n* = 10); ORX, scr (*n* = 10); ORX, anti-miR-19a/b (*n* = 10).

J Time course of the relative change (compared with sham-operated and scr-treated control) in BV/TV in the tibiae of male mice in which osteoporosis was induced by ORX 14 days before the start of weekly treatment with scr or anti-miR-19a/b, sham, scr (*n* = 10); ORX, scr (*n* = 10); ORX, anti-miR-19a/b (*n* = 10).

Data information: Mean values ± SEM. One-way ANOVA followed by Tukey's *post hoc* analysis was used for statistical analysis. *$P < 0.05$, **$P < 0.01$, ***$P < 0.001$ vs. sham; scr, #$P < 0.05$ vs. ORX; scr.

 © 2022 The Authors

Figure EV5. Anti-miR-19a/b treatment restores ovariectomy-induced bone loss in mice.

A–C (A, B) μCT scans of the proximal tibiae and (C) the fourth lumbar vertebral bodies of female mice in which osteoporosis was induced by ovariectomy (OVX) 21 days before the start of weekly treatment with scrambled control oligonucleotides (scr, $n = 6$) or anti-miR-19a/b ($n = 8$). Sham-operated and scr-treated animals served as controls ($n = 10$).

D Histomorphometric analysis of the cortical thickness at the midshaft femora of the same mice as in (A–C) after termination of the experiment.

E, F (E) Uterus weight and (F) body weight of female mice after sham operation ($n = 8$) or ovariectomy (OVX) and 7 weeks of treatment with scr ($n = 6$) or anti-miR-19a/b ($n = 7$).

G mRNA expression of inflammatory markers Ccl5, Ccl2, Interferon gamma (Inf-γ), Tumor necrosis factor alpha (Tnf-α), F4/80, Interleukin 1 (Il1), Interleukin 1b (Il1b), Interleukin 6 (Il6), cluster of differentiation 11b (Cd11b) and cluster of differentiation 11c (Cd11c) in tibiae of sham-operated scr-treated mice ($n = 11$) and OVX mice treated with scr ($n = 6$) or anti-miR-19a/b ($n = 7$).

Data information: Mean values ± SEM. One-way ANOVA followed by Tukey's *post hoc* analysis was used for statistical analysis. *$P < 0.05$, **$P < 0.01$, ***$P < 0.001$ vs. sham; scr, $^{###}P < 0.001$ vs. OVX; scr.

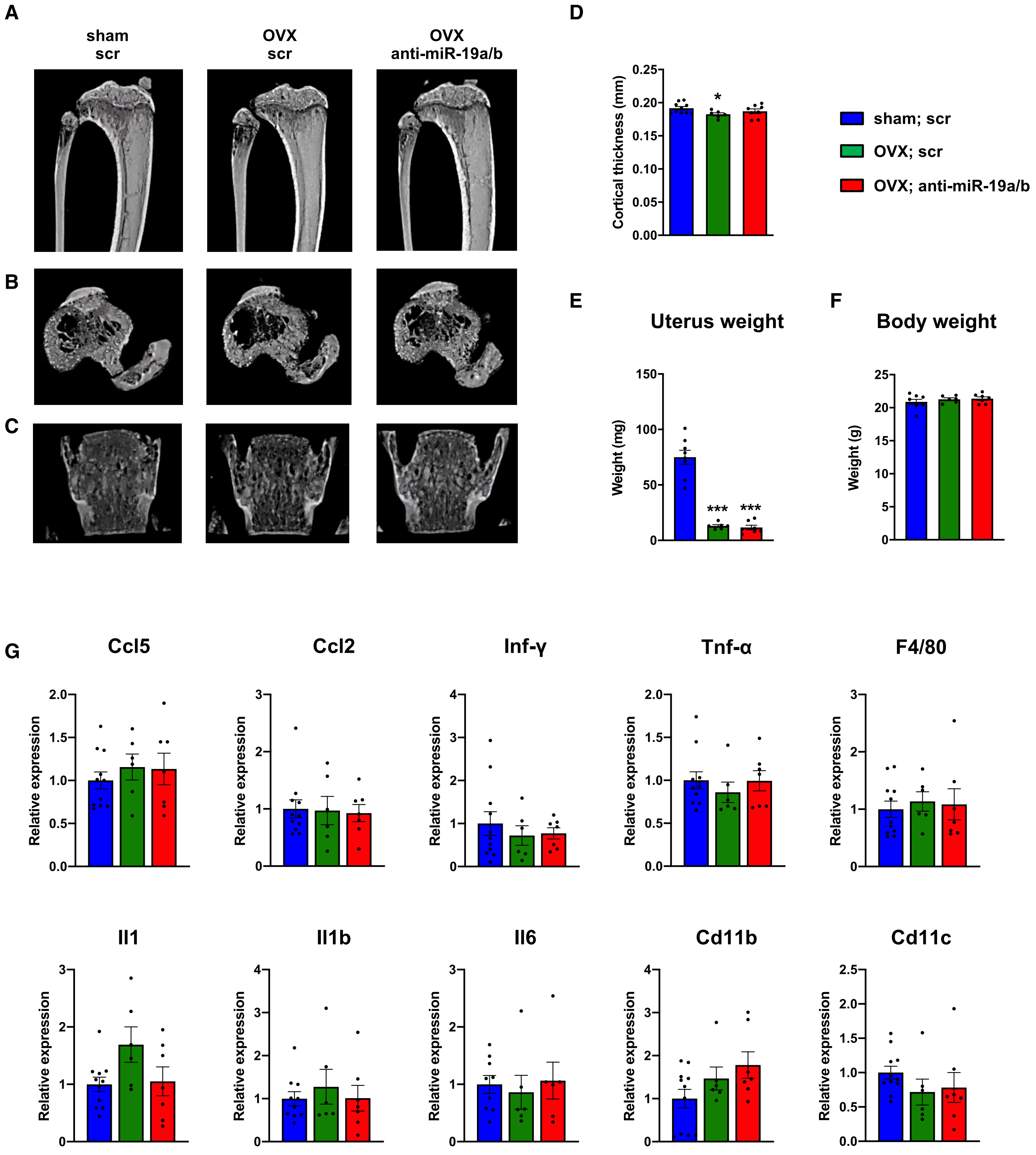

Figure EV5.

 © 2022 The Authors
