# Supplementary material for: Antagonizing microRNA‐19a/b augments PTH anabolic action and restores bone mass in osteoporosis in mice
Source: EMBO Mol Med. 2022 Oct 4;14(11):e13617. doi: 10.15252/emmm.202013617 (PMC9641424; doi:10.15252/emmm.202013617)
Supplement: Supplementary file 3 — Table EV1 [file EMMM-14-e13617-s005.pdf]

Table EV1. Histomorphometric analysis of the proximal tibiae and vertebrae in wild type mice after anti-miR-19a/b treatment

		Male		
	Parameters	vehicle	scr	anti-miR-19a/b
Proximal tibia	BV/TV (%)	6.09 ± 0.22 (n=8)	5.95 ± 0.59 (n=11)	7.72 ± 0.52$^{*\#}$ (n=11)
	Tb.Th (µm)	31.30 ± 1.18 (n=8)	27.93 ± 1.30 (n=11)	29.96 ± 1.02 (n=11)
	Tb.Sp (µm)	488.9 ± 29.5 (n=8)	481.7 ± 47.1 (n=11)	376.1 ± 29.6 (n=11)
	Tb.N (1/mm)	1.965 ± 0.107 (n=8)	2.105 ± 0.158 (n=11)	2.558 ± 0.136$^{*\#}$ (n=11)
	MS/BS (%)	28.06 ± 1.45 (n=8)	23.93 ± 1.41 (n=11)	34.80 ± 2.28$^{*\#\#\#}$ (n=12)
	MAR (µm/day)	1.248 ± 0.050 (n=8)	1.432 ± 0.066 (n=11)	1.837 ± 0.071$^{***\#\#\#}$ (n=12)
	BFR/BS ($\mu m^3/\mu m^2$/year)	128.6 ± 9.5 (n=8)	125.7 ± 10.4 (n=11)	231.4 ± 14.6$^{***\#\#\#}$ (n=12)
	BFR/BV (%/year)	583.8 ± 56.4 (n=8)	651.9 ± 66.3 (n=11)	1257 ± 104.7$^{***\#\#\#}$ (n=12)
	OV/BV (%)	1.534 ± 0.202 (n=8)	1.633 ± 0.171 (n=9)	3.673 ± 0.449$^{***\#\#\#}$ (n=9)
	OS/BS (%)	10.29 ± 0.91 (n=8)	11.23 ± 1.05 (n=9)	20.33 ± 1.12$^{***\#\#\#}$ (n=9)
	Ob.S/BS (%)	10.78 ± 0.91 (n=8)	11.96 ± 1.11 (n=9)	21.36 ± 1.29$^{***\#\#\#}$ (n=9)
	N.Ob/BS (1/mm)	6.531 ± 0.683 (n=8)	7.265 ± 0.673 (n=9)	13.06 ± 0.78$^{***\#\#\#}$ (n=9)
	ES/BS (%)	0.8111 ± 0.0916 (n=8)	0.6342 ± 0.1110 (n=9)	0.9969 ± 0.1212 (n=9)
	Oc.S/BS (%)	0.6792 ± 0.0803 (n=8)	0.5308 ± 0.0731 (n=9)	0.9231 ± 0.1282$^{\#}$ (n=9)
	N.Oc/BS (1/mm)	0.3172 ± 0.0370 (n=8)	0.2470 ± 0.0322 (n=9)	0.3820 ± 0.0484 (n=9)
Vertebral body	BV/TV (%)	13.50 ± 0.99 (n=8)	13.92 ± 0.76 (n=12)	16.08 ± 0.66$^{*\#}$ (n=12)
	Tb.Th (µm)	30.71 ± 1.10 (n=8)	29.34 ± 0.91 (n=12)	30.80 ± 1.09 (n=12)
	Tb.Sp (µm)	194.9 ± 14.56 (n=8)	162.6 ± 6.4* (n=12)	162.0 ± 5.1* (n=12)
	Tb.N (1/mm)	4.581 ± 0.289 (n=8)	5.263 ± 0.154* (n=12)	5.228 ± 0.137* (n=12)

Histomorphometry of the proximal tibiae and the L4 vertebral bodies of 12-week old mice. Mean values ± SEM. * p<0.05, ***p<0.001 vs. vehicle, $^{\#}$ p<0.05, $^{\#\#\#}$ p<0.001 vs. scr.
