# Supplementary material for: Antagonizing microRNA‐19a/b augments PTH anabolic action and restores bone mass in osteoporosis in mice
Source: EMBO Mol Med. 2022 Oct 4;14(11):e13617. doi: 10.15252/emmm.202013617 (PMC9641424; doi:10.15252/emmm.202013617)
Supplement: Supplementary file 4 — Table EV2 [file EMMM-14-e13617-s006.pdf]

Table EV2. Histomorphometric analysis of the proximal tibiae and vertebrae in Dmp1-Cre$^-$;Tgif1$^{fl/fl}$ and Dmp1-Cre^{Tg};Tgif1$^{fl/fl}$ male mice treated with anti-miR-19a/b

	Parameters	Male			
		Dmp1-Cre$^-$;Tgif1$^{fl/fl}$, scr	Dmp1-Cre$^-$;Tgif1$^{fl/fl}$, anti-miR-19a/b	Dmp1-Cre^{Tg};Tgif1$^{fl/fl}$, scr	Dmp1-Cre^{Tg};Tgif1$^{fl/fl}$, anti-miR-19a/b
Proximal tibia	BV/TV (%)	7.628 ± 0.581 (n=8)	11.38 ± 0.918* (n=10)	6.573 ± 0.368$^{\#\#}$ (n=7)	8.305 ± 0.576$^{\#}$ (n=14)
	Tb.Th (µm)	28.55 ± 1.08 (n=8)	34.30 ± 1.92* (n=10)	29.71 ± 1.604 (n=7)	30.19 ± 0.85 (n=14)
	Tb.Sp (µm)	355.0 ± 19.0 (n=8)	279.1 ± 20.9 (n=10)	430.5 ± 35.7$^{\#\#}$ (n=7)	348.3 ± 18.3 (n=14)
	Tb.N (1/mm)	2.651 ± 0.132 (n=8)	3.308 ± 0.202* (n=10)	2.246 ± 0.154 (n=7)	2.723 ± 0.137 (n=14)
	MS/BS (%)	26.32 ± 1.04 (n=7)	38.48 ± 0.88** (n=9)	21.13 ± 1.13$^{\#\#\#}$ (n=7)	30.61 ± 1.42$^{\#,§}$ (n=14)
	MAR (µm/day)	1.134 ± 0.056 (n=7)	2.033 ± 0.123*** (n=9)	0.8749 ± 0.0618$^{\#\#\#}$ (n=7)	1.563 ± 0.082§§ (n=14)
	BFR/BS (µm^3/µm^2/year)	108.2 ± 4.9 (n=7)	287.1 ± 21.8*** (n=9)	68.57 ± 8.10$^{\#\#\#}$ (n=7)	174.7 ± 11.7§§ (n=14)
	BFR/BV (%/year)	589.4 ± 46.2 (n=7)	1285 ± 97** (n=9)	388.7 ± 56.3$^{\#\#\#}$ (n=7)	855.0 ± 63.3§ (n=11)
	OV/BV (%)	1.960 ± 0.083 (n=8)	4.866 ± 0.409** (n=9)	1.064 ± 0.206$^{\#\#\#}$ (n=7)	2.313 ± 0.197$^{\#}$ (n=11)
	OS/BS (%)	12.71 ± 0.73 (n=8)	26.58 ± 1.50** (n=9)	7.619 ± 1.130$^{\#\#\#}$ (n=7)	14.82 ± 1.23$^{\#}$ (n=11)
	Ob.S/BS (%)	12.77 ± 0.83 (n=8)	25.98 ± 1.67* (n=9)	7.587 ± 1.046$^{\#\#\#}$ (n=7)	14.82 ± 1.18$^{\#}$ (n=11)
	N.Ob/BS (1/mm)	7.762 ± 0.560 (n=8)	16.71 ± 0.83** (n=9)	4.574 ± 0.555$^{\#\#\#}$ (n=7)	9.266 ± 0.654§ (n=11)
	ES/BS (%)	0.8232 ± 0.0684 (n=8)	1.042 ± 0.0884 (n=9)	0.3635 ± 0.0408*,$^{\#\#\#}$ (n=7)	0.835 ± 0.074§ (n=11)
	Oc.S/BS (%)	0.6121 ± 0.0479 (n=8)	0.9226 ± 0.1007 (n=9)	0.4219 ± 0.0439*,$^{\#\#\#}$ (n=7)	0.7104 ± 0.0727§ (n=11)
	N.Oc/BS (1/mm)	0.3128 ± 0.0260 (n=8)	0.3861 ± 0.0394 (n=9)	0.1630 ± 0.0191$^{\#\#\#}$ (n=7)	0.304 ± 0.023§ (n=11)
Vertebral body	BV/TV (%)	15.36 ± 0.45 (n=8)	18.72 ± 1.00* (n=10)	14.32 ± 0.49 (n=7)	15.66 ± 0.75 (n=14)
	Tb.Th (µm)	30.08 ± 1.55 (n=8)	31.83 ± 1.58* (n=10)	26.64 ± 0.61 (n=7)	28.97 ± 1.20 (n=14)
	Tb.Sp (µm)	165.9 ± 7.22 (n=8)	140.8 ± 8.7* (n=10)	160.2 ± 5.3 (n=7)	156.4 ± 4.8 (n=14)
	Tb.N (1/mm)	5.170 ± 0.220 (n=8)	5.932 ± 0.292* (n=10)	5.377 ± 0.150 (n=7)	5.449 ± 0.144 (n=14)

Histomorphometry of the proximal tibiae and the L4 vertebral bodies of 12-week old mice. Mean values ± SEM. * p<0.05, **p<0.01, ***p<0.001 vs. *Dmp1-Cre$^-$;Tgif1$^{fl/fl}$* + scr, $^{\#}$p<0.05, $^{\#\#}$p<0.01, $^{\#\#\#}$p<0.001 vs. *Dmp1-Cre$^-$;Tgif1$^{fl/fl}$* + anti-miR-19a/b, § p<0.05, §§ p<0.01, §§§ p<0.001 vs. *Dmp1-Cre^{Tg};Tgif1$^{fl/fl}$* + scr .
