# Supplementary material for: Antagonizing microRNA‐19a/b augments PTH anabolic action and restores bone mass in osteoporosis in mice
Source: EMBO Mol Med. 2022 Oct 4;14(11):e13617. doi: 10.15252/emmm.202013617 (PMC9641424; doi:10.15252/emmm.202013617)
Supplement: Supplementary file 5 — Table EV3 [file EMMM-14-e13617-s013.pdf]

Table EV3. Histomorphometric analysis of the proximal tibiae and vertebrae in Dmp1-Cre^{-};Tgif1$^{fl/fl}$ male mice after single or combined treatment with PTH and anti-miR-19a/b

	Parameters	Male			
		Dmp1-Cre^{-};Tgif1$^{fl/fl}$, scr, vehicle	Dmp1-Cre^{-};Tgif1$^{fl/fl}$, anti-miR-19a/b, vehicle	Dmp1-Cre^{-};Tgif1$^{fl/fl}$, scr, PTH	Dmp1-Cre^{-};Tgif1$^{fl/fl}$, anti-miR-19a/b, PTH
Proximal tibia	BV/TV (%)	7.628 ± 0.581 (n=8)	11.38 ± 0.918* (n=10)	10.68 ± 0.88* (n=12)	15.50 ± 0.90***$^{\#\#\S\S\S}$ (n=10)
	Tb.Th (µm)	28.55 ± 1.08 (n=8)	34.30 ± 1.92 (n=10)	35.06 ± 2.26 (n=12)	40.62 ± 1.59*** (n=10)
	Tb.Sp (µm)	355.0 ± 19.0 (n=8)	279.1 ± 20.9* (n=10)	300.8 ± 15.8* (n=12)	228.7 ± 16.5***§§§ (n=10)
	Tb.N (1/mm)	2.651 ± 0.132 (n=8)	3.308 ± 0.202* (n=10)	3.043 ± 0.132 (n=12)	3.826 ± 0.209***$^{\#\S\S}$ (n=10)
	MS/BS (%)	26.32 ± 1.04 (n=7)	38.48 ± 0.88*** (n=9)	36.30 ± 2.30*** (n=9)	44.61 ± 1.22***$^{\#\#\S\S\S}$ (n=10)
	MAR (µm/day)	1.134 ± 0.056 (n=7)	2.033 ± 0.123*** (n=9)	2.044 ± 0.142*** (n=9)	2.425 ± 0.098***$^{\#\S}$ (n=10)
	BFR/BS (µm^3/µm^2/year)	108.2 ± 4.9 (n=7)	287.1 ± 21.8*** (n=9)	271.3 ± 26.0*** (n=9)	394.2 ± 17.9***$^{\#\#\#\S\S\S}$ (n=10)
	BFR/BV (%/year)	589.4 ± 46.2 (n=7)	1285 ± 97*** (n=9)	1212 ± 110*** (n=9)	1709 ± 103***$^{\#\#\S\S}$ (n=10)
	OV/BV (%)	1.960 ± 0.083 (n=8)	4.866 ± 0.409*** (n=9)	5.473 ± 0.423*** (n=9	7.191 ± 0.559***$^{\#\#\S\S}$ (n=9)
	OS/BS (%)	12.71 ± 0.73 (n=8)	26.58 ± 1.50*** (n=9)	34.03 ± 3.53*** (n=9)	44.31 ± 3.01***$^{\#\#\#\S\S}$ (n=9)
	Ob.S/BS (%)	12.77 ± 0.83 (n=8)	25.98 ± 1.67** (n=9)	33.55 ± 3.56*** (n=9)	44.13 ± 3.15***$^{\#\#\#\S\S}$ (n=9)
	N.Ob/BS (1/mm)	7.762 ± 0.560 (n=8)	16.71 ± 0.83** (n=9)	21.03 ± 2.65*** (n=9)	29.56 ± 2.14***$^{\#\#\#\S\S}$ (n=9)
	ES/BS (%)	0.8232 ± 0.0684 (n=8)	1.042 ± 0.0884 (n=9)	1.431 ± 0.109**$^{\#}$ (n=9)	1.187 ± 0.125* (n=9)
	Oc.S/BS (%)	0.6121 ± 0.0479 (n=8)	0.9226 ± 0.1007* (n=9)	1.302 ± 0.116***$^{\#}$ (n=9)	1.037 ± 0.104* (n=9)
	N.Oc/BS (1/mm)	0.3128 ± 0.0260 (n=8)	0.3861 ± 0.0394 (n=9)	0.5384 ± 0.362**$^{\#}$ (n=9)	0.4179 ± 0.0440§ (n=9)
Vertebral body	BV/TV (%)	15.36 ± 0.45 (n=8)	18.72 ± 1.00* (n=10)	17.80 ± 0.75* (n=12)	19.12 ± 1.43* (n=9)
	Tb.Th (µm)	30.08 ± 1.55 (n=8)	31.83 ± 1.58 (n=10)	33.24 ± 1.23 (n=12)	31.84 ± 1.81 (n=9)
	Tb.Sp (µm)	165.9 ± 7.22 (n=8)	140.8 ± 8.7* (n=10)	154.2 ± 3.7 (n=12)	136.8 ± 6.0** (n=9)
	Tb.N (1/mm)	5.170 ± 0.220 (n=8)	5.932 ± 0.292 (n=10)	5.354 ± 0.096 (n=12)	5.971 ± 0.174* (n=9)

Histomorphometry of the proximal tibiae and the L4 vertebral bodies of 12-week old mice. Mean values ± SEM. * p<0.05, ** p<0.01, *** p<0.001 vs. scr, vehicle; $^{\#}$ p<0.05, $^{\#\#}$ p<0.01, $^{\#\#\#}$ p<0.001 vs. anti-miR-19a/b, vehicle; § p<0.05, §§ p<0.01, §§§ p<0.001 vs. scr, PTH.
