# Supplementary material for: Antagonizing microRNA‐19a/b augments PTH anabolic action and restores bone mass in osteoporosis in mice
Source: EMBO Mol Med. 2022 Oct 4;14(11):e13617. doi: 10.15252/emmm.202013617 (PMC9641424; doi:10.15252/emmm.202013617)
Supplement: Supplementary file 6 — Table EV4 [file EMMM-14-e13617-s015.pdf]

Table EV4. µCT analysis of the distal and midshaft femura of osteoporotic male mice after anti-miR-19a/b treatment

	Parameters	Male		
		sham; scr	ORX; scr	ORX; anti-miR-19a/b
Distal femur	BV/TV (%)	14.10 ± 0.21 (n=10)	6.49 ± 0.53* (n=10)	9.20 ± 0.94*# (n=10)
	Tb.Th (µm)	43.24 ± 2.47 (n=10)	43.02 ± 1.55 (n=10)	46.83 ± 2.13 (n=10)
	Tb.Sp (µm)	333.2 ± 52.1 (n=10)	663.3 ± 57.8* (n=10)	525.5 ± 70.0* (n=10)
	Tb.N (1/mm)	3.083 ± 0.338 (n=10)	1.482 ± 0.086* (n=10)	1.916 ± 0.147* (n=10)
	SMI	22.028 ± 1.98 (n=10)	23.50 ± 1.17 (n=10)	20.67 ± 1.74*# (n=10)
Midshaft femur	Ct.Th (µm)	19.53 ± 0.48 (n=10)	17.42 ± 0.27** (n=10)	17.57 ± 0.41** (n=10)
	Ct.Dens (mg HA/cm^3)	974.2 ± 29.60 (n=10)	1025 ± 30.68 (n=10)	1024 ± 39.96 (n=10)
	Ps.Dm (mm)	190.2 ± 20.50 (n=10)	181.6 ± 22.7 (n=10)	181.0 ± 15.4 (n=10)
	Ps.Pm (mm)	5.972 ± 0.0644 (n=10)	5.693 ± 0.071* (n=10)	5.682 ± 0.048* (n=10)
	Ec.Dm (mm)	1.601 ± 0.018 (n=10)	1.543 ± 0.024 (n=10)	1.535 ± 0.014 (n=10)
	Ec.Pm (mm)	5.027 ± 0.057 (n=10)	4.845 ± 0.076* (n=10)	4.821 ± 0.015* (n=10)

µCT analysis in 18-week old mice. Mean values ± SEM. * p<0.05 vs. sham; scr, ** p<0.05 vs. sham; scr # p<0.05 vs. ORX; scr.
