# Supplementary material for: Antagonizing microRNA‐19a/b augments PTH anabolic action and restores bone mass in osteoporosis in mice
Source: EMBO Mol Med. 2022 Oct 4;14(11):e13617. doi: 10.15252/emmm.202013617 (PMC9641424; doi:10.15252/emmm.202013617)
Supplement: Supplementary file 7 — Table EV5 [file EMMM-14-e13617-s010.pdf]

Table EV5. µCT analysis of the distal and midshaft femura of osteoporotic female mice after anti-miR-19a/b treatment

	Parameters	Female		
		sham; scr	OVX; scr	OVX; anti-miR-19a/b
Distal femur	BV/TV (%)	12.46 ± 0.71 (n=10)	8.218 ± 0.49* (n=6)	13.35 ± 1.27## (n=8)
	Tb.Th (µm)	49.27 ± 1.22 (n=10)	43.42 ± 1.40* (n=6)	50.11 ± 1.59# (n=8)
	Tb.Sp (µm)	355.3 ± 17.69 (n=10)	492.8 ± 25.66*** (n=6)	343.4 ± 26.80*### (n=8)
	Tb.N (1/mm)	3.083 ± 0.338 (n=10)	1.482 ± 0.086** (n=6)	1.916 ± 0.147## (n=8)
	SMI	20.38 ± 0.954 (n=10)	21.77 ± 0.786 (n=6)	20.46 ± 0.748 (n=8)
Midshaft femur	Ct.Th (µm)	19.17 ± 0.236 (n=10)	18.25 ± 0.208** (n=6)	18.71 ± 0.318** (n=8)
	Ct.Dens (mg HA/cm^3)	1033 ± 29.60 (n=10)	1048 ± 21.67 (n=6)	1055 ± 14.61 (n=8)
	Ps.Dm (mm)	187.7 ± 27.50 (n=10)	176.4 ± 16.40* (n=6)	186.4 ± 30.4# (n=8)
	Ps.Pm (mm)	5.894 ± 0.084 (n=10)	5.540 ± 0.049* (n=6)	5.853 ± 0.095# (n=10)
	Ec.Dm (mm)	1.594 ± 0.030 (n=10)	1.496 ± 0.044 (n=6)	1.582 ± 0.084 (n=10)
	Ec.Pm (mm)	5.006 ± 0.093 (n=10)	4.668 ± 0.014 (n=6)	4.969 ± 0.027 (n=10)

µCT analysis in 19-week old mice. Mean values ± SEM. * p<0.05, ** p<0.01, *** p<0.001 vs. sham; scr # p<0.05, ## p<0.01, ### p<0.001 vs. OVX; scr.
