# Supplementary material for: Antagonizing microRNA‐19a/b augments PTH anabolic action and restores bone mass in osteoporosis in mice
Source: EMBO Mol Med. 2022 Oct 4;14(11):e13617. doi: 10.15252/emmm.202013617 (PMC9641424; doi:10.15252/emmm.202013617)
Supplement: Supplementary file 8 — Table EV6 [file EMMM-14-e13617-s012.pdf]

Table EV6. Sequences of oligonucleotides used for gene expression analysis and of GapmeRs used for gene silencing

Oligonucleotide sequences

**Name**	**Sequence (5'-3')**
Runx2 F	TCCACAAGGAGAGAGTCAGATTACAG
Runx2 R	CAGAAGTCAGAGGTGGCAGTGTCATC
Col1a1 F	CGATGGATTCCCGTTCGAGT
Col1a1 R	CGATCTCGTTGGATCCCTGG
Tbp F	GCTCTGGAATTGTACCGCAGC
Tbp R	CTCTTGGCTCCTGTGCACAC
Gapdh F	TGCACCACCAACTGCTTAG
Gapdh R	GGATGCAGGGATGATGTTC
U6 F	CGCAAGGATGACACGCAAATTC
miR19a F	TGTGCAAATCCATGCAAAACTGA
miR19b F	TGTGCAAATCTATGCAAAACTGA
b2m F	CTGCTACGTAACACAGTTCCACCC
b2m R	CATGATGCTTGATCACATGTCTCG
β-actin1 F	CGTAAAGACCTCTATGCCAACAC
β-actin1 R	CACATCTGCTGGAAGGTGGAC
β-actin2 F	TGTACCCAGGCATTGCTGAC
β-actin2 R	AGGGTGTAAAACGCAGCTCA
Trap F	GGTTCCAGGAGACCTTTGAGG
Trap R	AGCGCAAACGGTAGTAAGGG
CatK F	CAGTTTTACAGCAGAGGTGTGT
CatK R	TGGCTGGAATCACATCTTGGG
Rankl F	GAACTGCAACACATTGTGGGG
Rankl R	GTGACTTTATGGGAACCCGATG
PP2A-Cβ F	GAACCAAGTGCGGACGCTG
PP2A-Cβ R	CCTCTGTCTACATAGTCCCCC
Tgif1 F	CAGACACACCTGTCCACACTA
Tgif1 R	GGAATGAAATGGGCTCTCTTCT
Inf-γ F	CCACGGCACAGTCATTGAAAG
Inf-γ R	GCTGATGGCCTGATTGTCTTTC
Tnf-α F	GAACTGGCGAAGAGGCACTC
Tnf-α R	CTGATGAGAGGGAGGCCATTTG
Ccl5 F	CTCACCATATGGCTCGGACAC
Ccl5 R	CACACACTTGGCGGTTCCTTC
Ccl2 F	TTAAAAACCTGGATCGGAACCAA
Ccl2 R	GCATTAGCTTCAGATTTACGGGT
F4/80 F	ACTGTCTGCTCAACCGTCAGGT
F4/80 R	GGAATGGGAGCTAAGGTCAGTCT
Il1 F	GACCTTCCAGGATGAGGACA
Il1 R	AGGCCACAGGTATTTTGTCG

Il1b F	CAGAAGTACCTGAGCTCGCC
Il1b R	AGATTCGTAGCTGGATGCCG
Cd11b F	GCATCTAAGCCAGCTGGTGAAC
Cd11b R	CCAGCAAGGGACCATTAGAGG
Cd11c F	GCTGTGATGAGCCAGCTTCAG
Cd11c R	GGGTGGTGAACAGTTCTGTTATGAC
Eph2B F	CAGACCAGCATCAAGGAAAAG
Eph2B R	ATGTGTCCGCTGGTGTAGTG
Eph4B F	CACCCAGCAGCTTGATCCTG
Eph4B R	ACCAGGACCACACCCACAAC
Ephrin2B F	GCCAGGAATCACGGTCCAAC
Ephrin2B R	GATGCATCCTGATGCGATCCC
IL6 F	GAGCCCACCAAGAACGATAG
IL6 R	GGTTGTCACCAGCATCAGTC
IL11 F	CGGCAACTAGCTGCACAGATG
IL11 R	CTCCAGAGTCTTTAGGGAAGG
IL18 F	CCAGCATCAGGACAAAGAAA
IL18 R	TACAGTGAAGTCGGCCAAAG
Igf1 F	GATGCTCTTCAGTTCGTGTGTG
Igf1 R	GCTCCGGAAGCAACACTCAT
Igf2 F	CACGCTTCAGTTTGTCTGTTCGG
Igf2 R	CAGCACTCTTCCACGATGCCAC
Igfbp2 F	CTGTGTTCCGGGAGAAGGTC
Igfbp2 R	GGGATGTGCAGGGAGTAGAG
Igfbp3 F	CAAGTTCCATCCACTCCATGC
Igfbp3 R	CCTCTGGGACTCAGCACATTG
Igfbp5 F	CCTGCACCTGAGATGAGACAG
Igfbp5 R	CACCAGCAGATGCCACGTTTG

F=forward, R=reverse

GapmeR sequences

**Target name**	**Sequence (5'-3')**
Tgif1	ACAGTCTTGGAACATA
PP2A-Cβ	TGAGACCGTTGGCATG
scrambled control	AACACGTCTATACGC
