# Supplementary figures and images for: Antagonizing microRNA‐19a/b augments PTH anabolic action and restores bone mass in osteoporosis in mice
Source: EMBO Mol Med. 2022 Oct 4;14(11):e13617. doi: 10.15252/emmm.202013617 (PMC9641424; doi:10.15252/emmm.202013617)

Source Data to Fig 3

A

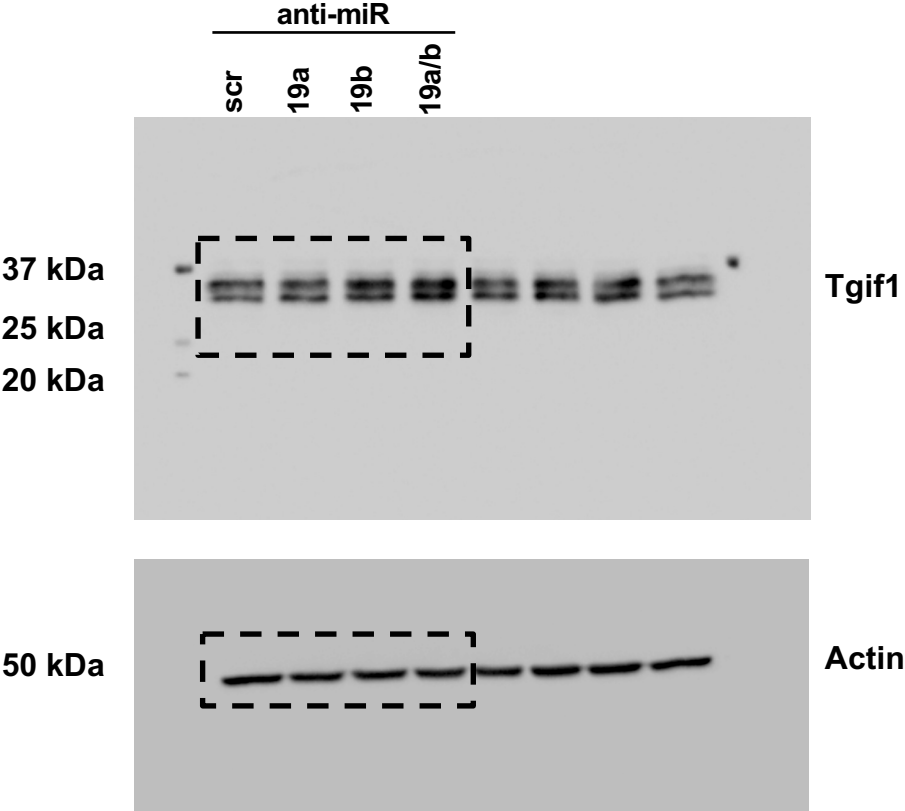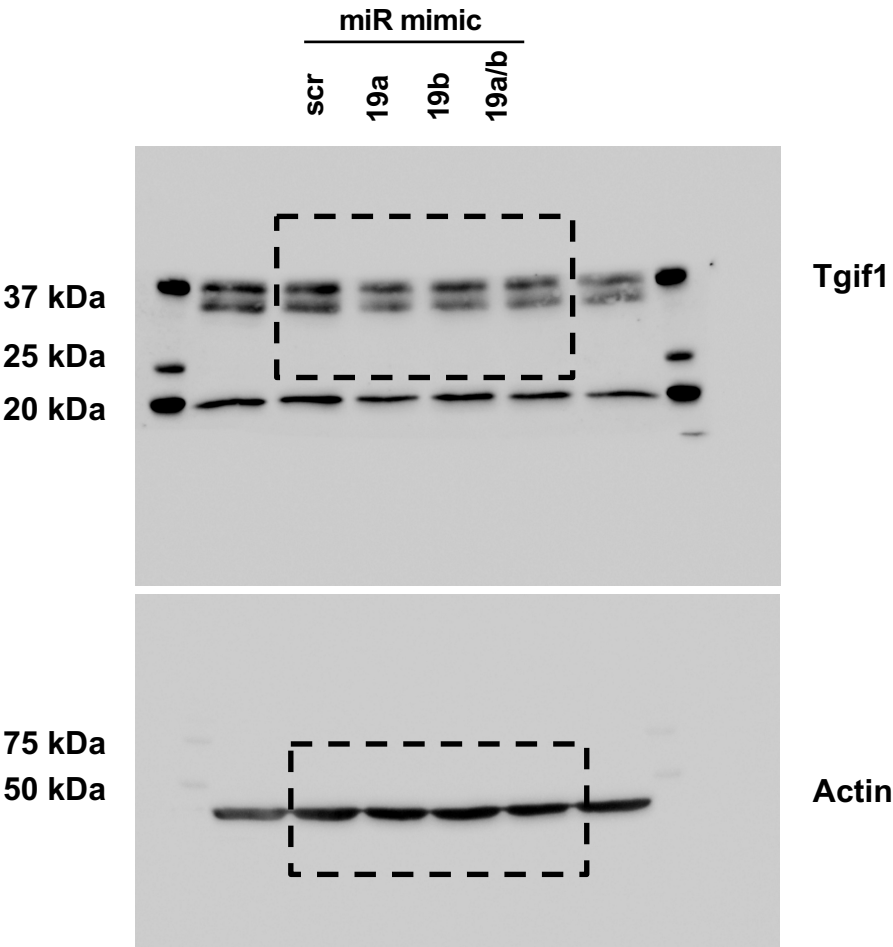

Supplement: Supplementary file 12 — Source Data for Figure 3 [file EMMM-14-e13617-s014.pdf]

A

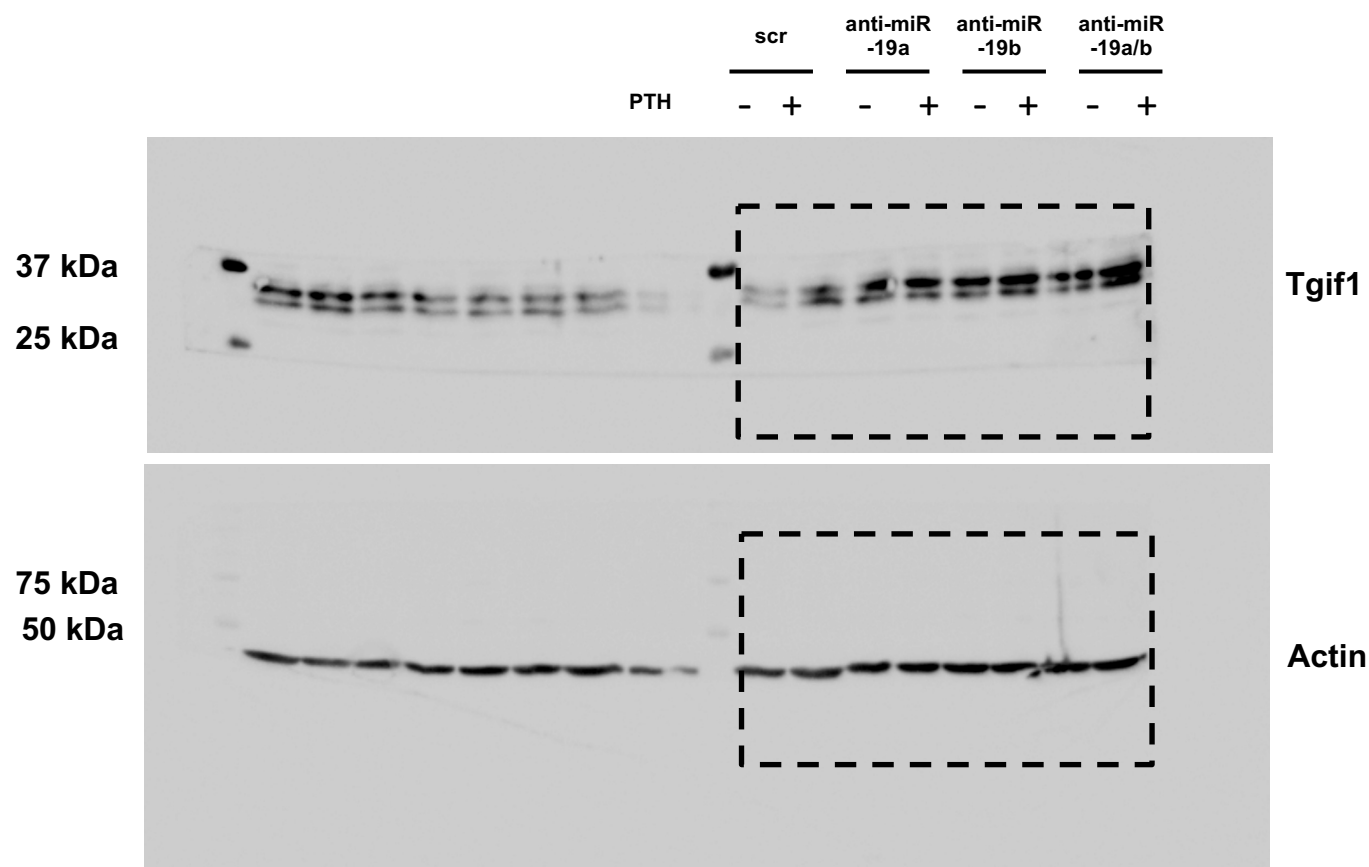

Supplement: Supplementary file 13 — Source Data for Figure 4 [file EMMM-14-e13617-s002.pdf]

Source Data to Fig 6

L

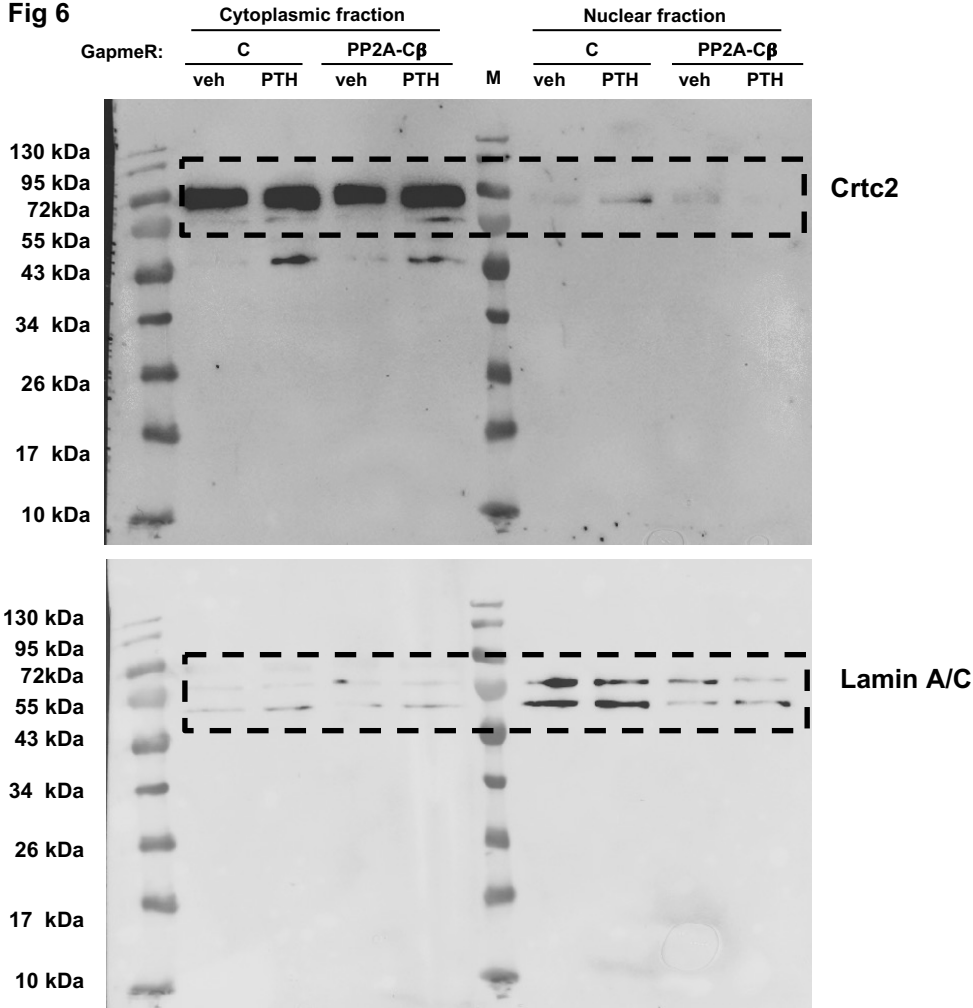

M

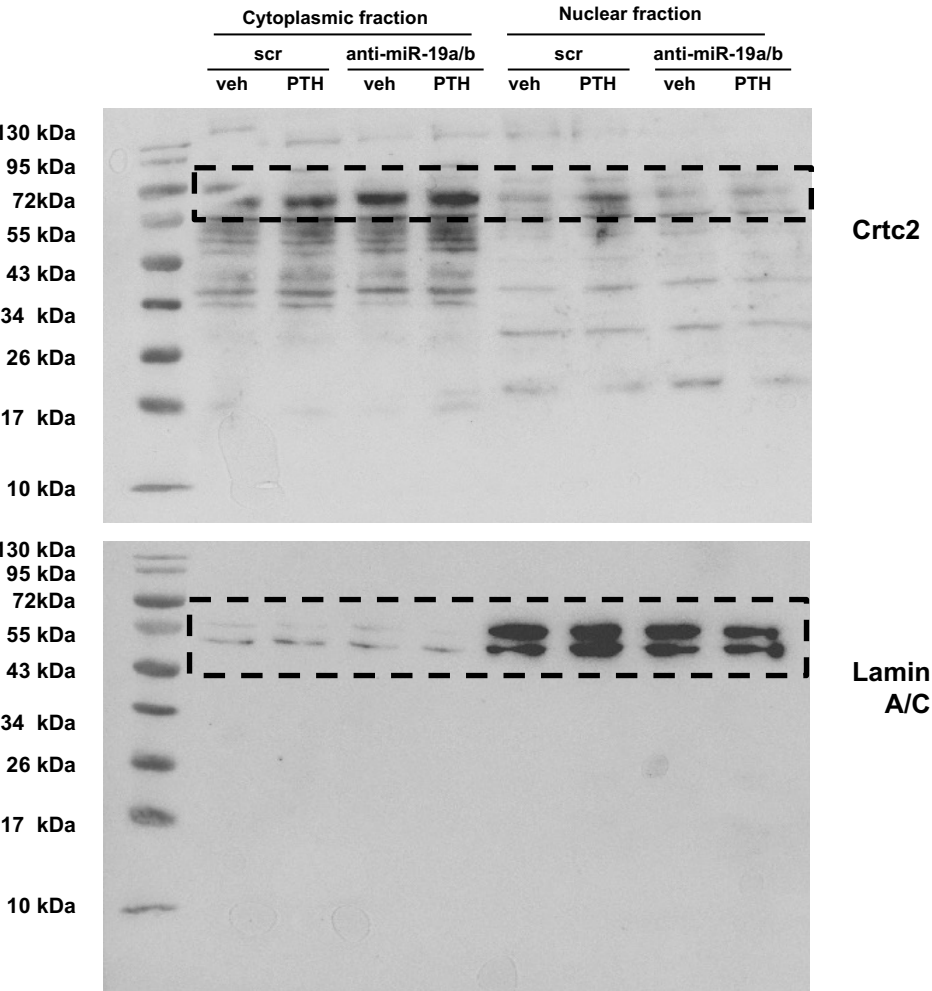

Supplement: Supplementary file 14 — Source Data for Figure 6 [file EMMM-14-e13617-s008.pdf]
