# Supplementary material for: Antagonizing microRNA‐19a/b augments PTH anabolic action and restores bone mass in osteoporosis in mice
Source: EMBO Mol Med. 2022 Oct 4;14(11):e13617. doi: 10.15252/emmm.202013617 (PMC9641424; doi:10.15252/emmm.202013617)
Supplement: Supplementary file 15 [file EMMM-14-e13617-s009.pdf]

EMBO Molecular Medicine

Antagonizing miRNA-19a/b augments PTH anabolic action and restores bone mass in osteoporosis in mice

Hanna Taipaleenmäki, Hiroaki Saito, Saskia Schröder, Miki Maeda, Ramona Mettler, Matthias Ring, Ewa Rollmann, Andreas Gasser, Carl Haasper, Thorsten Gehrke, Alexander Weiss, Steffen Grimm, and Eric Hesse
DOI: 10.15252/emmm.202013617

Corresponding authors: Eric Hesse (Eric.Hesse@med.uni-muenchen.de) , Hanna Taipaleenmäki (hanna.taipaleenmaeki@med.uni-muenchen.de)

Review Timeline:

Submission Date:	20th Oct 20
Editorial Decision:	9th Nov 20
Revision Received:	31st May 22
Editorial Decision:	20th Jun 22
Revision Received:	30th Aug 22
Editorial Decision:	5th Sep 22
Revision Received:	12th Sep 22
Accepted:	13th Sep 22

Editor: Zeljko Durdevic

Transaction Report:

(Note: With the exception of the correction of typographical or spelling errors that could be a source of ambiguity, letters and reports are not edited. Depending on transfer agreements, referee reports obtained elsewhere may or may not be included in this compilation. Referee reports are anonymous unless the Referee chooses to sign their reports.)

9th Nov 2020

Dear Prof. Hesse,

Thank you for the submission of your manuscript to EMBO Molecular Medicine. We have now received feedback from the three reviewers who agreed to evaluate your manuscript. As you will see from the reports below, the referees acknowledge the interest and novelty of the study but also raise serious and partially overlapping concerns that should be addressed in a major revision. Particular attention should be given to the detailed analysis of the osteoclast phenotype.

Addressing the reviewers' concerns in full will be necessary for further considering the manuscript in our journal, and acceptance of the manuscript will entail a second round of review. EMBO Molecular Medicine encourages a single round of revision only and therefore, acceptance or rejection of the manuscript will depend on the completeness of your responses included in the next, final version of the manuscript. For this reason, and to save you from any frustrations in the end, I would strongly advise against returning an incomplete revision.

Considering the extent of the revision, I am happy to extend the revisions time to 6 months. We realize that the current situation is exceptional on the account of the COVID-19/SARS-CoV-2 pandemic. Therefore, please let us know if you need more than six months to revise the manuscript.

I look forward to receiving your revised manuscript.

Yours sincerely,

Zeljko Durdevic

Zeljko Durdevic
Editor
EMBO Molecular Medicine

***** Reviewer's comments *****

Referee #1 (Remarks for Author):

The authors investigated the role of miR-19a/b in bone tissue and the effects of anti-miR-19a/b treatment on bone in various scenarios. They found that anti-miR-10a/b treatment is able to increase bone mass alone and further augment the effect of PTH osteoanabolic treatment. One proposed mechanism might be via Tgif-1 signaling. The research topic is very interesting and clinically relevant, the manuscript is well written and the authors used appropriate methods. I especially like the translational relevance of this work since the authors also showed some human data. However, there are some points in this work which need some clarification before publication. Please see my specific comments below.

Specific comments

Abstract: very well written

Introduction: clear structure and very well written

Results:

Inhibition of miR-19a/B increases bone mass:

- Fig. 1: The authors wrote that osteoclast activity was not affected, but this seems to indeed be the case according to the data (OcS/BS is sign. increased in the spine and tibia). Please correct that in the text.

- Fig 1 and Suppl. Fig 2: The authors choose to analyze tibiae from 8 weeks old mice (which seems to have very few trabecular structure in the metaphysis) and femurs from 12 weeks old mice which show a lot of trabecular structure. Could the authors comment on the different ages and why these ages and locations were chosen for analysis?

Anti-miR-19a/b treatment increases bone mass in a Tgif1-dependent manner

- The authors used the Dmp1-Cre model to prove that the effects of anti-miR treatment is Tgif1-mediated. However, only very mature osteoblasts and osteocytes express Dmp1 in the intact bone. This might be the reason why many treatment effects are still significant in Cre+ mice. Would the authors expect the same results using a Cre model earlier in the osteoblast lineage, like Runx2-Cre or OC-Cre? Why did the authors choose the Dmp1-Cre mouse?

- The finding that anti-miR treatment reduces resorption in the context of PTH treatment and OVX is very interesting also in the light that OcS/BS was increased in mice treated with anti-miR alone. These findings should be displayed in the main figures of the manuscript instead of the suppl. figures. Furthermore, some more mechanistic data on that topic is needed. The authors should investigate if the effect on osteoclasts is a direct effect or maybe an indirect effect over osteoblast-secreted RANKL. For that, further in vitro experiments are needed (influence of anti-miR treatment/PTH/treatment/E2 deficiency on osteoclasts directly, or coculture/supernatant experiments with osteoblasts). Also, immunohistochemical stainings for analyzing osteoclast-related markers in the bone sections of the treated animals would be important (RANKL, CatK).

- It is known that OVX mice displayed a chronic low-grade inflammation and that inflammatory cytokines might also influence osteoblasts and osteoclasts. Did the authors analyze the inflammatory state of the animals and the effect of miR-10a/b?

- It would be interesting to see the effects of anti-miR-19a/b treatment also in combination with Wnt activation in vivo, although I know that this would be a lot of extra work. But maybe the authors have some data about that topic also in vivo.

- Is Tgif-1 expression also altered by sex-steroid deficiency?

Discussion:

The authors should further discuss the different effects of anti-miR-10a/b treatment on osteoclasts if given alone or in combination with PTH or after OVX.

Methods

Detailed description would allow repetition of the work.

Referee #2 (Remarks for Author):

The manuscript of Taipaleemäki et al. shows that skeletal expression of miR-19a/b is reduced by treatment with PTH or anti-sclerostin antibody. Moreover, using cell culture and animal models, they identify TGIF as a downstream mediator of miR-19a/b that mediates a bone-anabolic response via increasing bone formation. Finally, they show that antagonizing miR-19a/b enhances the bone anabolic effect of PTH and enhances bone mass in models of sex hormone deficiency, thus, proposing it as a novel treatment strategy for osteoporosis. Overall, this is an easy to follow manuscript with a logical structure, sufficient methodological details to replicate the study, and sound results that are presented in a comprehendible way. The topic of finding new drugs for osteoporosis treatment is of great interest.

Major comments:
1. The rescue of the bone phenotype induced by miR-19a/b knockdown via TGIF deficiency in osteogenic cells is quite intriguing. TGIF seems to play no role on the increase in bone formation parameters in mice with a miR-19a/b knockdown. However, bone resorption parameters are higher, if TGIF in Dmp1-expressing cells is not present. Thus, given that TGIF is only deleted in osteogenic cells, it seems these cells exert stimulatory signals to osteoclasts. Have the authors checked RANKL/OPG and does TGIF in osteogenic cells alter their communication to osteoclasts? Would co-cultures be useful to address this communication? Is miR-19a/b expressed in osteoclasts?
2. Along those lines, how is the modulation of Wnt signaling in these cells (cells without TGIF and with anti-miR-19a/b)? This phenotype resembles lack of Wnt signaling in osteogenic cells and the abnormal crosstalk to osteoclasts. This is also displayed again by the actions shown in Suppl. Fig. 8, which reminds one more of the effects of anti-sclerostin therapy than PTH (due to the uncoupled effect on bone remodeling). Does Sost (or Dkk1 or another Wnt inhibitor) play a role in mediating the effects of miR-19a/b?
3. Overall, it appears to this reviewer, as if most of the effects of antagonizing miR-19a/b (after PTH treatment and OVX/ORX, for

some reason not in steady-state) does not stem from the increase in bone formation, but from the indirect effects on suppressing bone resorption. I think this should be stressed more throughout the manuscript. I think this is also the reason why the treatment works so nicely in OVX and ORX, which are mostly osteoclast driven. This goes in line with the abstract and introduction, which I would rather not stress on age-related osteoporosis, but post-menopausal osteoporosis, as these are also the models the authors use later on. Also, in some figures, it may be more useful to show one bone site and the histology data next to them, than burry them in the supplementary figures. This would immediately show the cellular mechanisms of action without needing to go to the supplement.
4. Synergism of anti-miR-19a/b treatment and PTH is more pronounced at the tibia. No additive effects are observed in the spine. Do the authors have an explanation for this? Is the expression of miR-19 different? Usually, PTH has more pronounced effects on the spine than the hip/femur. How are these different effects between miR-19a/b and PTH reconciled?
5. The patient cohort should be better described. Besides being old and having OA, what other diseases/medications were present?

Minor comments:
6. Can the authors explain/comment on the differences between miR-19a and b? The expression and regulation seems to overlap to a significant amount. Is this expected or unusual?
7. It is not entirely clear why housekeeping genes were used to look at potential adverse effects on different organs.
8. The normalization in some figures including Fig. 2B/C makes it difficult to appreciate the level of miR-19a/b expression in the different tissues. It would be more informative to see the absolute values to get an impression of how much miR-19 is expressed in the various tissues.
9. Full Western blots should be provided as supplementary figures. Western blots should be semi-quantified.
10. Paragraph two of the discussion could be shortened.
11. Two-way ANOVA should be used for Fig. 3C-F and Fig. 4B-E.
12. It could be explained a little bit better how TGIF was chosen to be further investigated as a downstream target of miR-19a/b. I am aware that the authors have studied TGIF a lot, but for sure other targets of miR-19a/b are available, perhaps including components of the Wnt signaling pathway?

Referee #3 (Comments on Novelty/Model System for Author):

At this stage, the paper presents insufficient detail on the methods used to be confident about the data presented. If they are able to address these concerns, the model and methods of measurement may prove to be adequate.

Referee #3 (Remarks for Author):

This work from Taipaleenmaki provides interesting new data to show that inhibition of miR-19a/b may provide a novel pathway to promote bone formation which may provide effects additional to PTH-based anabolic therapy. The introduction is very clearly written. Generally, the data is well presented, but the methods are poorly described, and some model systems not clearly defined. In some cases, it is difficult to be confident of the conclusions drawn without this additional information.

Major concerns:
1. Method details are lacking for many of the experiments, which makes it difficult to judge the validity of the data. Some examples: (1) the vehicle used, and the time point at which vehicle samples were collected for the data shown in Figure 1A, (2) the bioinformatic analysis used for the data shown in Figure 1A, (3) regions used for histomorphometry and micro-computed tomography, (4) thresholding levels used for micro-computed tomography, (5) sex of mice used for the experiment described on p7 (Figure 1), (6) details of in silico analysis described on p9, line 9, (7) age and sex of Dmp1-CreTgif mice and duration of treatment used for data shown in Figure 3, (8) sex and age of mice for PTH + anti-miR studies shown in Figure 4 (9)
2. Please provide evidence that anti-miR19a, anti-miR19b, and anti-miR19a/b were successful at achieving knockdown in the cell culture experiment shown in Figure 1D, and provide quantitation of the alkaline phosphatase assay. A single image is not sufficient evidence of altered osteoblast activity. Ideally mRNA markers of osteoblast differentiation should also be reported for this experiment. Without this, the authors cannot really claim that "differentiation was enhanced".
3. The evidence that Tgif is required for the anabolic action of miR-19a/b blockade is not very convincing and does not add to the story. This data could be removed and the paper would be improved. The substantial increase in osteoblast markers in response to miR-19a/b blockade in these mice, and the data showing a lack of increase in BV/TV is not normally distributed. The evidence is very poor, and should be removed.
4. Throughout the study, it seems that all of the mice used are young, growing animals. These are not a good model of bone remodeling, or a good model of adult osteoporosis. This is a major limitation which should be discussed in detail in the paper.
5. The patient data is very poorly described, and based on extremely low numbers. I recommend removing it. It adds little to the work. Without explaining the tissue collected, and showing the micro-CT data, it is not very strong evidence at all, and raises more questions than it answers.
6. The authors repeatedly use the term "osteoporosis" to describe low bone mass in mice. This is inaccurate, and should be changed. Mice do not suffer from osteoporosis; it is more accurate to say "low bone mass", or even "gonadectomy-induced bone loss".

7. It is surprising that miR-19a/b inhibition and PTH have additive effects when PTH itself already reduces miR-19a/b. This suggests they may have different modes of action, and that PTH anabolic effect is not dependent on miR-10a/b. Some discussion of this would be appropriate.

Additional comments:
1. p3, line 18. Abaloparatide is not the same as the first 34 amino acids of PTHrP; it is a modified form of the 34 amino acid protein.
2. Figure 1A, most of the regions of the Venn diagram contain no information. Is that because no unique genes were regulated by veh, PTH and Scl-Ab?
3. ObS/BS is not a marker of osteoclast size. Please rephrase (p11, line 6).
4. The authors state that anti-miR treatment "reduced activated bone resorption" but present no evidence to support this (p11, line 12).
5. It would help the reader if the methods could be reported in the same order as the experimental data shown.
6. Please describe whether bone samples were flushed of marrow before mRNA was generated.

Response to reviewers

We appreciate the careful review provided by the three referees and for very useful expert comments that have further improved the quality of the manuscript. Below we provide a point-by-point response to each question. Overall, we belief that all aspects have been taken care of in a reasonable manner.

Referee #1 (Remarks for Author):

The authors investigated the role of miR-19a/b in bone tissue and the effects of anti-miR-19a/b treatment on bone in various scenarios. They found that anti-miR-10a/b treatment is able to increase bone mass alone and further augment the effect of PTH osteoanabolic treatment. One proposed mechanism might be via Tgif-1 signaling. The research topic is very interesting and clinically relevant, the manuscript is well written and the authors used appropriate methods. I especially like the translational relevance of this work since the authors also showed some human data. However, there are some points in this work which need some clarification before publication. Please see my specific comments below.

We thank the reviewer for appreciating the interesting and clinically relevant nature of our manuscript that is perceived to be well written and based on appropriate models. It is reassuring to hear that the reviewer values the human data since they support the translational relevance of the work. We are delighted to provide clarification on the remaining points the reviewer has, which we were able to address fully.

Specific comments

Abstract: very well written

Introduction: clear structure and very well written

Results:

Inhibition of miR-19a/B increases bone mass:

- ***Fig. 1: The authors wrote that osteoclast activity was not affected, but this seems to indeed be the case according to the data (OcS/BS is sign. increased in the spine and tibia). Please correct that in the text.***

We thank the reviewer for indicating this. We added the notion to the text that Oc.S/BS is increased, while all other osteoclast-related parameters are unchanged (page 7, line 16-19).

- ***Fig 1 and Suppl. Fig 2: The authors choose to analyze tibiae from 8 weeks old mice (which seems to have very few trabecular structure in the metaphysis) and femurs from 12 weeks old mice which show a lot of trabecular structure. Could the authors comment on the different ages and why these ages and locations were chosen for analysis?***

We apologize for this misunderstanding. Treatments like anti-miR injections or OVX/ORX surgeries were always performed at 8-weeks of age. Analysis of bones at either location (tibia, femur, vertebra) was done at 12-weeks of age after 4-weeks of treatment with anti-miR-19a/b or PTH or at older ages in the ORX (19-week-old) and OVX (18-week-old) experiments. Thus, there are no differences between age and location regarding the initiation of each experiment.

We have carefully revised the manuscript to ensure that the ages of the animals are stated correctly.

*- **The authors used the Dmp1-Cre model to prove that the effects of anti-miR treatment is Tgif1-mediated. However, only very mature osteoblasts and osteocytes express Dmp1 in the intact bone. This might be the reason why many treatment effects are still significant in Cre+ mice. Would the authors expect the same results using a Cre model earlier in the osteoblast lineage, like Runx2-Cre or OC-Cre? Why did the authors choose the Dmp1-Cre mouse?***

This is a very good point. We agree with the reviewer that in theory, Dmp1 promoter-driven Cre expression should delete Tgif1 only in a small set of mature osteoblasts and in osteocytes, while other Cre strains (e.g. Osx-Cre, Col1-3.6 kb-Cre, Col1-2.3 kb-Cre or Runx2-Cre) would be expected to delete Tgif1 in earlier stage osteoblasts and therefore in a larger number of cells.

However, the artificial promoters or promoter fragments used are not as specific and precise in deleting at a defined stage of osteoblast differentiation as they are expected to be. For instance, other groups have performed a more detailed investigation of the Dmp1 promoter-driven transgene expression. Al-Jazzar and colleagues injected diphtheria toxin in mice expressing the human diphtheria toxin receptor transgene to ablate osteocytes. Unexpectedly, animals presented histopathological diphtheria toxin-induced changes in multiple organs with rapid deterioration of the health of the animals (*Al-Jazzar A., et al., Int. J. Mol. Sci., 2017*). These results indicate an ectopic activity of the Dmp1 promoter. Similar non-specific deletion events have been described for the Osx-Cre mouse line (*Chen J. et al., Plos One, 2014*). This demonstrates the technical limitations of these model systems.

Importantly, we have performed osteoblast-targeted deletion of Tgif1 using Osx-Cre and Dmp1-Cre lines and observed the identical low bone turnover phenotype (*Saito H. et al., Nat Commun., 2019*). This observation indicates that the extend and the pattern of the deletion is quite comparable.

Based on these findings reported by our group and others, we would not have expected to observe markedly different treatment effects using other Cre lines. The observation that some treatment effects are still significant in Dmp1-Cre mice is most likely because miR-19a/b might have additional targets, leading to Tgif1-independent effects although Tgif1 is essential for the increase in bone mass in response to anti-miR-19a/b treatment.

Among the available Cre lines, we chose Dmp1-Cre mice because this strain does not cause non-specific effects of the Cre recombinase (which is known for the Osx-Cre line, *Huang et al., Transgenic Res., 2015*) and because mice of this strain are robust to breed.

*- **The finding that anti-miR treatment reduces resorption in the context of PTH treatment and OVX is very interesting also in the light that OcS/BS was increased in mice treated with anti-miR alone. These findings should be displayed in the main figures of the manuscript instead of the suppl. figures. Furthermore, some more mechanistic data on that topic is needed. The authors should investigate if the effect on osteoclasts is a direct effect or maybe an indirect effect over osteoblast-secreted RANKL. For that, further in vitro experiments are needed (influence of anti-miR treatment/PTH/treatment/E2 deficiency on osteoclasts directly, or coculture/supernatant experiments with osteoblasts). Also, immunohistochemical stainings for analyzing osteoclast-related markers in the bone sections of the treated animals would be important (RANKL, CatK).***

We agree with the reviewer on these very important aspects. As suggested, we moved the data demonstrating the anti-resorptive effect of anti-miR-19a/b treatment in the context of PTH-stimulation and OVX to the main figures (Figures 4 and 5).

To provide more mechanistic insight in how anti-miR-19a/b exerts anti-resorptive effects under conditions of high bone turnover, we conducted a comprehensive set of new experiments. First, we confirmed that anti-miR-19a/b treatment reduced the enhanced activation and differentiation of osteoclast obtained from OVX mice (Fig. 5 I, J). As suggested by the reviewer, we quantified osteoclasts in bone sections of treated animals using TRAP staining and confirmed that the PTH-induced increase in osteoclast number is returned to the level of control by anti-miR-19a/b treatment (Appendix Figure S3). However, these findings and the notion that endogenous expression of miR-19a and miR-19b increase during osteoclast differentiation (Fig. 6 A, B) did not rule out the possibility of direct effects of anti-miR-19a/b on osteoclast differentiation. To address this question, we antagonized endogenous miR-19a and miR-19b in osteoclasts (Appendix Figure S5A), which did not affect the appearance or number of TRAP-positive multinucleated cells (MNCs) (Fig. 6 C, D). Together, these findings confirm the inhibitory effect of anti-miR-19a/b on osteoclast activity *ex vivo* and *in vivo* but indicate an indirect inhibitory effect of anti-miR19a/b on osteoclasts.

To elucidate the underlying molecular mechanism and as suggested by the reviewer, we conducted supernatant experiments in which we transiently transfected cells of the osteoblastic cell line Ocy454 with anti-miR-19a/b or scr control and stimulated these cells with PTH or vehicle. Supernatant was then used to stimulate the differentiation of bone marrow macrophages into osteoclasts (Appendix Figure S5B). TRAP staining and counting of MNCs confirmed that anti-miR-19a/b alone does not have a direct effect on osteoclast differentiation but prevented the PTH-induced increase in TRAP-positive MNCs (Fig. 6 E, F). To identify the osteoblast-derived soluble factor affected by anti-miR-19a/b, we first excluded a larger number of possible targets (Appendix Figure S5C) and then uncovered that anti-miR-19a/b prevents the PTH-induced increase in Rankl mRNA expression in osteoblasts and RANKL protein abundance in the supernatant of cultured osteoblasts (Fig. 6 G, H).

We then elucidated that anti-miR-19a/b attenuates the PTH-induced increase in Rankl expression by augmenting the abundance of Tgif1. Tgif1 antagonizes PP2A-Cβ, which prevents the nuclear translocation of Crtc2, leading to a lower expression of Rankl in osteoblasts and osteocytes (Fig. 6 I-M). Consistently, under conditions of *in vivo* and *in vitro* sex steroid-deficiency, expression of endogenous miR-19a, miR-19b and PP2A-Cβ was increased, while Tgif1 expression was decreased (Fig. 7 A-G, Appendix Figure S6 A-C). Finally, estrogen-deficiency-induced increase in Rankl expression was returned to the level of control by anti-miR-19a/b (Fig. 7 H).

In summary, the comprehensive and consistent novel *in vivo* and *in vitro* findings let us to conclude that under conditions of high bone turn-over as for instance upon PTH treatment or sex-steroid deficiency, anti-miR-19a/b reduces the increased bone resorption by augmenting the abundance of Tgif1, which in turn antagonizes the expression of PP2A-Cβ thereby reducing the nuclear translocation of Crct2 and diminishing the expression of Rankl (Fig. 7 P).

- It is known that OVX mice displayed a chronic low-grade inflammation and that inflammatory cytokines might also influence osteoblasts and osteoclasts. Did the authors analyze the inflammatory state of the animals and the effect of miR-10a/b?

We agree with the reviewer that inflammatory processes can influence bone remodeling and sex steroid deficiency-induced bone loss. We therefore quantified the expression of a comprehensive panel of inflammatory cytokines in bones. However, we did not observe any alteration in the OVX group nor in the group of mice that were treated with anti-miR-19a/b

compared to control animals. We therefore concluded that inflammatory processes are not a major driver of bone loss or pharmacologically-induced gain of bone mass. The findings are reported in the revised manuscript (page 13, line 5-9 and Fig. EV 5G).

- It would be interesting to see the effects of anti-miR-19a/b treatment also in combination with Wnt activation in vivo, although I know that this would be a lot of extra work. But maybe the authors have some data about that topic also in vivo.

We agree with the reviewer that it is an interesting question if anti-miR-19a/b influences the activity of canonical Wnt signaling. To address this question, we transfected cells of the osteoblast-like cell lines ST2 and MC3T3-E1 with anti-miR-19a/b or scrambled control as well as with the TopFlash reporter gene construct and stimulated these cells with Wnt3a or vehicle. However, we did not observe any effect of miR-19a/b inhibition on reporter gene activity (see data below, panel A, B). In addition, quantification of the expression of the Wnt target gene Tcf1 in MC3T3-E1 cells did also not reveal any effect of anti-miR-19a/b on Wnt3a-mediated Wnt pathway activation (panel C). Furthermore, inhibition of miR-19a/b did not alter the endogenous expression of the Wnt target genes Axin2, Lef1 or Tcf1 in MC3T3-E1 cells (panel D-F). Finally, expression of the Wnt inhibitors Dkk1 and Sost in UMR-106 cells was also not affected by anti-miR-19a/b treatment (panel G, H). In conclusion, the combined *in vitro* evidence does not suggest any effect on Wnt pathway activation by anti-miR-19a/b. Considering these findings and to better preserve our resources in support of further investigating the mechanisms by which anti-miR-19a/b acts anti-resorptive in high bone turnover conditions as central part of the revision of this manuscript, we reasoned not to conduct additional *in vivo* experiments in this direction. We hope this reviewer agrees with this evidence-based rationale. The results demonstrating the lack of Wnt activation by anti-miR-19a/b are presented in this letter but not in the manuscript. However, we are open to present these finding to the readership if suggested by the reviewer.

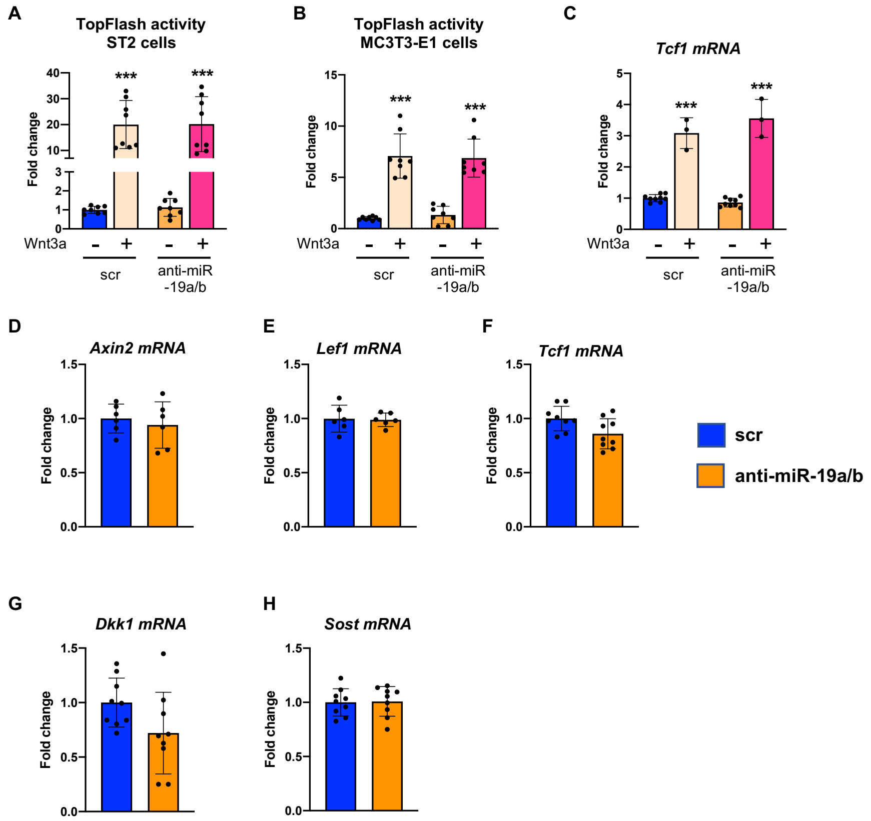

- Is Tgif-1 expression also altered by sex-steroid deficiency?

We thank the reviewer for this very good question. Given the relevance of this point, we extended the experiments and determined the expression of miR-19a/b, PP2A-Cβ and Rankl in this context as well, in addition to Tgif1.

In vivo, ovariectomy-induced sex steroid-deficiency increases the expression of endogenous miR-19a and miR-19b, while a strong trend towards a decrease in Tgif1 expression was determined compared to sham-operated control animals (Fig. 7 A-C). *In vitro*, estrogen deficiency significantly increased miR-19a, miR-19b and PP2A-Cβ expression, while the expression of Tgif1 was significantly reduced (Fig. 7 D-G). Furthermore, estrogen deficiency expectedly increased the expression of Rankl, while anti-miR19a/b treatment alone had no effect on Rankl expression but reversed the estrogen deficiency-induced increase in Rankl expression (Fig. 7 H). Consistent with these findings, expression of miR-19a, miR-19b and Rankl was increased in an *in vitro* model of androgen deficiency while expression of Tgif1 was significantly reduced (Appendix Figure S6 A-D).

These important findings demonstrate that under pathological conditions of sex steroid-deficiency, endogenous miR-19a/b is increased, leading to a decrease in Tgif1 expression and a consecutive increase in PP2A-Cβ and Rankl expression. This contributes to a decrease in bone formation and an increase in bone resorption and therefore to a pathological loss of bone mass. Therapeutic interference with anti-miR19a/b reverses this condition, leading to an increase in bone mass and structure. These new insights have been integrated in the revised manuscript and summarized in a schematic model (Fig. 7 P).

Discussion:
The authors should further discuss the different effects of anti-miR-10a/b treatment on osteoclasts if given alone or in combination with PTH or after OVX.

We agree with the reviewer that discussing these findings in the context of normal- and high bone remodeling, in particular in light of the new data on the underlying molecular mechanisms, is very important for the reader. We therefore discuss these aspects in the revised manuscript (page 21, line 14-23 and page 22, line 1-3).

Methods
Detailed description would allow repetition of the work.

We added more experimental details to the methods section and throughout the manuscript. Furthermore, we included detailed additional information related to experiments conducted during revision. The more detailed methods part should allow others to repeat the work.

Referee #2 (Remarks for Author):
The manuscript of Taipaleemäki et al. shows that skeletal expression of miR-19a/b is reduced by treatment with PTH or anti-sclerostin antibody. Moreover, using cell culture and animal models, they identify TGIF as a downstream mediator of miR-19a/b that mediates a bone-anabolic response via increasing bone formation. Finally, they show that antagonizing miR-19a/b enhances the bone anabolic effect of PTH and enhances bone mass in models of sex hormone deficiency, thus, proposing it as a novel treatment strategy for osteoporosis. Overall,

this is an easy to follow manuscript with a logical structure, sufficient methodological details to replicate the study, and sound results that are presented in a comprehendible way. The topic of finding new drugs for osteoporosis treatment is of great interest.

We thank the reviewer for emphasizing the quality and comprehensiveness of the data and the overall relevance of the research topic. The manuscript is perceived by this reviewer as logic and well-structured with sufficient methodological details. We appreciate the very good comments and input provided by this reviewer and think that we addressed all points fully.

Major comments:

1. The rescue of the bone phenotype induced by miR-19a/b knockdown via TGIF deficiency in osteogenic cells is quite intriguing. TGIF seems to play no role on the increase in bone formation parameters in mice with a miR-19a/b knockdown. However, bone resorption parameters are higher, if TGIF in Dmp1-expressing cells is not present. Thus, given that TGIF is only deleted in osteogenic cells, it seems these cells exert stimulatory signals to osteoclasts. Have the authors checked RANKL/OPG and does TGIF in osteogenic cells alter their communication to osteoclasts? Would co-cultures be useful to address this communication? Is miR-19a/b expressed in osteoclasts?

We appreciate this very good comment made by the reviewer and for supporting the importance of the experiment demonstrating the prevention of an increase in bone mass by Tgif1-defiency in osteoblast lineage cells in response to anti-miR-19a/b treatment. In the revised manuscript, we present a complete elucidation of the molecular mechanisms by which anti-miR-19a/b acts anti-resorptive, particularly under conditions of high bone turnover.

Although miR-19a/b is expressed in osteoclasts (Fig. 6 A, B), it is dispensable for osteoclast differentiation (Fig. 6 C, D), indicating that anti-miR-19a/b suppresses bone resorption in an indirect manner. After exclusion of several soluble factors that could mediate this effect, we uncovered that anti-miR-19a/b suppresses the increase in Rankl expression (e.g. in response to PTH treatment or ovariectomy) in a Tgif1-PP2A-Cβ-Crct2-dependent manner in osteoblast lineage cells (Fig. 6 E-M, Appendix Figure S6 A-D).

According to this model integrating these novel findings (Fig. 7 P), in the absence of Tgif1 in cells of the osteoblast lineage the bone-forming activity of osteoblasts is modestly impaired in response to anti-miR-19a/b treatment while a basal repression of Rankl expression is attenuated. This explains why some osteoclast-related parameters are slightly elevated while the increase of some osteoblast-related parameters is modestly compromised in *Dmp1-Cre^{Tg};Tgif1$^{fl/fl}$* mice treated with anti-miR-19a/b compared to control littermates (Fig. 3 D). Ultimately, this disturbed bone remodeling prevents the increase in bone mass in a Tgif1-dependent manner (Fig. 3 C-F).

2. Along those lines, how is the modulation of Wnt signaling in these cells (cells without TGIF and with anti-miR-19a/b)? This phenotype resembles lack of Wnt signaling in osteogenic cells and the abnormal crosstalk to osteoclasts. This is also displayed again by the actions shown in Suppl. Fig. 8, which reminds one more of the effects of anti-sclerostin therapy than PTH (due to the uncoupled effect on bone remodeling). Does Sost (or Dkk1 or another Wnt inhibitor) play a role in mediating the effects of miR-19a/b?

We agree with the reviewer that the effects of anti-miR-19a/b treatment have some similarities with the ones seen by Wnt activation. However, a great difference, particularly under conditions of high bone turnover for instance in response to ovariectomy-induced sex steroid-deficiency,

is that pharmacological activation of Wnt *in vivo* (e.g. by Scl-antibody) causes a strong anabolic effect that ceases over time combined with an anti-resorptive component. In contrast, anti-miR-19a/b acts anti-resorptive with an anabolic component under conditions of high bone turnover. The gain in bone mass in response to anti-miR-19a/b treatment is therefore a consequence of dual mode of action comprising the suppression of a pathologically increased Rankl expression and consecutive bone resorption combined with an anabolic component. In the context of normal Rankl expression, for instance under steady-state conditions, anti-miR-19a/b functions solely anabolic by stimulation of osteoblast-dependent bone formation.

To address the well taken point of the reviewer, we investigated a potential effect of anti-miR-19a/b on the activity of canonical Wnt signaling by conducting a set of *in vitro* experiments. We transfected cells of the osteoblast-like cell lines ST2 and MC3T3-E1 with anti-miR19a/b or scrambled control as well as with the TopFlash reporter gene construct and stimulated these cells with Wnt3a or vehicle. However, we did not observe any effect of miR-19a/b inhibition on reporter gene activity (see data below, panel A, B). In addition, quantification of the expression of the Wnt target gene Tcf1 in MC3T3-E1 cells did also not reveal any effect of anti-miR-19a/b on Wnt3a-mediated Wnt pathway activation (panel C). Furthermore, inhibition of miR-19a/b did not alter the endogenous expression of the Wnt target genes Axin2, Lef1 or Tcf1 in MC3T3-E1 cells (panel D-F). Finally, expression of the Wnt inhibitors Dkk1 and Sost in UMR-106 cells was also not affected by anti-miR-19a/b treatment (panel G, H). In conclusion, the combined *in vitro* evidence does not suggest any effect of anti-miR-19a/b on Wnt pathway activation. We therefore conclude that the Wnt pathway is not the primary mediator of the effect of anti-miR-19a/b on bone remodeling. The findings are presented in this letter but not in the manuscript. However, the data can be shown if requested by the reviewer.

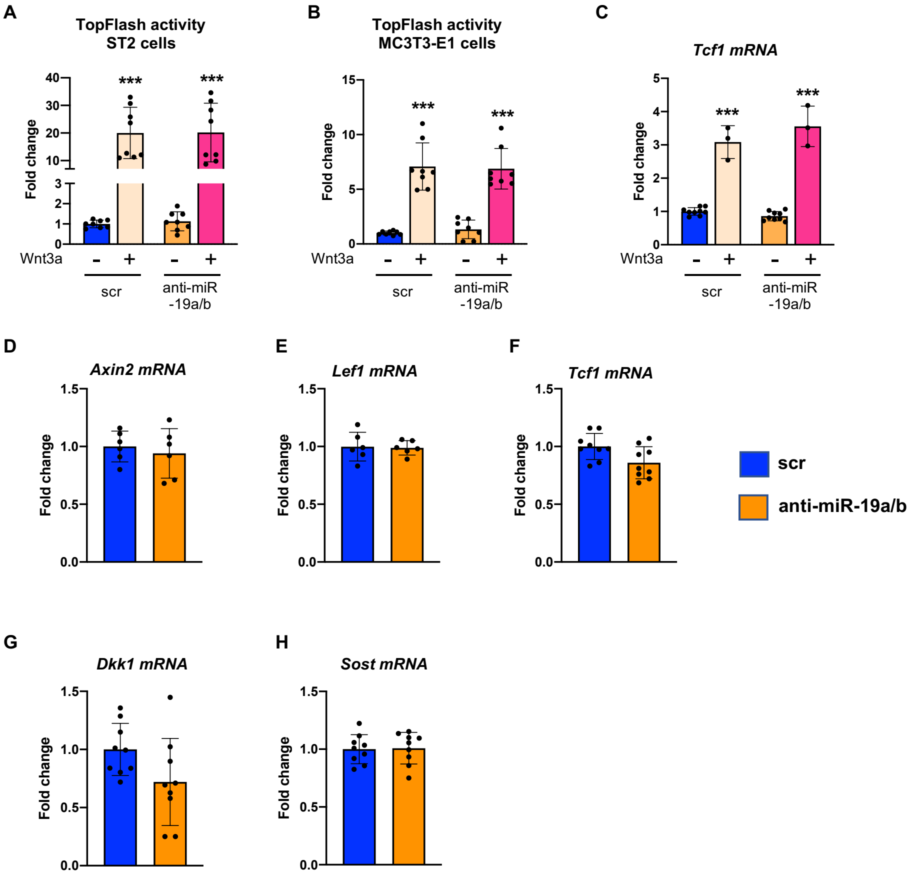

3. Overall, it appears to this reviewer, as if most of the effects of antagonizing miR-19a/b (after PTH treatment and OVX/ORX, for some reason not in steady-state) does not stem from the increase in bone formation, but from the indirect effects on suppressing bone resorption. I think this should be stressed more throughout the manuscript. I think this is also the reason why the treatment works so nicely in OVX and ORX, which are mostly osteoclast driven. This goes in line with the abstract and introduction, which I would rather not stress on age-related osteoporosis, but post-menopausal osteoporosis, as these are also the models the authors use later on. Also, in some figures, it may be more useful to show one bone site and the histology data next to them, than burry them in the supplementary figures. This would immediately show the cellular mechanisms of action without needing to go to the supplement.

We appreciate that the reviewer raised this very important point. It is indeed true that under conditions of an activated bone remodeling and increased bone resorption such as in the context of PTH treatment or sex steroid-deficiency in response to OVX/ORX the predominant effect of anti-miR-19a/b treatment is the reduction of bone resorption. As rightly mentioned by the reviewer, this is not the prevailing mechanisms in healthy mice under steady state conditions. Here, the prevailing effect of anti-miR-19a/b treatment is the increase in bone formation.

During revision, we conducted a complete set of experiments and unraveled the mechanisms explaining this mode of action. Basically, under conditions of high bone turnover, expression of Rankl is increased, which stimulates osteoclast differentiation, function, and bone resorption. In the framework of this catabolic signaling, anti-miR-19a/b antagonizes the elevated abundance of endogenous miR-19a and miR-19b, causing an increase in Tgif1 expression. Tgif1 inhibits PP2A-Cβ, leading to a reduced nuclear translocation of Crct2 and subsequent Rankl expression (Fig. 6 I-M). By this mechanisms, anti-miR-19a/b acts primarily anti-resorptive while the positive effect on bone formation is still present (Fig. 4 C, E and Fig. 5H). If Rankl expression is normal, such as under physiological conditions, anti-miR-19a/b acts primarily in an anabolic manner (Fig. 1 H, I). All new data have been incorporated in the revised manuscript.

We agree with the reviewer that the terms "post-menopausal osteoporosis" or "sex steroid deficiency-induced osteoporosis" are more suitable than "age-related osteoporosis". We made changes accordingly throughout the manuscript. In addition, we followed the recommendation of the reviewer and present the histology and the corresponding data next to each other in the main figures to better demonstrate the cellular mechanisms (Fig. 3 C, D and Fig. 4 B-E and Fig. 5 B-H).

4. Synergism of anti-miR-19a/b treatment and PTH is more pronounced at the tibia. No additive effects are observed in the spine. Do the authors have an explanation for this? Is the expression of miR-19 different? Usually, PTH has more pronounced effects on the spine than the hip/femur. How are these different effects between miR-19a/b and PTH reconciled?

We agree with the reviewer that this observation might appear counter intuitive because as rightly mentioned, in humans the anabolic effect of PTH treatment is more pronounced at the lumbar spine than in long bones. Expression of miR-19a/b does not differ between skeletal sites and can therefore not explain this effect. However, it has been reported that in mice, other than in humans, the PTH-stimulated increase in trabecular area and BMD is more prominent in the tibia and femur than in the lumbar spine (*Iida-Klein et al., J Bone Miner Res., 2002*). These findings demonstrate that the activation of bone-remodeling in the spine due to PTH treatment varies between species with a lower response in mice compared to humans. Our data are consistent with this report. To share this insight with the readers, we discuss this observation in the revised manuscript (page 21, line 9-13).

5. The patient cohort should be better described. Besides being old and having OA, what other diseases/medications were present?

We agree with the reviewer that providing more information on the characterization of the patient cohort is useful to the readers. Indeed, we performed a comprehensive screening prior to including patients in this study. In addition to obtaining a medical history and drug history, we analyzed a large number of laboratory parameters that altogether allowed us to exclude patients that have metabolically- or drug-induced alterations of bone metabolism. Only patients that did not take drugs affecting bone metabolism and did not present laboratory abnormalities were included. A list of parameters analyzed is provided in the revised manuscript (page 32, line 5-16).

<u>*Minor comments:*</u>

6. Can the authors explain/comment on the differences between miR-19a and b? The expression and regulation seems to overlap to a significant amount. Is this expected or unusual?

It is expected that the expression and regulation of miR-19a and miR-19b is highly similar since both miRNAs arise from the miR-17-92 cluster and are expressed from the same promoter. The sequence of miR-19a and miR-19b differs only in one nucleotide at position 11, which is outside the seed sequence. Since the seed sequence, which is critical for recognition and binding to the 3'UTR of the target mRNA is identical between miR-19a and miR-19b, both miRNAs have identical downstream targets.

7. It is not entirely clear why housekeeping genes were used to look at potential adverse effects on different organs.

Housekeeping genes are implicated in the regulation of basic cellular functions that ensure cell differentiation and survival. Compounds that impair cellular functions in a cytotoxic manner can affect the expression of housekeeping genes. Quantifying a panel of 4 different housekeeping genes in different organs allowed us to detect putative toxic effects at the cellular/tissue level. Since no changes in the expression of any housekeeping gene was observed in response to anti-miR-19a/b treatment, we concluded that no major adverse effects occurred. This was further supported by analyzing organ histology *ex vivo,* weight and behavior *in vivo* as well as cell viability and apoptosis *in vitro*.

8. The normalization in some figures including Fig. 2B/C makes it difficult to appreciate the level of miR-19a/b expression in the different tissues. It would be more informative to see the absolute values to get an impression of how much miR-19 is expressed in the various tissues.

To provide information on the expression of miR-19a and miR-19b within a given tissue, we normalized the expression of miR-19a and miR-19b only to the expression of the housekeeping gene U6 as internal control (ΔCT). This provides information on the absolute expression in various tissues and allows comparison between different tissues. Data are presented in the Fig EV1 D, E, and the mode of normalization has been indicated in the graphs, figure legends and on page 25, line 22-23).

9. Full Western blots should be provided as supplementary figures. Western blots should be semi-quantified.

Immunoblots have been semi-quantified and the data are presented next to each blot. Quantification of band intensities was performed by grey scale analysis using Image Lab Software (Biorad). The intensity of the signal of interest was normalized to the intensity of the internal control signal (Actin or Lamin A/C). Normalized ratios were quantified and are expressed as fold change relative to the experimental control group (page 29, line 12-15). Full immunoblots have been deposited in the figure source data file.

10. Paragraph two of the discussion could be shortened.

We shortened this paragraph in the revised manuscript.

11. Two-way ANOVA should be used for Fig. 3C-F and Fig. 4B-E.
We thank the reviewer for this valuable comment regarding our statistical analysis. To address this point, we have consulted Dr. Helmut Küchenhoff, Professor and Director of the Statistical Consulting Unit, Department of Statistics at LMU Munich. According to the suggestion of the reviewer, we re-analyzed the data in Figure 4 C, E using two-way ANOVA. As a result, some significant values changed but not in a way that this would affect the interpretation of the data. However, according to the recommendation given by our statistician, we re-analyzed the data in Figure 3 D, F using Kruskal-Wallis test, a non-parametric test, which does not assume a normal distribution of the residuals. Re-analysis of these data resulted in some changes in the significance values that further support our hypothesis on the importance of Tgif1 in osteoblasts for the function of anti-miR-19a/b. Briefly, based on the new analysis, anti-miR-19a/b is not able to fully activate the osteoblasts (Ob.S/BS, N.Ob/BS) in the absence of Tgif1 in Dmp1-expressing cells. As a result, anti-miR-19a/b treatment increases bone formation, mineral apposition rate, mineralized surface and osteoid surface less in the absence of Tgif1 in osteoblasts compared to littermate controls. These results are integrated in the revised manuscript on page 10, lines 6-9. The updated statistical analysis is reported in the methods section (page 32, lines 16-21) and figure legends of the revised manuscript.

12. It could be explained a little bit better how TGIF was chosen to be further investigated as a downstream target of miR-19a/b. I am aware that the authors have studied TGIF a lot, but for sure other targets of miR-19a/b are available, perhaps including components of the Wnt signaling pathway?

To identify targets of miR-19a/b we applied several algorithms including TargetScan, PicTar and miRWalk. Using this *in silico* approach, we did not identify components of the Wnt signaling pathway. However, among the predicted targets we identified Tgif1, which was highly ranked by all algorithms and novel, since it had not been previously identified and validated as a miR-19a/b target. Given the unbiased evidence, novelty and the resources we had in our group, we decided to further explore the Tgif1 mechanistic pathway. This information has been included in the revised manuscript on page 9, line 10-13.

Referee #3 (Comments on Novelty/Model System for Author):
At this stage, the paper presents insufficient detail on the methods used to be confident about the data presented. If they are able to address these concerns, the model and methods of measurement may prove to be adequate.

Referee #3 (Remarks for Author):
This work from Taipaleenmaki provides interesting new data to show that inhibition of miR-19a/b may provide a novel pathway to promote bone formation which may provide effects additional to PTH-based anabolic therapy. The introduction is very clearly written. Generally, the data is well presented, but the methods are poorly described, and some model systems not clearly defined. In some cases, it is difficult to be confident of the conclusions drawn without this additional information.

We thank the reviewer for considering our findings interesting and novel. We appreciate acknowledging that the paper is well written, and that the data are presented in a clear manner. In the revised manuscript we describe the methods and models in greater detail and provide further information as suggested. All other points raised have also been answered in detail.

Major concerns:
1. Method details are lacking for many of the experiments, which makes it difficult to judge the validity of the data. Some examples: (1) the vehicle used, and the time point at which vehicle samples were collected for the data shown in Figure 1A, (2) the bioinformatic analysis used for the data shown in Figure 1A, (3) regions used for histomorphometry and micro-computed tomography, (4) thresholding levels used for micro-computed tomography, (5) sex of mice used for the experiment described on p7 (Figure 1), (6) details of in silico analysis described on p9, line 9, (7) age and sex of Dmp1-Cre<Tg>Tgif mice and duration of treatment used for data shown in Figure 3, (8) sex and age of mice for PTH + anti-miR studies shown in Figure 4 (9)

We appreciate the detailed comments by the reviewer and apologize for lacking some experimental information, which we are presenting in the revised manuscript. Below we provide information to the specific points raised by the reviewer and integrated overall more experimental information throughout the revised manuscript.

1. For the experiment presented in Figure 1A, we used a control vehicle composed of 0.1mM acetic acid, 0.01% bovine serum albumin (BSA) in H_2O (page 24, line 3). For both groups (PTH and Scl-Ab) we used separate vehicle groups, which were collected at the same time point as the treatment (vehicle for the PTH group after 4h, vehicle for the Scle-Ab group after 24 hour) (page 24, line 4).
2. RNAseq analysis and calculation of significance was performed at Exiqon (Vedbaek, Denmark). Values derived from vehicle groups were combined mathematically (page 24, line 6-8).
3. For analysis by histomorphometry and μCT, a region starting 200μm below the growth plate of the long bones was used. For histomorphometry, the average size of the region of interest was 2.5 mm^2 (page 27, line 14-16).
4. The threshold value used in all μCT analyses was determined at 326 mg/cm^3 hydroxyapatite based on Hounsfield units and a phantom with a linear hydroxyapatite gradient (79 – 729 mg/cm^3) (page 27, line 11-13).

5. Healthy 8-week-old male C57Bl/6J mice were used for this experiment (page 7, line 6 and figure legend)
6. *In silico* analysis was performed using TargetScan, miRWalk and PicTar algorithms for the identification of miR-19a/b targets. The significant top hundred conserved targets revealed by each algorithm were used to identify commonly predicted targets. These targets were ranked according to significance and novelty, leading to the identification of the homeodomain protein TG-interacting factor 1 (Tgif1) (page 9, line 10-13).
7. For the targeted deletion of Tgif1 in the osteoblast lineage as shown in Figure 3, we used 8-week-old male mice and a treatment period of four weeks. Bones of mice were therefor analyzed at an age of 12 weeks (page 10, line 5; page 26, line 14 and figure legend).
8. For anabolic studies using anti-miR-19a/b and PTH treatment (Figure 4), 8-week-old male C57Bl/6 wild type mice were treated for four weeks and analyzed at an age of 12 weeks (page 10, line 19; page 26, line 12 and figure legend).

2. Please provide evidence that anti-miR19a, anti-miR19b, and anti-miR19a/b were successful at achieving knockdown in the cell culture experiment shown in Figure 1D, and provide quantitation of the alkaline phosphatase assay. A single image is not sufficient evidence of altered osteoblast activity. Ideally mRNA markers of osteoblast differentiation should also be reported for this experiment. Without this, the authors cannot really claim that "differentiation was enhanced".

As suggested by the reviewer, we quantified the decrease of endogenous miR-19a/b expression in osteoblasts cultured *in vitro* in response to anti-miR treatment (Fig. EV 1 B, C). In addition, alkaline phosphatase (ALP) activity was quantified in *in vitro* cultured osteoblast (Fig. 1 E). Furthermore, as suggested, we determined the expression of Runx2 and Col1a1 as markers of osteoblast differentiation (Fig. 1 F, G). Successful reduction of miR-19 expression combined with an increase in ALP activity and a higher expression of markers of osteoblast differentiation support our hypothesis that anti-miR-19a/b treatment enhances osteoblast differentiation.

3. The evidence that Tgif is required for the anabolic action of miR-19a/b blockade is not very convincing and does not add to the story. This data could be removed and the paper would be improved. The substantial increase in osteoblast markers in response to miR-19a/b blockade in these mice, and the data showing a lack of increase in BV/TV is not normally distributed. The evidence is very poor, and should be removed.

We thank the reviewer for this comment. However, in our view this experiment reports an important *in vivo* mechanistic finding as it demonstrates that Tgif1 is a critical downstream target of miR-19a/b. This experiment therefore provides valuable *in vivo* genetic-based insights that an increase in bone mass in long bones and vertebra by anti-miR-19a/b largely depends on Tgif1 in cells of the osteoblast lineage. We respectfully decided to leave the findings in the manuscript.

We appreciate the expert comment of the reviewer on the statistical analysis. To ensure correct statistical analysis of our data we have consulted Dr. Helmut Küchenhoff, Professor and Director of the Statistical Consulting Unit, Department of Statistics at LMU Munich. According to our statistician, sample size should be at least 30 to statistically test normal distribution of data in a reliable way. However, as the reviewer pointed out, based on the distribution of the data presented in Fig. 3 E, F normality should not be assumed. Therefore, based on the statistician's advice, we have re-analyzed the data using Kruskal-Wallis test, a non-parametric

tests, which does not assume a normal distribution of the residuals. Re-analysis of these data resulted in changes in the significance values that further support our hypothesis on the importance of Tgif1 in osteoblasts for the function of anti-miR-19a/b. Briefly, based on the re-analysis, anti-miR-19a/b is not able to fully activate the osteoblasts (Ob.S/BS, N.Ob/BS) in the absence of Tgif1 in Dmp1-expressing cells. As a result, anti-miR-19a/b treatment increases bone formation, mineral apposition rate, mineralized surface, and osteoid surface less in the absence of Tgif1 in osteoblasts compared to littermate controls. These results are integrated in the revised manuscript on page 10, lines 6-9. The updated statistical analysis is reported in the methods section (page 33, lines 15-16) and figure legends of the revised manuscript.

4. Throughout the study, it seems that all of the mice used are young, growing animals. These are not a good model of bone remodeling, or a good model of adult osteoporosis. This is a major limitation which should be discussed in detail in the paper.

In this study, female or male mice of C57BL/6J background were 8-week-old when anti-miR-19a/b or PTH treatments were initiated and 12-week-old by the time of analysis. OVX and ORX were also performed at 8-week-of age. In the OVX experiment, mice had an age of 18 weeks and in the ORX model, mice were 19-week-old by the time of analyzing the bone phenotype. Since longitudinal growth of mice is usually completed before the age of 8 weeks, we do not consider these mice as growing anymore. Furthermore, mice of this age are often used in studies investigating bone remodeling also in the context of ovariectomy-induced osteoporosis and PTH treatment (*e.g., Arlt H. et al., Bone Rep, 2020; Dohke T. et al., J Bone Miner Metab., 2018; Wu X. et al., Calcif Tissue Int, 2017*). In addition, a recent study presented a detailed investigation of the effect of ovariectomy on bone density and microarchitecture in female C57BL/6J mice at 8-, 12-, and 16-weeks of age. The group also compared C57BL/6J mice with mouse strains of other genetic backgrounds. The data demonstrate that bone loss is the greatest in 8-week-old animals while it is weak in 16-week-old mice compared to sham control animals (*Zhou S., et al. Exp Ther Med, 2018*). Compared with other genetic backgrounds, bone loss was the greatest in C57BL/6J mice. Based on these findings the authors concluded that "8-week-old female C57Bl/6J mice are a good animal model for postmenopausal osteoporosis and preclinical testing of potential therapies for this disease" (*Zhou S., et al. Exp Ther Med, 2018*). We therefore think that it is appropriate to investigate skeletally mature mice of the indicated ages to address the questions raised in this manuscript as a widely accepted model in the field. However, as suggested by the reviewer, we are discussing this aspect in the revised manuscript (page 21, line 14-16).

5. The patient data is very poorly described, and based on extremely low numbers. I recommend removing it. It adds little to the work. Without explaining the tissue collected, and showing the micro-CT data, it is not very strong evidence at all, and raises more questions than it answers.

We understand the concerns of the reviewer but believe that confirming our findings in human material provides important impact to the manuscript. However, to address the points raised, in the revised manuscript we describe how carefully we characterized the patient cohort prior to including patients in this study. In addition to obtaining a medical history and drug history, we analyzed a large number of laboratory parameters that altogether allowed us to exclude patients that have metabolically- or drug-induced alterations of bone metabolism. Only patients that did not take drugs affecting bone metabolism and did not present laboratory abnormalities were included. Parameters analyzed are indicated in the revised manuscript (page 32, line 7-16). We

agree with the reviewer that it is useful to show μCT data. We therefore included μCT images and the quantification of bone mineral density (BMD) in the revised manuscript and present these data together with histological images and histomorphometric parameters (Fig. 7 I-L).

6. The authors repeatedly use the term "osteoporosis" to describe low bone mass in mice. This is inaccurate, and should be changed. Mice do not suffer from osteoporosis; it is more accurate to say "low bone mass", or even "gonadectomy-induced bone loss".

We accept the point made by the reviewer and clarified throughout the revised manuscript that gonadectomy was performed to induce sex steroid-deficiency. Nevertheless, this condition resembles postmenopausal osteoporosis and is an accepted animal model to study this disease. These changes were also made in alignment with the comment #3 given by referee #2.

7. It is surprising that miR-19a/b inhibition and PTH have additive effects when PTH itself already reduces miR-19a/b. This suggests they may have different modes of action, and that PTH anabolic effect is not dependent on miR-10a/b. Some discussion of this would be appropriate.

We thank the reviewer for this valuable comment. In our view it is plausible that anti-miR-19a/b treatment has synergistic effects with PTH treatment. Decreasing endogenous miR-19a/b expression is part of the molecular mechanism of PTH signaling. Thus, endogenous miR-19a/b is reduced to participate in the full gain in bone mass by PTH. Concomitant pharmacological inhibition of endogenous miR-19a/b by anti-miR-19a/b is further supporting this effect, thereby augmenting the effect of PTH treatment. These findings implemented in the larger context of the manuscript suggest to us that miR-19a/b is an important downstream component of the PTH signaling cascade. However, this does not exclude the possibility that PTH has miR-19a/b-independent effects and that anti-miR-19a/b treatment targets pathway components that are independent of PTH signaling. We therefore appreciate the recommendation of the reviewer and discuss this aspect in the revised manuscript (page 20, line 21-page 21, line 2).

<u>*Additional comments:*</u>

1. p3, line 18. Abaloparatide is not the same as the first 34 amino acids of PTHrP; it is a modified form of the 34 amino acid protein.

The reviewer is right. We corrected this inaccuracy (page 3, line 20).

2. Figure 1A, most of the regions of the Venn diagram contain no information. Is that because no unique genes were regulated by veh, PTH and Scl-Ab?

In total, 142 miRNAs were identified to be regulated in this experiment by whole-genome miRNA sequencing. Out of this, 107 miRNAs were only regulated by Scl-Ab treatment compared to vehicle control and 13 miRNAs were only regulated by PTH compared to vehicle control. 22 miRNAs were regulated by PTH and Scl-Ab treatment. The number of the miRNAs are written in the space where the circles indicate treatment overlap (Fig. 1 A).

3. ObS/BS is not a marker of osteoclast size. Please rephrase (p11, line 6).

The manuscript mentions that the size of osteoclasts is reflected by the histomorphometric parameter Oc.S/BS (page 11, line 6-7).

4. The authors state that anti-miR treatment "reduced activated bone resorption" but present no evidence to support this (p11, line 12).

Intermittent administration of PTH 1-34 is known to activate bone remodeling, which includes an increase in bone resorption. This is confirmed in our experiment demonstrating an increase in histomorphometric parameters of osteoclast-dependent bone resorption at the proximal tibia and the lumbar spine (ES/BS, Oc.S/BS, N.Oc/BS) (Fig. 4 C, E). These indices reflecting an activated bone resorption are reduced by co-treatment with anti-miR-19a/b (Fig. 4 C, E). Furthermore, quantification of CTX as serum marker of bone resorption confirmed that PTH-mediated increase in bone resorption is indeed attenuated by anti-miR-19a/b (Fig. 4 F). Similar findings were obtained in ovariectomy-induced high turnover bone loss at the tissue, cellular and gene expression level (Fig. 5 H-J). Collectively, these findings confirm that anti-miR-19a/b treatment indeed reduces an activated bone resorption.

5. It would help the reader if the methods could be reported in the same order as the experimental data shown.

As requested by the reviewer, we have changed the order of the experimental procedures to follow the data presentation in the figures as much as possible. However, this is not absolute strict since the same methods are used in different experiments.

6. Please describe whether bone samples were flushed of marrow before mRNA was generated.

Yes, bones were flushed of bone marrow prior extracting mRNA from bone envelopes. We specify this in the revised methods section (page 24, line 5).

20th Jun 2022

Dear Prof. Hesse,

Thank you for the submission of your revised manuscript to EMBO Molecular Medicine. We have now heard back from the three referees who we asked to re-evaluate your manuscript. As you will see from the reports below, while the referees #1 and #2 are supporting publication of your work, referee #3 raises important concerns that should be addressed in an additional and final round of major revision. Particular attention should be given to the interpretation of the data and the proper use and report of statistical tests. Limitations of the mouse model should be discussed, no additional experiments with older mice are required.

Further consideration of a revision that addresses reviewer's concerns in full will entail an additional round of review. Acceptance or rejection of the manuscript will depend on the completeness of your responses included in the next, final version of the manuscript. For this reason, and to save you from any frustrations in the end, I would strongly advise against returning an incomplete revision.

We would welcome the submission of a revised version within three months for further consideration. Please let us know if you require longer to complete the revision.

Please use this link to login to the manuscript system and submit your revision: https://embomolmed.msubmit.net/cgi-bin/main.plex

I look forward to receiving your revised manuscript.

Yours sincerely,

Zeljko Durdevic

Zeljko Durdevic
Editor
EMBO Molecular Medicine

***** Reviewer's comments *****

Referee #1 (Comments on Novelty/Model System for Author):

The author described in their point-to-point reply in great detail, why the used mouse line is appropriate.

Referee #1 (Remarks for Author):

I want to congratulate the authors on this new version of the manuscript. The new data and text clearly increase the value of this study and improved the manuscript greatly. The authors addressed all of my previous concerns. Especially the data regarding RANKL is very interesting.

Referee #2 (Comments on Novelty/Model System for Author):

The authors have added several more data in particular on the crosstalk between osteoblasts and osteoclasts and in my opinion, have substantially improved the manuscript.

Referee #2 (Remarks for Author):

The authors have added several more data in particular on the crosstalk between osteoblasts and osteoclasts and in my opinion, have substantially improved the manuscript.

Referee #3 (Comments on Novelty/Model System for Author):

The conclusions are not supported by the data presented. Two-way ANOVAs are not appropriately reported, and a very young mouse model has been used as a model for postmenopausal osteoporosis. I have made suggestions about how to deal with these two major problems in my comments to authors. However, since both of these issues were raised in the first submission, I have little confidence that the authors will address them.

Referee #3 (Remarks for Author):

The authors have improved the methods section of the paper, making it now possible to assess the validity of the data. As it currently stands, the data presented, while novel, does not support the conclusions made. This paper does not provide convincing evidence that (1) anti-miR-19a/b effects are mediated by Tgif, (2) that PTH anabolic actions are modified by anti-miR-19a/b, and provides limited evidence that anti-miR-19a/b could provide benefit for postmenopausal osteoporosis.

Remaining major concerns:

1. I continue to disagree very strongly with the terms "osteoporotic mice" and "mouse models of osteoporosis". The use of 8 week old ovariectomised mice is not a model for postmenopausal osteopenia, regardless of how old the mice are at the time of tissue collection. Transverse and longitudinal growth, both continue after 8 weeks of age, and the trabecular network is not yet established at this time (see Glatt et al, JBMR 2009; doi.org/10.1359/jbmr.070507). The effect of ovariectomy at this time point, while substantial, is an effect that is interruption of full pubertal development, and an effect primarily on generation of new trabeculae rather than an effect on bone remodelling. The claim that that this is an "established model" of postmenopausal

osteoporosis is disingenuous and harmful. I want to emphasise that the use of young animals does not detract from the novelty of the data - but the authors should not overstate the value of the mouse model they have used. While the work will require repeating in an aged OVX model in the future, for the purposes of this paper it is important not to overclaim the importance of their findings.

2. Figure 2A: The evidence that administration of anti-miR-19a/b does not cause an effect on bone marrow is based on a single von Kossa image, but the statement is made that "no tissue abnormalities in bone, bone marrow... were detected". Why is such a small region of bone shown here, rather than a full tibia as in the other figures? Ther is no quantitation. This is a very bold statement with very little evidence. Are these the same animals shown in Figure 1? What about bone length, bone width, cortical thickness?

3. Figure 3C-F: The authors claim that the effect of anti-miR-19a/b is dependent on Tfig1 expression, but this is not supported by the data presented. They see a significant increase in MS/BS, N.Ob/BS, BFR/BS and MAR, but show only BV/TV (without Tb.N., Tb.Th.). They state that the effect on bone formation related parameters is "less pronounced", but do not show p values from their new two-way ANOVA (suggested by another reviewer) to show that the interaction between genotype and anti-miR treatment is significant. This is essential - if the interaction is not significant, there is no evidence is presented that the effect of miR-10a/b is less in the Tgif deficient mice.

4. Figure 4: Similarly, the authors claim this provides evidence that anti-miR-19a/b augments the anabolic effect of PTH, but the data presented does not include results of the two-way ANOVA interaction (again requested by another reviewer). Is the interaction between PTH and anti-miR treatment significant. The data appears to show that PTH treatment in both the presence and absence of Tgif knockdown is effective in both tibiae and vertebrae.

Minor concerns:
1. Introduction: line 12, the statement that "In the context of sex steroid-deficiency such as after menopause, bone resorption often increases while bone formation decreases" requires a supporting reference.
2. The authors state that ObS/BS and OcS/BS are markers of osteoblast and osteoclast size, respectively. They are not measurements of cell size. They indicate the extent of bone surface containing osteoblasts or osteoclasts. This can be influenced by both cell size and by cell number (p7, lines 16-18, p11, line 13).

<u>**Response to reviewers**</u>

We are grateful to all referees for their comments on our revised manuscript and are delighted that referees #1 and #2 give clearance to publication. In addition, we did our best to fully address the remaining points of referee #3 in a reasonable manner as described below. We believe that the manuscript is now acceptable for publication.

Referee #3 (Comments on Novelty/Model System for Author):

The conclusions are not supported by the data presented. Two-way ANOVAs are not appropriately reported, and a very young mouse model has been used as a model for postmenopausal osteoporosis. I have made suggestions about how to deal with these two major problems in my comments to authors. However, since both of these issues were raised in the first submission, I have little confidence that the authors will address them.

Referee #3 (Remarks for Author):

The authors have improved the methods section of the paper, making it now possible to assess the validity of the data. As it currently stands, the data presented, while novel, does not support the conclusions made. This paper does not provide convincing evidence that (1) anti-miR-19a/b effects are mediated by Tgif, (2) that PTH anabolic actions are modified by anti-miR-19a/b, and provides limited evidence that anti-miR-19a/b could provide benefit for postmenopausal osteoporosis.

Remaining major concerns:

1. I continue to disagree very strongly with the terms "osteoporotic mice" and "mouse models of osteoporosis". The use of 8 week old ovariectomised mice is not a model for postmenopausal osteopenia, regardless of how old the mice are at the time of tissue collection. Transverse and longitudinal growth, both continue after 8 weeks of age, and the trabecular network is not yet established at this time (see Glatt et al, JBMR 2009; doi.org/10.1359/jbmr.070507). The effect of ovariectomy at this time point, while substantial, is an effect that is interruption of full pubertal development, and an effect primarily on generation of new trabeculae rather than an effect on bone remodelling. The claim that that this is an "established model" of postmenopausal osteoporosis is disingenuous and harmful. I want to emphasise that the use of young animals does not detract from the novelty of the data - but the authors should not overstate the value of the mouse model they have used. While the work will require repeating in an aged OVX model in the future, for the purposes of this paper it is important not to overclaim the importance of their findings.

We follow the recommendation of this referee and agree that this animal model can be conducted with older mice. Nevertheless, using mice of the age we used in this study has been reported in many publications and is therefore also an accepted experimental model of sex steroid deficiency-induced osteoporosis. However, since we accept the valid point of this referee, we mention in the revised discussion that our models have limitations due to the relatively young age of the mice used and that future investigations using aged mice are likely to provide additional valuable information (page 22, lines 18-20). Furthermore, to not overstate the usefulness of our animal model, we removed the statement that “8-week-old female C57Bl/6J mice were used as an established animal model for postmenopausal osteoporosis and preclinical testing of osteoporosis therapies” (page 21, line 15). We hope this brings the model in a balanced perspective and fully addresses the valuable recommendation.

2. Figure 2A: The evidence that administration of anti-miR-19a/b does not cause an effect on bone marrow is based on a single von Kossa image, but the statement is made that "no tissue abnormalities in bone, bone marrow... were detected". Why is such a small region of bone shown here, rather than a full tibia as in the other figures? Ther is no quantitation. This is a very bold statement with very little evidence. Are these the same animals shown in Figure 1? What about bone length, bone width, cortical thickness?

The referee is correct. To better illustrate the absence of tissue abnormalities, Figure 2A shows magnifications of the bone marrow from experimental animals presented in Figure 1, which shows larger sample areas/almost the entire bone. The evidence that administration of anti-miR-19a/b does not cause an effect on bone marrow is not only based on a single von Kossa image. Like for other organ tissues, we determined quantitatively that anti-miR-19a/b treatment does not cause changes in the expression of several housekeeping genes in the bone marrow (Fig. 2G). The statement is therefore based on different and quantitative analyses, which were also done for other organs. We agree with the referee that additional information on bone length and width is of interest and therefore provide additional experimental data and further information in the revised manuscript (page 8, lines 9-10; page 27, lines 16-22 and page 28, lines 1-2). We quantified both parameters using μCT analysis of the femora but did not detect any difference (Fig. 2B, C), thereby excluding negative effects of anti-miR-19a/b on skeletal growth. Information on cortical thickness were already provided and do not show any difference under steady state conditions (Appendix Figure S1).

3. Figure 3C-F: The authors claim that the effect of anti-miR-19a/b is dependent on Tfig1 expression, but this is not supported by the data presented. They see a significant increase in MS/BS, N.Ob/BS, BFR/BS and MAR, but show only BV/TV (without Tb.N., Tb.Th.). They state that the effect on bone formation related parameters is "less pronounced", but do not show p values from their new two-way ANOVA (suggested by another reviewer) to show that the interaction between genotype and anti-miR treatment is significant. This is essential - if the interaction is not significant, there is no evidence is presented that the effect of miR-10a/b is less in the Tgif deficient mice.

This referee indicated during a previous review that the data showing a lack of increase in BV/TV are not normally distributed. To take this aspect into account and to ensure correct statistical analysis of our data, we already consulted Dr. Helmut Küchenhoff, Professor and Director of the Statistical Consulting Unit, Department of Statistics at LMU Munich. Based on the statistician's advice, we had already re-analyzed the data using Kruskal-Wallis test, a non-parametric tests, which does not assume a normal distribution. Two-way ANOVA was not performed. However, to address the current point raised by this referee and to provide further evidence that the effect of anti-miR-19a/b on bone mass is indeed dependent on Tgif1, we extended our statistical assessment by not only comparing to scr control but rather providing a full statistical analysis. The data demonstrate that MS/BS, OS/BS, Ob.S/BS and importantly BV/TV are significantly less increased by anti-miR-19a/b in Tgif1-deficient mice compared to control littermates. We added more information on the rationale of this statistical test to the statistics section (page 34, lines 2-6) and the legend of Figure 3 of the revised manuscript. Data regarding Tb.N. and Tb.Th. had already been provided in the accompanying table (Table EV2) and confirm that these parameters also require the presence of Tgif1 in osteoblasts to be increased by anti-miR-19a/b. Together, our data using appropriate statistical tests confirm that the increase in bone mass by anti-miR-19a/b is indeed largely depended on Tgif1 in osteoblasts.

4. Figure 4: Similarly, the authors claim this provides evidence that anti-miR-19a/b augments the anabolic effect of PTH, but the data presented does not include results of the two-way ANOVA interaction (again requested by another reviewer). Is the interaction between PTH and anti-miR treatment significant. The data appears to show that PTH treatment in both the presence and absence of Tgif knockdown is effective in both tibiae and vertebrae.

We thank the referee for this important comment. Due to the recommendation of our expert statistician, we modified the null hypothesis by not assuming dependency of the parameters/assuming any result (page 10, lines 17-18), which is acceptable in the biological context of this experiment and re-analyzed the data using one-way ANOVA. This revised statistical analysis solidly demonstrates that concomitant treatment with PTH and anti-miR-19a/b has significant additional effects on bone mass and osteoblast/bone formation-related indices over either treatment alone. The information is integrated in the revised Figure 4. Regarding the last point: Data related to PTH treatment in the absence of Tgif1 are not part of this manuscript.

Minor concerns:
1. Introduction: line 12, the statement that "In the context of sex steroid-deficiency such as after menopause, bone resorption often increases while bone formation decreases" requires a supporting reference.

We thank the reviewer for pointing this out. Two supporting references have been added to the revised manuscript (page 3, line 13).

2. The authors state that ObS/BS and OcS/BS are markers of osteoblast and osteoclast size, respectively. They are not measurements of cell size. They indicate the extent of bone surface containing osteoblasts or osteoclasts. This can be influenced by both cell size and by cell number (p7, lines 16-18, p11, line 13).

The reviewer is correct. Corrections have been made accordingly (page 7, lines 15-16 and page 11, line 13).

5th Sep 2022

Dear Prof. Hesse,

Thank you for the submission of your revised manuscript to EMBO Molecular Medicine. I am pleased to inform you that we will be able to accept your manuscript pending the following final amendments:

1) In the main manuscript file, please do the following:
- In M&M, include a statement that informed consent was obtained from all human subjects and that, in addition to the principles set out in the WMA Declaration of Helsinki, the experiments conformed the Department of Health and Human Services Belmont Report.
- In M&M, the statistical paragraph should reflect all information that you have filled in the Authors Checklist, especially regarding randomization, blinding, replication.
- Please be aware that all datasets should be made freely available upon acceptance, without restriction. Use the following format to report the accession number of your data:

The datasets produced in this study are available in the following databases:
[data type]: [full name of the resource] [accession number/identifier] ([doi or URL or identifiers.org/DATABASE:ACCESSION])

Please check "Author Guidelines" for more information.
https://www.embopress.org/page/journal/17574684/authorguide#availabilityofpublishedmaterial
2) The Paper Explained: Please add it to the main manuscript file and rename subtitles to "Problem", "Results", and "Impact".
3) Synopsis:
- Synopsis image: Please provide a striking image or visual abstract as a high-resolution jpeg file 550 px-wide x (250-400)-px high to illustrate your article.
- Please check your synopsis text and image before submission with your revised manuscript. Please be aware that in the proof stage minor corrections only are allowed (e.g., typos).
4) For more information: This space should be used to list relevant web links for further consultation by our readers. Could you identify some relevant ones and provide such information as well? Some examples are patient associations, relevant databases, OMIM/proteins/genes links, author's websites, etc...
5) As part of the EMBO Publications transparent editorial process initiative (see our Editorial at http://embomolmed.embopress.org/content/2/9/329), EMBO Molecular Medicine will publish online a Review Process File (RPF) to accompany accepted manuscripts. This file will be published in conjunction with your paper and will include the anonymous referee reports, your point-by-point response and all pertinent correspondence relating to the manuscript. Let us know whether you agree with the publication of the RPF and as here, if you want to remove or not any figures from it prior to publication. Please note that the Authors checklist will be published at the end of the RPF.
6) Please provide a point-by-point letter INCLUDING my comments as well as the reviewer's reports and your detailed responses (as Word file).

I look forward to reading a new revised version of your manuscript.

Yours sincerely,

Zeljko Durdevic

Zeljko Durdevic
Editor
EMBO Molecular Medicine

***** Reviewer's comments *****

Referee #3 (Remarks for Author):

I thank the authors for carefully addressing these concerns.

The authors performed the requested editorial changes.

3rd Revision - Editorial Decision 13th Sep 2022

We are pleased to inform you that your manuscript is accepted for publication and is now being sent to our publisher to be included in the next available issue of EMBO Molecular Medicine.

EMBO Press Author Checklist

Corresponding Author Name: Hesse/Taipaleenmäki
Journal Submitted to: EMBO Mol Med
Manuscript Number: EMM-2020-13617

USEFUL LINKS FOR COMPLETING THIS FORM
The EMBO Journal - Author Guidelines
EMBO Reports - Author Guidelines
Molecular Systems Biology - Author Guidelines
EMBO Molecular Medicine - Author Guidelines

Reporting Checklist for Life Science Articles (updated January 2022)
This checklist is adapted from Materials Design Analysis Reporting (MDAR) Checklist for Authors. MDAR establishes a minimum set of requirements in transparent reporting in the life sciences (see Statement of Task: 10.31222/osf.io/9sm4x). Please follow the journal's guidelines in preparing your manuscript.
Please note that a copy of this checklist will be published alongside your article.

Abridged guidelines for figures
1. Data
The data shown in figures should satisfy the following conditions:
- the data were obtained and processed according to the field's best practice and are presented to reflect the results of the experiments in an accurate and unbiased manner.
- ideally, figure panels should include only measurements that are directly comparable to each other and obtained with the same assay.
- plots include clearly labeled error bars for independent experiments and sample sizes. Unless justified, error bars should not be shown for technical replicates.
- if n<5, the individual data points from each experiment should be plotted. Any statistical test employed should be justified.
- Source Data should be included to report the data underlying figures according to the guidelines set out in the authorship guidelines on Data Presentation.

2. Captions
Each figure caption should contain the following information, for each panel where they are relevant:
- a specification of the experimental system investigated (eg cell line, species name).
- the assay(s) and method(s) used to carry out the reported observations and measurements.
- an explicit mention of the biological and chemical entity(ies) that are being measured.
- an explicit mention of the biological and chemical entity(ies) that are altered/varied/perturbed in a controlled manner.
- the exact sample size (n) for each experimental group/condition, given as a number, not a range;
- a description of the sample collection allowing the reader to understand whether the samples represent technical or biological replicates (including how many animals, litters, cultures, etc.).
- a statement of how many times the experiment shown was independently replicated in the laboratory.
- definitions of statistical methods and measures:
  - common tests, such as t-test (please specify whether paired vs. unpaired), simple χ2 tests, Wilcoxon and Mann-Whitney tests, can be unambiguously identified by name only, but more complex techniques should be described in the methods section;
  - are tests one-sided or two-sided?
  - are there adjustments for multiple comparisons?
  - exact statistical test results, e.g., P values = x but not P values < x;
  - definition of 'center values' as median or average;
  - definition of error bars as s.d. or s.e.m.

Please complete ALL of the questions below.
Select "Not Applicable" only when the requested information is not relevant for your study.

Materials

Newly Created Materials	Information included in the manuscript?	In which section is the information available? (Reagents and Tools Table, Materials and Methods, Figures, Data Availability Section)
New materials and reagents need to be available; do any restrictions apply?	Not Applicable	

Antibodies	Information included in the manuscript?	In which section is the information available? (Reagents and Tools Table, Materials and Methods, Figures, Data Availability Section)
For **antibodies** provide the following information:<br>- Commercial antibodies: RRID (if possible) or supplier name, catalogue number and or/clone number<br>- Non-commercial: RRID or citation	Yes	Materials and Methods

DNA and RNA sequences	Information included in the manuscript?	In which section is the information available? (Reagents and Tools Table, Materials and Methods, Figures, Data Availability Section)
**Short novel DNA or RNA including primers, probes**: provide the sequences.	Yes	Supplemental information

Cell materials	Information included in the manuscript?	In which section is the information available? (Reagents and Tools Table, Materials and Methods, Figures, Data Availability Section)
**Cell lines:** Provide species information, strain. Provide accession number in repository **OR** supplier name, catalog number, clone number, and/**OR** RRID.	Yes	Materials and Methods
**Primary cultures:** Provide species, strain, sex of origin, genetic modification status.	Yes	Materials and Methods
Report if the cell lines were recently **authenticated** (e.g., by STR profiling) and tested for mycoplasma contamination.	Yes	Materials and Methods

Experimental animals	Information included in the manuscript?	In which section is the information available? (Reagents and Tools Table, Materials and Methods, Figures, Data Availability Section)
**Laboratory animals or Model organisms:** Provide species, strain, sex, age, genetic modification status. Provide accession number in repository **OR** supplier name, catalog number, clone number, **OR** RRID.	Yes	Materials and Methods
**Animal observed in or captured from the field:** Provide species, sex, and age where possible.	Not Applicable	
Please detail **housing and husbandry conditions**.	Yes	Materials and Methods

Plants and microbes	Information included in the manuscript?	In which section is the information available? (Reagents and Tools Table, Materials and Methods, Figures, Data Availability Section)
**Plants:** provide species and strain, ecotype and cultivar where relevant, unique accession number if available, and source (including location for collected wild specimens).	Not Applicable	
**Microbes:** provide species and strain, unique accession number if available, and source.	Not Applicable	

Human research participants	Information included in the manuscript?	In which section is the information available? (Reagents and Tools Table, Materials and Methods, Figures, Data Availability Section)
If collected and within the bounds of privacy constraints report on age, sex and gender or ethnicity for all study participants.	Yes	Materials and Methods

Core facilities	Information included in the manuscript?	In which section is the information available? (Reagents and Tools Table, Materials and Methods, Figures, Data Availability Section)
If your work benefited from core facilities, was their service mentioned in the acknowledgments section?	Yes	Acknowledgment

Design

Study protocol	Information included in the manuscript?	In which section is the information available? (Reagents and Tools Table, Materials and Methods, Figures, Data Availability Section)
If study protocol has been **pre-registered, provide DOI in the manuscript**. For clinical trials, provide the trial registration number **OR** cite DOI.	Not Applicable	
Report the **clinical trial registration number** (at ClinicalTrials.gov or equivalent), where applicable.	Not Applicable	

Laboratory protocol	Information included in the manuscript?	In which section is the information available? (Reagents and Tools Table, Materials and Methods, Figures, Data Availability Section)
Provide DOI OR other citation details if **external detailed step-by-step protocols** are available.	Not Applicable	

Experimental study design and statistics	Information included in the manuscript?	In which section is the information available? (Reagents and Tools Table, Materials and Methods, Figures, Data Availability Section)
Include a statement about **sample size** estimate even if no statistical methods were used.	Yes	Materials and Methods
Were any steps taken to minimize the effects of subjective bias when allocating animals/samples to treatment (e.g. **randomization procedure**)? If yes, have they been described?	Yes	Materials and Methods
Include a statement about **blinding** even if no blinding was done.	Yes	Materials and Methods
Describe **inclusion/exclusion criteria** if samples or animals were excluded from the analysis. Were the criteria pre-established?<br><br>If sample or data points were omitted from analysis, report if this was due to attrition or intentional exclusion and provide justification.	Yes	Materials and Methods
For every figure, are **statistical tests** justified as appropriate? Do the data meet the assumptions of the tests (e.g., normal distribution)? Describe any methods used to assess it. Is there an estimate of variation within each group of data? Is the variance similar between the groups that are being statistically compared?	Yes	Materials and Methods + Figure Legends

Sample definition and in-laboratory replication	Information included in the manuscript?	In which section is the information available? (Reagents and Tools Table, Materials and Methods, Figures, Data Availability Section)
In the figure legends: state number of times the experiment was **replicated** in laboratory.	Yes	Figure Legends
In the figure legends: define whether data describe **technical or biological replicates**.	Yes	Figure Legends

Ethics

Ethics	Information included in the manuscript?	In which section is the information available? (Reagents and Tools Table, Materials and Methods, Figures, Data Availability Section)
Studies involving **human participants**: State details of **authority granting ethics approval** (IRB or equivalent committee(s), provide reference number for approval.	Yes	Materials and Methods
Studies involving **human participants**: Include a statement confirming that **informed consent** was obtained from all subjects and that the experiments conformed to the principles set out in the WMA Declaration of Helsinki and the Department of Health and Human Services Belmont Report.	Yes	Materials and Methods
Studies involving **human participants:** For publication of **patient photos**, include a statement confirming that consent to publish was obtained.	Not Applicable	
Studies involving experimental **animals**: State details of **authority granting ethics approval** (IRB or equivalent committee(s), provide reference number for approval. Include a statement of compliance with ethical regulations.	Yes	Materials and Methods
Studies involving **specimen and field samples:** State if relevant **permits** obtained, provide details of authority approving study; if none were required, explain why.	Not Applicable	

Dual Use Research of Concern (DURC)	Information included in the manuscript?	In which section is the information available? (Reagents and Tools Table, Materials and Methods, Figures, Data Availability Section)
Could your study fall under dual use research restrictions? Please check biosecurity documents and list of **select agents and toxins** (CDC): https://www.selectagents.gov/sat/list.htm	Not Applicable	
If you used a select agent, is the security level of the lab appropriate and reported in the manuscript?	Not Applicable	
If a study is subject to dual use research of concern regulations, is the name of the **authority granting approval and reference number** for the regulatory approval provided in the manuscript?	Not Applicable	

Reporting

The MDAR framework recommends adoption of discipline-specific guidelines, established and endorsed through community initiatives. Journals have their own policy about requiring specific guidelines and recommendations to complement MDAR.

Adherence to community standards	Information included in the manuscript?	In which section is the information available? (Reagents and Tools Table, Materials and Methods, Figures, Data Availability Section)
State if relevant guidelines or checklists (e.g., **ICMJE, MIBBI, ARRIVE, PRISMA**) have been followed or provided.	Not Applicable	
For **tumor marker prognostic studies,** we recommend that you follow the **REMARK** reporting guidelines (see link list at top right). See author guidelines, under 'Reporting Guidelines'. Please confirm you have followed these guidelines.	Not Applicable	
For **phase II and III randomized controlled trials**, please refer to the **CONSORT** flow diagram (see link list at top right) and submit the CONSORT checklist (see link list at top right) with your submission. See author guidelines, under 'Reporting Guidelines'. Please confirm you have submitted this list.	Not Applicable	

Data Availability

Data availability	Information included in the manuscript?	In which section is the information available? (Reagents and Tools Table, Materials and Methods, Figures, Data Availability Section)
Have **primary datasets** been deposited according to the journal's guidelines (see 'Data Deposition' section) and the respective accession numbers provided in the Data Availability Section?	Yes	Data Availability Section
Were **human clinical and genomic datasets** deposited in a public access-controlled repository in accordance to ethical obligations to the patients and to the applicable consent agreement?	Not Applicable	
Are **computational models** that are central and integral to a study available without restrictions in a machine-readable form? Were the relevant accession numbers or links provided?	Not Applicable	
If publicly available data were reused, provide the respective **data citations in the reference list**.	Not Applicable	
